# ﻿A revision of *Lycianthes* (Solanaceae) in tropical Asia

**DOI:** 10.3897/phytokeys.245.121988

**Published:** 2024-07-29

**Authors:** Sandra Knapp

**Affiliations:** 1 Natural History Museum, Cromwell Road, London SW7 5BD, UK Natural History Museum London United Kingdom

**Keywords:** Asia, conservation assessments, ochlospecies, subtropics, tropics, weeds, widespread species

## Abstract

The genus *Lycianthes* (Dunal) Hassl. (Solanaceae) has in the past been treated as a section of the large genus *Solanum* L. but is more closely related to *Capsicum* L. Outside of the Americas, where the highest species diversity occurs, the genus is found in tropical and subtropical habitats from India to Japan and the Philippines, including the islands of Indonesia, New Guinea and the Solomons. The 19 species from Australia, New Guinea and the Pacific were treated in ‘PhytoKeys 209’. Here I treat the remaining 10 species occurring across Asia; including two native species, *L.biflora* (Lour.) Bitter and *L.oliveriana* (Lauterb. & K.Schum) Bitter, and one cultivated species, *L.rantonnetii* (Carrière) Bitter that were also included in the earlier work. The Asian species treated here occupy a wide range of forested and disturbed habitats and are diverse in habit, ranging from epiphytic vines to small or medium sized trees, shrubs or creeping herbs. Many of the species are weedy plants of highly disturbed habitats and are best characterised as “ochlospecies”, with complex polymorphic variation. *Lycianthesrantonnetii*, a species native to southern South America, is recorded as cultivated in India and Pakistan, but may be more widespread than collections indicate. The history of taxonomic treatments of *Lycianthes* in Asia is discussed, along with details of morphology found in all species. All species are treated in full, with complete morphological descriptions, including synonymy, lecto- or neotypifications, discussions of ecology and vernacular names, distribution maps and preliminary conservation assessments (for all except the cultivated *L.rantonnetii*). Searchable lists of all specimens examined are presented as Suppl. materials 1, 2.

## ﻿﻿Introduction

*Lycianthes* (Dunal) Hassl. is the third largest genus in the Solanaceae, after *Solanum* L. and *Cestrum* L. (Dean et al. 2020). The genus comprises approximately 150 to 200 species, most of these distributed in the Americas, with the centre of species diversity in Mesoamerica. Significant species diversity is present outside of the Americas however, with New Guinea, the world’s most species-rich tropical island (Bitter 1917; Symon 1984a, b; Cámara-Leret et al. 2020), standing out as an area of high endemism, as has been seen for many other taxa (Knapp 2022). Previously considered a section of *Solanum* L., the genus *Lycianthes* has been shown to be more closely related to the peppers (*Capsicum* L.) despite sharing the striking character of poricidal anthers with *Solanum* (Särkinen et al. 2013).

*Lycianthes* can be recognised by the combination of axillary inflorescences, poricidal anthers and a calyx that does not have distinct lobes, but rather has a truncate rim (sometimes called a sleeve) with or without tooth-like appendages protruding from near or below the calyx rim (D’Arcy 1986). Species of *Lycianthes* can be confused with other genera with similar unlobed calyces like *Capsicum*, *Brachistus* Miers, *Cuatresia* Hunz., and *Witheringia* L’Her. (D’Arcy 1986; Dean et al. 2020), especially when flowers are lacking.

This revision is the second in a series of treatments of the species of *Lycianthes* outside the Americas, and part of collaborative work to treat the species of this genus worldwide (e.g., Dean et al. 2020; Knapp 2022). In this revision I treat *Lycianthes* in the broad area of tropical Asia (see Table 1 for a list of the species and their distribution) from India to Japan but excluding the island of New Guinea that has been treated separately (Knapp 2022) as all but two (*L.biflora*, *L.oliveriana*) of the species occurring there are endemic. I have included Pakistan here, as the cultivated species *L.rantonnetii* occurs there.

**Table 1.** Species of *Lycianthes* occurring in Asia, the Pacific and Australia. Species treated in this monograph are in bold font; more detailed distributions for New Guinea species can be found in Knapp (2022).

Species	Distribution	Reference
*Lycianthesbambusarum* (Bitter) Bitter	New Guinea	Knapp (2022)
***Lycianthesbanahaensis* (Elmer) Bitter**	**Indonesia to Philippines**	**treated here**
*Lycianthesbelensis* (Merr. & L.M.Perry) A.R.Bean	New Guinea	Knapp (2022)
***Lycianthesbiflora* (Lour) Bitter**	**Widespread throughout**	Knapp (2022); treated here
***Lycianthesbimensis* (Miq.) Bitter**	**Indonesia**	**treated here**
*Lycianthesbitteriana* (Symon) A.R.Bean	New Guinea	Knapp (2022)
*Lycianthescladotrichota* (Bitter) Bitter	New Guinea	Knapp (2022)
*Lycianthesdendropilosa* (Symon) A.R.Bean	New Guinea	Knapp (2022)
*Lycianthesimpar* (Warb.) Bitter	New Guinea	Knapp (2022)
*Lyciantheskaernbachii* (Lauterb. & K.Schum.) Bitter	New Guinea	Knapp (2022)
***Lyciantheslaevis* (Dunal) Bitter**	**Widespread throughout**	**treated here**
*Lyciantheslucens* S.Knapp	New Ireland	Knapp (2022)
***Lyciantheslysimachioides* (Wall.) Bitter**	**Widespread throughout**	
*Lycianthesmoszkowskii* (Bitter) Bitter	New Guinea	Knapp (2022)
*Lycianthesmultifoliola* (Merr. & L.M.Perry) A.R.Bean	New Guinea	Knapp (2022)
***Lycianthesoliveriana* (Lauterb. & K.Schum.) Bitter**	**New Guinea, Maluku islands of Indonesia**	**Knapp (2022); and treated here**
***Lycianthesparasitica* (Blume) Bitter**	**Thailand to Philippines**	**treated here**
*Lycianthesperanomala* (Wernham ex Rdl.) A.R.Bean	New Guinea	Knapp (2022)
***Lycianthesrantonnetii* (Carrière) Bitter**	**Introduced; in drier areas**	**Barboza et al. (2013); Knapp (2022)**
*Lycianthesrostellata* (Merr. & L.M. Perry) A.R.Bean	New Guinea	Knapp (2022)
***Lycianthesschizocalyx* (Merr.) Bitter**	**Thailand to Philippines**	**treated here**
*Lycianthesshanesii* (F.Muell.) A.R.Bean	Australia	Knapp (2022)
***Lycianthesshunningensis* C.Y. Wu & S.C.Huang**	**Northeastern India to China and Indochina**	**treated here**
*Lycianthesvitiensis* (Seem.) A.R.Bean	Bougainville to Tonga (Pacific)	Knapp (2022)
*Lyciantheswollastonii* (Wernham) A.R.Bean	New Guinea	Knapp (2022)

## ﻿﻿Phylogenetic and taxonomic history

Taxa now recognised as *Lycianthes* were first included in the large and diverse genus *Solanum*, due to the shared poricidal anthers. Dunal (1813, 1816) recognised them as his group “Polymeris” based on the calyx appendages and axillary flowers. This group was maintained by subsequent authors (see summary in Dean et al. 2020), eventually being recognised at the subgeneric level by the German botanist Georg Bitter (Bitter 1917) in a treatment of *Solanum* in the Papuan region.

In the same year the group was distinguished as a distinct genus by Émile (or Emil) Hassler (Hassler 1917), including only a few of the species in Dunal’s larger group (Dunal 1852) that contained stone cells in their fruits, all from the Americas. Bitter (1919) later recognised the distinctness of *Lycianthes* at the generic level and his worldwide monograph of the genus contained 189 taxa (species and infraspecies), most transferred from *Solanum*. He suggested the genus was closely related to *Capsicum* based on their similar calyx morphology; this suggestion, however, was not widely accepted until the advent of molecular phylogenetic studies in the Solanaceae (see below). Throughout the rest of the 20^th^ century various authors continued to treat the species of *Lycianthes* as members of *Solanum* (e.g., Morton 1976; Hunziker 1979; Symon 1981, 1985), or as a distinct genus related to *Solanum* (e.g., Hunziker 2001).

Phylogenetic analyses of Solanaceae using DNA sequence data showed that the species of *Lycianthes* were indeed most closely related to *Capsicum* and not to *Solanum* (e.g., Särkinen et al. 2013); few species from outside the Americas were included in this analysis. These and other more focused studies on the two genera appeared to suggest that *Lycianthes* was paraphyletic with respect to *Capsicum* (Spalink et al. 2018), but considerable discordance between different genomic regions complicates resolution of the monophyly of *Lycianthes*. Its close relationship to *Capsicum*, however, is now undisputed. Recent studies using whole plastome sequences and more numerous nuclear sequences (A. Orejuela, pers. comm.) support the reciprocal monophyly of *Capsicum* and *Lycianthes*; these more recent studies have included more species of *Lycianthes* from outside the Americas (Orejuela 2021).

**Figure 1.**
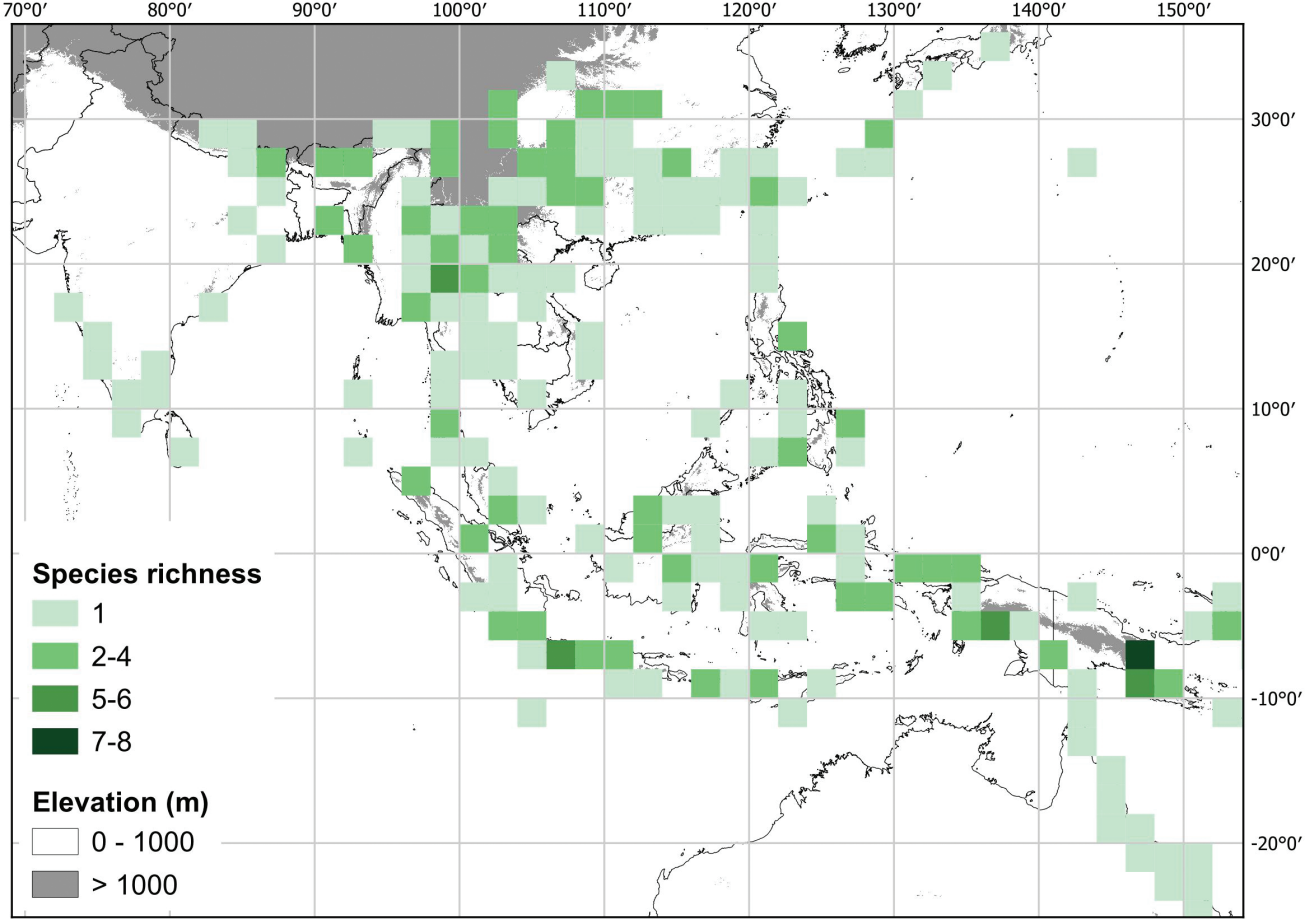
Distribution of species of *Lycianthes* native outside of the Americas (including all species treated in Knapp 2022). Darker squares indicate those with higher species richness. The cultivated *L.rantonnetii* is not included here.

Dean et al. (2020) summarised the taxonomic history of *Lycianthes* in the Americas; taxa outside the Americas have been less intensively studied. Twenty-four native species of *Lycianthes* (see list in Table 1) occur across Asia from northern India to Japan, the Philippines and New Guinea, to the Pacific islands of Fiji and Samoa, including tropical areas of northern Australia (see Fig. 1).

Treatments of the Asian species of *Lycianthes* have mostly been done at a regional level as part of national or local floras. The first Asian *Lycianthes* species to be described was *L.biflora* (as *Solanumbiflorum* Lour.) from “Cochinchina” (Loureiro 1790). Blume (1823, 1826) and Miquel (1864) treated species from Indonesia (as part of *Solanum*), recognising four and six species respectively. The most comprehensive early treatment of Asian species was done by Christian Godfrey Nees van Esenbeck (1834) in his “Monograph of the East Indian Solaneae”, treating the taxa found in what was then “British India” (comprising Pakistan, India, Myanmar, Bangladesh and Sri Lanka). In a set of papers read at the Linnean Society of London in 1832 he described 12 species (as *Solanum*, as had earlier authors) differentiating them mostly by calyx morphology (Nees van Esenbeck 1834). He validated many names used by the Danish botanist Nathaniel Wallich whose collections for the British East India Company between 1829 and 1847 resulted in an unpublished list (the “Wallich Catalogue” correctly titled “A numerical list of dried specimens of plants in the East India Company’s Museum, collected under the superintendence of Dr. Wallich of the Company’s Botanic Garden at Calcutta” https://wallich.rbge.org.uk/) that was widely distributed in the early part of the 19^th^ Century (De Candolle and Radcliffe-Smith 1981). Subsequent local treatments for the region (Clarke 1883; Hepper 1987) largely used the names as circumscribed by Nees van Esenbeck (1834).

In the early 20^th^ Century, the botanists Adolph Elmer and Elmer Merrill worked intensively on the Philippine flora, and in a series of papers (Elmer 1908, 1910, 1915; Merrill and Merritt 1910; Merrill 1912) described a number of species (all as *Solanum*), largely differentiating them on the basis of where in the archipelago they were collected.

The German botanist Georg Bitter published a worldwide monograph of *Lycianthes* (Bitter 1919) in which he recognised 133 species and many infraspecific taxa, often in hierarchies that reflected his idea of classification. For Asia he recognised 29 species, 8 subspecies, 23 varieties and a single form. Ten of these species were endemic to the island of New Guinea or the Pacific; Bitter’s taxon concepts were often driven by geography, with varieties consisting of specimens from a single area (e.g., L.macrodon(Nees)Bittervar.sikkimensis Bitter, *L.macrodon* (Nees) Bitter) var. manipurensis Bitter). As collecting has increased, these regional differences have become less clear (as Bitter predicted, see below).

Recent floristic treatments have generally recognised few species. Hepper (1987) recognised a single species for Sri Lanka (*L.bigeminata* (Nees) Bitter, here a synonym of *L.laevis*). For China Zhang et al. (1994) recognised a number of Bitter’s (1919) taxa at the species level, and in the course of work on the flora described several more species and infraspecific taxa (Wu and Huang 1978), often from very few specimens. In total Zhang et al. (1994) recognised 15 taxa (10 species and 5 varieties). In treating *Lycianthes* for Bhutan, Mill (2001) recognised only two species, *L.macrodon* and *L.crassipetala* (Wall.) Bitter; he distinguished *L.macrodon* from *L.biflora* by the length of the calyx appendages, and *L.crassipetala* from *L.laevis* by its distribution (Himalayas versus southern India). In the flora of Cambodia, Laos and Vietnam Hul and Dy Phon (2014) recognised four species of *Lycianthes* (including the then recently described *L.baviensis* V.V.Hop, here treated as a synonym of *L.schizocalyx*).

Several of the Asian species of *Lycianthes* treated here are widespread and taxonomically very difficult (e.g., *L.biflora*) resulting in a plethora of names at both species and infraspecies ranks. Bitter (1919: 461) recognised what he called a “Gesamart” (Overall species or Super species sensu Mayr 1992) for taxa he considered related to *L.biflora* [*L.biflora*, *L.macrodon*, *L.schizocalyx*, *L.brachyanthera* Bitter, *L.minutipila* Bitter, *L.denticulata* (Nees) Bitter]. He admitted that the boundaries between these taxa were not at all clear; “the demarcation from some of the following is by no means completely sharp, even if these are also some sorts apparently easier to distinguish from *L.biflora* due to the smaller number of calyx lobes. Because of its wide distribution, the main species *L.biflora* is particularly diverse; much lack of clarity about differences and similarities can only be decided after comparative cultivation and a careful examination of the history of the development of the individual forms.” (translated from the original German by SK). He also suggested that “it is, however, very possible that, after becoming aware of even more abundant evidence, the scope of this large species must undergo a considerable expansion.” (Bitter 1919: 416, translated from the original German).

*Lycianthesbiflora* is indeed a very complicated species; I have had difficulty demarcating clear boundaries between what appear to be very different extremes. This is also the case for the similarly variable and widespread *L.laevis* (see species treatments) and to some extent also *L.schizocalyx*. These are perhaps best considered ochlospecies (sensu the late Frank White as described in Cronk 1998), recognised as complexes where variation is polymorphic but not geographically or ecologically correlated, and characters appear to vary independently when specimens are analysed over the entire range of the taxon. Variants are often locally distinct, but over the entire range gradation is continuous. These species are usually widespread, and Cronk (1998) suggests that such patterns may be the result of disturbance-loving species with wide ecological tolerances rapidly invading new areas, with local mutations or adaptations arising in a geographically unstructured way. *Solanumumbelliferum* Eschsch. (DulMo clade, sensu Gagnon et al. 2022) of the California Floristic Province is such a species. Parish (1901) used variation in pubescence as the key character for distinguishing his taxa but also admitted that this character was continuous, while also stating “satisfactory segregation is a matter of no little difficulty. The exercise of that botanical industry that multiplies “species” by the minute description of individuals might reap here an abundant harvest.” I chose to treat *S.umbelliferum* as a single very variable taxon and urged further work at the population level (Knapp 2013), while others have treated it differently (Keil 2018). A similar situation may occur here.

The widespread, highly polymorphic *Lycianthes* species I have recognised here are excellent candidates for further in-depth field, population and molecular study. Widespread weedy taxa such as these are often ignored or treated as of little interest, but they are sources of genetic variation and can been seen as ‘pumps’ for the processes of sympatric, parapatric or allopatric speciation. Details of local variation can be found in the individual species treatments.

## ﻿﻿Morphology

### ﻿Habit and stems

**Figure 2.**
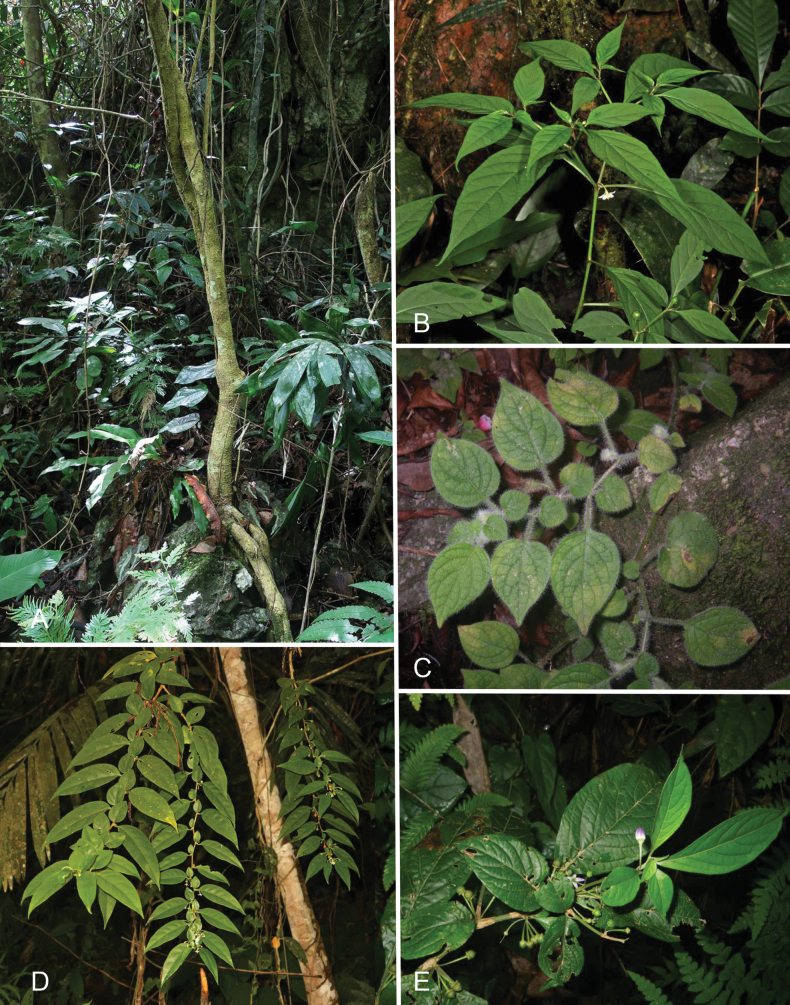
Habit and leaves of *Lycianthes* in Asia **A***L.banahaensis* (*Rule s.n.*, Philippines; DOL134625) **B***L.biflora* (*Pelser & Barcelona s.n.*, Philippines; DOL44707) **C***L.lysimachioides* (*Nuraliev 1638*, Vietnam) **D***L.parasitica* (*Saavedra s.n.*, Philippines; DOL145620) **E***L.shunningensis* (*Nuraliev 2810*, Vietnam). Photograph credits: **A** Greg Rule **B** Pieter B. Pelser & Julie F Barcelona **C, E** Maxim Nuraliev **D**) Aljohn J. Saavedra. DOL numbers are identifiers from PhytoImages (https://phytoimages.siu.edu). Details of collecting localities can be found in Suppl. material 2.

All species treated here are perennial plants, most of them woody, at least at the base. *Lyciantheslysimachioides* is usually a creeping herb, but can be a small shrub; the stems, however, are always herbaceous (Fig. 2C). *Lycianthesbiflora*, *L.laevis*, *L.schizocalyx* and *L.shunningensis* are all shrubs usually less than 2 m in height (Fig. 2B, E). Their distal stems are often described as spreading over other vegetation. Both *L.banahaensis* and *L.bimensis* are small trees, often attaining heights of 15 metres (Fig. 2A). *Lycianthesoliveriana* similarly has large woody stems, but is a liana, usually growing high in the canopy. Herbarium labels often erroneously describe it as a shrub or tree (Knapp 2022). *Lycianthesparasitica* is a true epiphyte (Fig. 2D), an unusual habit that has evolved several times across the Solanaceae (Orejuela 2021). Like *L.oliveriana*, it is often described as a shrub or tree on herbarium labels; whether this is due to these plants sometimes growing as shrubs or trees will need field confirmation.

Growth in the Solanaceae is initially monopodial, but with the onset of flowering, becomes sympodial. Inflorescences terminate each branch, and stem growth continues from an axillary bud or buds, as also seen in species of *Capsicum* and American *Lycianthes* (Dean et al. 2020; Barboza et al. 2022). Growth can either be from a single axillary bud, forming a monochasial branching pattern (as seen in most species of *Solanum* and in many the taxa treated here, e.g., Fig. 2C, D) or from two axillary buds, forming a dichasial branching pattern (common in *Capsicum* and in several of the species here, Fig. 2B). Each lateral shoot with alternate leaves arranged in a 1/3 phyllotaxic spiral and a terminal inflorescence (segment of stem between each inflorescence) is termed the sympodial unit in the descriptions here. Danert (1958, 1967, 1970) showed that when growth of the axes is suppressed, the leaves appear paired (geminate) at a node, thus the arrangement of leaves along the stems of Solanaceae was due to concaulescence of sympodial units, such that leaves can appear either singly or in groups. The number and arrangement of leaves in each sympodial unit can be diagnostic for both clades and species (e.g., Knapp 2002; Peralta et al. 2008). All of the taxa treated here have difoliate sympodial units with leaves usually strongly paired (geminate) at the nodes (Fig. 2A, B); these paired leaves are either similar in both size and shape, or similar in shape but differing in size (e.g., *L.parastica*, Fig. 2D). Some species have strongly zig-zagging stems (e.g., *L.parasitica*, Figs 2D, 18) resulting from the angle change of stems during sympodial growth.

### ﻿Leaves

Leaves of *Lycianthes* are all simple and entire, but occasionally some plants of *L.laevis* have at least some leaves with incised margins (see species description; Fig. 12). Many of the species treated here have geminate sympodial units (see above), with the major and minor leaves of different size but usually not of different shape (see Fig. 2B, E) but some in some species (e.g., *L.parasitica*, Fig. 2D and sometimes *L.lysimachioides*, Fig. 2C) paired leaves are remarkably different in both size and shape (see illustrations of individual species). In these species, the minor leaves are sometimes lost, giving the appearance of non-geminate sympodial units, and has led to description of new taxa based on apparent lack of these minor leaves (e.g., *S.epiphyticum* Merr., a synonym of *L.parasitica*). In the species descriptions below, I have described the major (larger) leaves first, followed by the minor leaves. The minor leaves in many species are deciduous as branches age, or in drying.

### ﻿Pubescence

Trichome morphology can be very useful in Solanaceae taxonomy (see Seithe 1962, 1979; Knapp 2001) with type, density, and colour often diagnostic for species identification. Stellate trichomes, important for identification of solanums in the Leptostemonum clade Vorontsova and Knapp 2016; Aubriot and Knapp 2022) and in American *Lycianthes* (Dean et al. 2020) are not found in Asian species of *Lycianthes*. Pubescence in these taxa is of uniseriate trichomes, either unbranched or dendritic (branched like deer antlers). Trichome type and density varies between and within species in Asia; the widespread species *L.biflora*, *L.laevis* and *L.schizocalyx* are all very variable in pubescence density. Only *L.biflora* has dendritic trichomes, these often rather sparse and intermixed with simple ones. I have been unable to make clear distinctions between the trichome types and each pubescence variation grades into the other throughout the range of each of these widespread taxa. All of the species treated here have tiny papillate trichomes on the new growth, these are common on most Solanaceae and are not the main types of trichomes useful for species differentiation.

### ﻿Inflorescences

Inflorescences of all species of *Lycianthes* are found in the leaf axils, often as small fascicles of only a few flowers (Dean et al. 2020) but sometimes with many flowers arising from an enlarged cushion-like structure (Knapp 2022) or borne along a short congested rhachis (Fig. 5D). The axillary inflorescence is one unequivocal character distinguishing *Lycianthes* from *Solanum* (although see Knapp and Helgason 1997; the Pteroidea clade of *Solanum* has axillary inflorescences, but these are never fasciculate, and the calyx is clearly 5-lobed).

**Figure 3.**
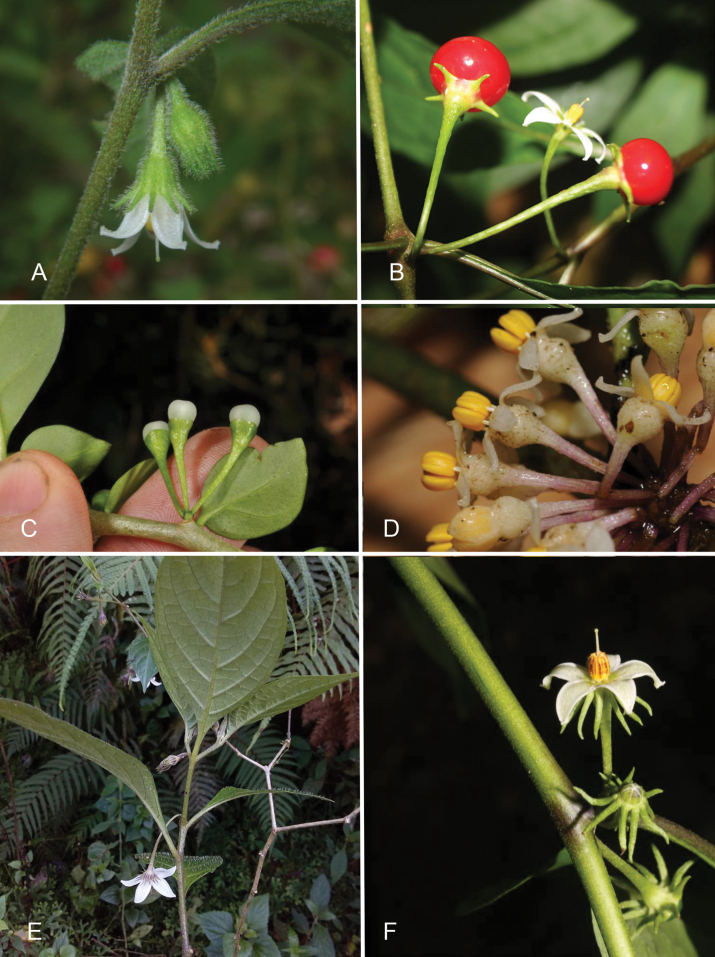
Calyx appendage variation of *Lycianthes* in Asia **A***L.biflora* with 10 equal calyx appendages (*Nuraliev s.n*, Vietnam [no voucher]) **B***L.laevis* with 5 calyx appendages (*Mustaqim s.n.*, Indonesia; DOL207886) **C***L.parasitica* with small, nub-like calyx appendages (*Pelser & Barcelona s.n.*, Philippines; DOL84744) **D***L.oliveriana* with thick, warty calyces with no appendages (*Damas et al. SAJ-1050*, Papua New Guinea) **E***L.schizocalyx* with long flower pedicels and 10 unequal calyx appendages (*Heyer s.n.*, Philippines; DOL151906) **F***L.shunningensis* with 10 strongly reflexed calyx appendages (*Averyanov 887*, Laos). Photograph credits: **A** Maxim Nuraliev **B** Wendy Mustaqim **C** Pieter B. Pelser & Julie F. Barcelona **D** Shelley James **E** Philippe Heyer **F** Leonid Averyanov. DOL numbers are identifiers from PhytoImages (https://phytoimages.siu.edu). Details of collecting localities can be found in Suppl. material 2.

The number of flowers per fascicle is not only variable between species (only one or two in *L.lysimachioides* versus up to 20 or even more in *L.oliveriana*, Figs 2C, 3D), but also within species, depending on the age of the inflorescence (see individual species illustrations). In young plants it can appear there are few flowers in each inflorescence (e.g., *L.biflora*, *L.laevis*); the number of flowers per fascicle in these taxa is not a reliable character for species level identification.

### ﻿Calyces

**Figure 4.**
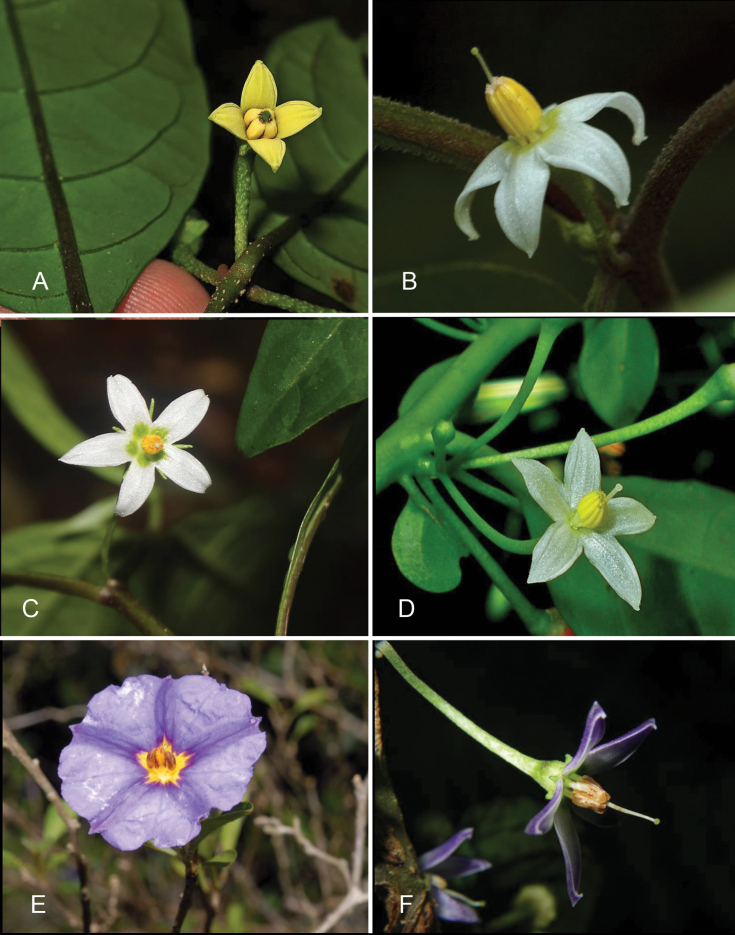
Flowers of *Lycianthes* in Asia **A***L.banahaensis* with tetramerous yellow flowers, note the stigma sitting almost within the anther tube (*Rule s.n.*, Philippines; DOL134710) **B***L.biflora* with membranous corolla lobes and a minutely capitate stigma (*Knapp et al. 10106*, China) **C***L.laevis* flower with green blotches at the base of the corolla lobes (*Mustaqim s.n*., Indonesia; DOL207828) **D***L.parasitica* flower with spreading corolla lobes and capitate stigma (*Tandang s.n.*, Philippines; DOL76576597) **E***L.rantonnetii* rotate corolla with orange, slightly curved anthers (*Stevenson s.n.*, cultivated in Mexico; DOL183568) **F***L.shunningensis* with very short reflexed calyx appendages and cucullate corolla lobe tips (*Nuraliev et al. 2810*, Vietnam). Photograph credits: **A** Greg Rule **B** Sandra Knapp **C** Wendy Mustaqim **D** Danilo Tandang; **E** Dennis Stevenson **F** Maxim Nuraliev. DOL numbers are identifiers from PhytoImages (https://phytoimages.siu.edu). Details of collecting localities can be found in Suppl. material 2.

**Figure 5.**
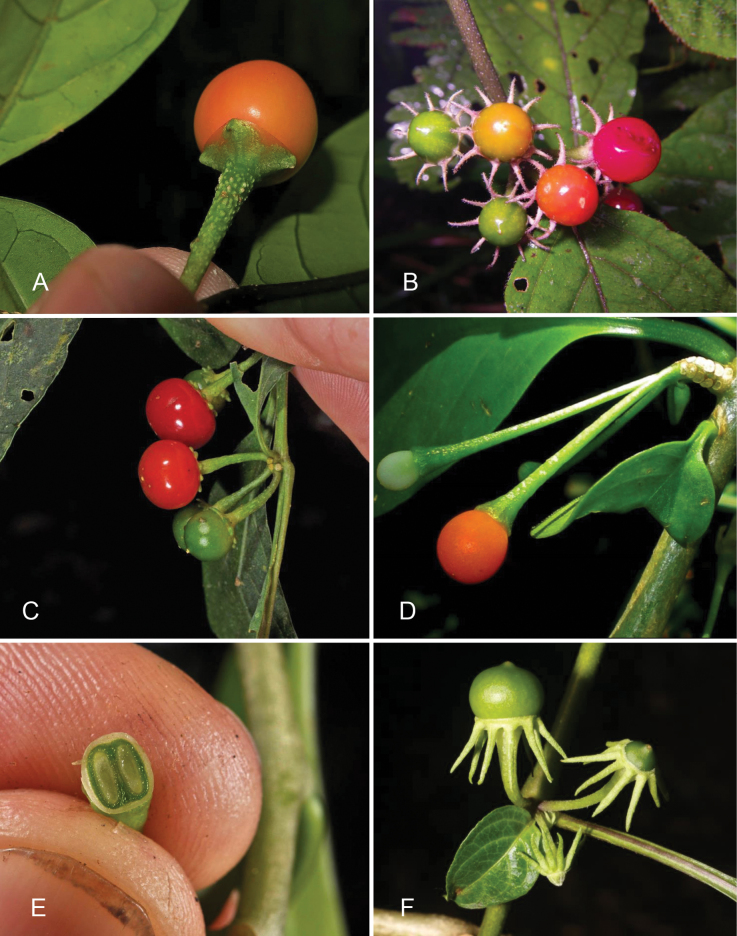
Fruits of *Lycianthes* in Asia **A***L.banahaensis* showing the opaque pericarp, lenticellate pedicel and small nub-like calyx appendages (*Rule s.n.*, Philippines; DOL134667) **B***L.biflora* showing sequence of fruiting ripening and elongate calyx appendages (*Nuraliev 182a*, Vietnam) **C***L.biflora* plant with short calyx appendages and little pubescence (*Pelser & Barcelona s.n*., Philippines; DOL44710) **D***L.parasitica* showing the small inflorescence axes and berries changing from white to orange with ripening (*Tandang s.n.*, Philippines; DOL76584) **E***L.parasitica* cross-section of berry showing the two seeds (*Pelser & Barcelona s.n.*, Philippines; DOL84746) **F***L.shunningensis* showing unripe berry and strongly reflexed calyx appendages (*Averyanov et al. 887*, Laos). Photograph credits: **A** Greg Rule **B** Maxim Nuraliev **C, E** Pieter B. Pelser & Julie F. Barcelona **D** Danilo Tandang **F** Leonid Averyanov. DOL numbers are identifiers from PhytoImages (https://phytoimages.siu.edu). Details of collecting localities can be found in Suppl. material 2.

In all species of *Lycianthes* the calyx is synsepalous and unlobed (D’Arcy 1986; Dean et al. 2020). The cup-like calyx tube has an entire truncate rim, although in many species (e.g., *L.schizocalyx*) the hyaline rim appears somewhat irregularly lobed through tearing. Calyx appendages emerge from below the truncate rim or from very near it; there is often a small margin of tissue between the appendage and the edge of the rim. In *L.biflora*, for example, the appendages appear to rise directly from the calyx rim (Fig. 3A) while in *L.schizocalyx* and *L.shunningensis* (Figs 3E, F, 4F) there is always a distinct rim of tissue for at least some of the appendages. In *L.shunningensis* the appendages are bent backwards such that they are reflexed and parallel to the pedicel (Figs 3F, 5F); this character can be difficult to see in dried specimens if they have not been pressed soon after collection. Calyx appendages can vary considerably in length within species and even within a single plant, they have previously been used as key taxonomic characters, but variability appears to be more or less continuous. In bud, the appendages appear longer in relation to overall calyx size, but as flowers expand the appendages become less apparent. For example, Wallich specimens identified as “Solanummembranaceum” have early buds with calyx appendages as long as the calyx tube, whereas other specimens of these plants in flower or fruit have much shorter calyx appendages relative to the length of the calyx tube.

### ﻿Corollas

The *Lycianthes* corolla is sympetalous, with 5 (occasionally 4) lobes (Fig. 4). Corolla shape varies from deeply stellate to rotate; deeply stellate corollas are divided nearly to the base and rarely have thin interpetalar tissue connecting the lobes (e.g., *L.biflora*, also see Fig. 4B, C, D, F)), while rotate corollas have abundant interpetalar tissue (e.g., *L.rantonnetii*, Figs 4E, 20). The only species occurring in this region with rotate corollas is the cultivated *L.rantonnetii* but in the Americas many species of *Lycianthes* have this corolla morphology (Dean 2001; Dean et al. 2020). *Lycianthesbanahaensis* is characterised by having many populations with tetramerous flowers (Fig. 4A), but this is not completely consistent across the species range. The tips of the corolla lobes are cucullate in most of these species (Figs 4A, F, 5B, C, F).

Interpetalar tissue, if present, is thinner than that of the lobes proper, is usually folded within the bud before anthesis, and usually lacks any pubescence such as that found on the rest of the abaxial corolla lobe surfaces. The native species treated here lack copious interpetalar tissue, where it does occur it is usually a thin flap at the margins of somewhat fleshy corolla lobes (e.g., *L.banahaensis*, *L.bimensis*; Fig. 4A).

Corolla colour in these species varies from yellow (many collections of *L.banahaensis* Fig. 4A) to white to dark purple (Figs 3, 4), with many species having individuals varying in overall flower colour or with patterns of colour such as the midveins of corolla lobes darker than the rest of the tissue (e.g., *L.laevis*). Several of these species often have green spots at the base of the corolla lobes (see Fig. 4C) similar to those found in some species of *Solanum* (Knapp 2013; Knapp et al. 2019, 2023). Reliance on corolla colour for identification is not recommended.

### ﻿Androecium

Stamens of *Lycianthes* are ‘Solanum-type’ (sensu Endress 1996) and specialised for buzz-pollination by bees (see Figs 3, 4). The anthers are usually longer than the filaments and dehisce by terminal pores. Bees grasp the anther cone and engage their indirect flight muscles to set up a vibration that causes pollen to be ejected from the anther theca through the pore (Buchmann et al. 1977; Vallejo-Marín et al. 2010). Endress (1996) has suggested that the poricidal nature of anthers is a secondarily acquired feature in plants with dry pollen, since vibratile pollination occurs in many plants without poricidal anthers. Most species of *Lycianthes* treated here have ellipsoid anthers, but these are usually elongated into a tiny beak of tissue that is paler in dry material. The anthers of some specimens of *L.biflora* are pubescent, with simple uniseriate trichomes along the dehiscence zone or over the entire anther surface. These hairs are reminiscent to those found in members of the Tomato clade of *Solanum* (Peralta et al. 2008), that hold the anthers together in a connivent structure that aids pollination. None of the other species treated here has pubescent or papillate anthers.

Terminal anther pores in the taxa treated here are always distally directed but are of two distinct shapes. Pores are either circular and distinctly bounded, or somewhat tear-drop shaped, with a line of dehiscence extending between the anther locules for varying lengths. In most of the taxa treated here the pore is tear-drop shaped (e.g., *L.biflora*, see Fig. 4B, F) the terminal anther pores elongate as the flower ages, and the anthers appear to “unzip” (D’Arcy et al. 1996; Knapp 2002) and become somewhat longitudinally dehiscent. In species with circular pores (e.g., *L.oliveriana*, Fig. 3D), the anther pore does not change shape with flower age and remains a distinct round opening at the distal tip. When anthers dehisce the pore or other shaped opening is edged with white crystals which have been described as the “oxalate package” (D’Arcy et al. 1996). These crystals have been implicated in anther dehiscence but are not differently placed in species with poridical anthers (see D’Arcy et al. 1996).

Unlike the heterantherous androecia of many American *Lycianthes* species (see Dean et al. 2020), all native species occurring in the region have stamens, anthers, and filaments of equal length. The bright orange, slightly curved anthers of the cultivated *L.rantonnetii*, however, are borne on filaments of different lengths, two short and three long (see description and Fig. 4E).

Filaments in most of the species treated here are glabrous, but the cultivated *L.rantonnetii* and many populations of *L.biflora* have tangled weak-walled simple trichomes on the adaxial surfaces of the filaments (inside the stamen tube).

### ﻿Gynoecium

The gynoecium in *Lycianthes* is bicarpellate with axile placentation. The ovary is glabrous, and usually conical to globose. The flowers lack nectaries, as do all species of *Lycianthes*. In species with heterostylous flowers the ovary in short-styled flowers is vestigial (and the flowers functionally staminate). In long-styled flowers the style is usually exserted from the anther cone, but in short-styled flowers it is contained well within, although in *L.banahaensis* the style is never exserted, even in long-styled flowers (Fig. 4A). The style is usually straight and glabrous, only in the cultivated *L.rantonnetii* is it strongly curved. The stigma is either very minutely capitate and merely a slight enlargement of the style tip (e.g., *L.biflora*, Fig. 4B), larger and more obviously capitate (e.g., *L.parasitica*, Fig. 4D) or elongate-clavate (e.g., *L.bimensis*); occasionally the stigma is bilobed (e.g., *L.bimensis*, *L.oliveriana*). The ovules are anatropous and non-arillate. The stigma of long-styled flowers of *L.banahaensis* is held almost at the level of the anther pores (Fig. 4A).

### ﻿Fruits

In *Lycianthes*, the fruit is a globose to subglobose to ellipsoid berry. In the species treated here, berries are globose to subglobose in most taxa. In cultivation *L.rantonnetii* rarely sets fruit, but in its native range the berries are large and ellipsoid (see Barboza 2013). Berry colour at maturity in *Lycianthes* can be green, yellow, orange, red, blue or dark purplish black (see Dean et al. 2020), and all are green when immature (Fig. 5F). While most of the species here have soft, fleshy berries, those of *L.oliveriana* appear to remain hard through maturity (they never flatten on pressing and drying). Most of the species treated here (e.g., *L.biflora*, *L.laevis*, *L.parasitica*, *L.schizocalyx*) have translucent shiny pericarp (epicarp) at maturity (Fig. 5B, C, D), while others are opaque and matte (e.g., *L.banahaensis*, *L.oliveriana*; see Fig. 5A). Most species treated here have red (e.g., *L.biflora*, *L.laevis*), or orange-red berries (e.g., *L.banahaensis*, *L.parasitica*). Mature berries of *L.oliveriana*, however, are purple or purplish black.

Across the Solanaceae small inclusions known as sclerids, brachyclerids or stone cells are found in the fruit, often mixed amongst the seeds (Bitter 1911, 1914). In the large genus *Solanum*, this character is found in particular clades (e.g., Archaesolanum, Morelloid; see Symon 1994; Särkinen et al. 2018). These concretions are composed of modified sclerenchyma cells with massively enlarged cell walls; the stone cells of pears and quinces (Rosaceae) are classic examples of this cell type. Neither their function nor their origin in Solanaceae is known. Bitter (1914) suggested that they existed in an evolutionary series in the family, with more “advanced” taxa lacking them altogether. In the species treated here stone cells are found only in *L.rantonnetii* (Knapp 2022: 95, fig. 43), part of the clade that includes the type of *Lycianthes* (Särkinen et al. 2013). None of the species of *Lycianthes* native to Asia have been recorded as possessing stone cells (Knapp 2022).

### ﻿Seeds

Seeds of *Lycianthes* are usually flattened or compressed and are a variety of shapes across the genus (Dean et al. 2020), from round to ovoid. In the species treated here, seeds are flattened, often somewhat triangular in outline (e.g., *L.biflora*) and usually pale straw-coloured or pale brown. Seed number per berry varies from 2 (e.g., *L.parasitica*; Fig. 5E) to more than 100 seeds per berry (e.g., *L.biflora*); most species have in the range of 20–80 seeds per berry.

Seed coat morphology has been suggested as a useful character in the taxonomy of Solanaceae (Souèges 1907; Lester and Durrands 1984). The lateral walls of the outer seed coat epidermal layer can develop lignified radial thickenings that appear as hair-like structures (Souèges 1907; Lester and Durrands 1984; Peralta et al. 2008). These are present in several of the species treated here (e.g., *L.bimensis*, *L.shunningensis*) and can be useful in distinguishing some species (e.g., *L.biflora* from *L.shunningensis*). When the outer wall of the epidermis is removed, either naturally or by enzymatic digestion (Lester and Durrands 1984; Knapp 2002) such seeds appear pubescent; seed measurements here include these projections. The outline of seed testal cells varies from rectangular or pentagonal to deeply sinuate (also termed cerebelloid); this is usually constant within a species and can be useful confirming identifications.

### ﻿Chromosomes

Few chromosome counts have been done for native Asian *Lycianthes*. A specimen (*Symon 10652*) of *L.biflora* collected in New Guinea is tetraploid with n = 24 (Symon 1985), while Madhavadian (1968) cited a count of n = 12 from South India. This latter count is an unvouchered reference to a plant identified as *Solanumdenticulatum* Blume (= *L.biflora*) but given the locality “Ootacamund and Kodaikanal” I suspect this is a plant of *L.laevis*. Plants from this region have often been identified as *S.denticulatum*, but *L.biflora* does not occur in southern India. Vouchered chromosome counts for *Lycianthes* outside the Americas are a priority.

## ﻿﻿Ecology and natural history

### ﻿Distribution and habitats

Most of the species treated here are widely distributed throughout tropical Asia (see Fig. 1, Table 2) but are not found in Australia (except for *L.biflora* from Christmas Island). *Lycianthesbiflora* is the most widely distributed, occurring from India to southern China and Japan, including most of Indonesia, but *L.laevis* and *L.schizocalyx* are also species with wide distributions in the area. *Lycianthesbimensis* only found in the Lesser Sunda Islands and has the narrowest distribution of the species treated here. *Lycianthesoliveriana* is widely distributed on New Guinea but only found in the Malaku islands in the area treated here (see individual species treatments for distribution maps).

**Table 2.** Geographic distribution of *Lycianthes* in Asia by country (for Australia, New Guinea and the Pacific see Knapp 2022).

Country	Species
Bangladesh	*L.biflora*, *L.laevis*
Bhutan	*L.biflora*, *L.laevis*
Brunei Darussalam	*L.biflora*, *L.laevis*, *L.parasitica*
Cambodia	*L.biflora*
China	*L.biflora*, *L.laevis*, *L.lysimachioides*, *L.shunningensis*
India	*L.biflora*, *L.laevis*, *L.lysimachioides*, *L.rantonnetii*, *L.schizocalyx*, *L.shunningensis*
Indonesia	*L.banahaensis*, *L.biflora*, *L.bimensis*, *L.laevis*, *L.lysimachioides*, *L.oliveriana*, *L.parasitica*, *L.schizocalyx*
Japan	*L.biflora*, *L.laevis*, *L.lysimachioides*
Laos	*L.biflora*, *L.laevis*, *L.lysimachioides*, *L.shunningensis*
Malaysia	*L.biflora*, *L.laevis*, *L.lysimachioides*, *L.parasitica*, *L.schizocalyx*
Myanmar	*L.biflora*, *L.laevis*, *L.lysimachioides*, *L.schizocalyx*, *L.shunningensis*
Nepal	*L.biflora*, *L.laevis*, *L.lysimachioides*
Pakistan	*L.rantonnetii*
Philippines	*L.banahaensis*, *L.biflora*, *L.laevis*, *L.parasitica*, *L.schizocalyx*
Singapore	*Biflora*
Sri Lanka	*Laevis*
Taiwan	*L.biflora*, *L.lysimachioides*
Thailand	*L.biflora*, *L.laevis*, *L.lysimachioides*, *L.parasitica*, *L.schizocalyx*
Timor Leste	*L.biflora*
Vietnam	*L.biflora*, *L.laevis*, *L.lysimachioides*, *L.schizocalyx*, *L.shunningensis*

These species of *Lycianthes* are mostly plants of forests, growing either in the understory or at forest edges. *Lycianthesbiflora* is a weedy shrub occurring mostly at forest edges, in clearings and along roads and field edges in a wide variety of forest types, as are *L.laevis* and *L.schizocalyx*. *Lyciantheslysimachioides* is usually described as growing in deep shade or clambering over rocks in wet shady places near streams or waterfalls. *Lycianthesparastica* grows in lowland wet forests, sometimes in areas of peatland. Both *L.banahaensis* and *L.bimensis* are small trees of the forest understory.

### ﻿Reproductive systems

Most species of *Lycianthes* occurring in the Americas have both staminate and pistillate function operational in the same flower (see Dean et al. 2020). In the descriptions I have referred to these flowers as cosexual rather than hermaphroditic, following the terminology suggested by Cardoso et al. (2018) for describing the complexity of plant reproductive systems. While some species have been documented as self-incompatible (SI; e.g., Dean 2004) and others like *L.rantonnetii*, are likely to be SI based on lack of fruit set in cultivation, most species of *Lycianthes* have not been assessed for compatibility.

In contrast to most American species of *Lycianthes*, a few of the species in Asia (e.g., *L.banahaensis*, *L.bimensis*) and many of those from New Guinea (Knapp 2022) are clearly heterostylous and from specimens appear to have long-styled and short-styled flowers on different plants, suggesting they are androdioecious or dioecious (but see below for the possibility of leaky dioecy). Separation of function to different plants in these *Lycianthes* species has only been documented in the Pacific *L.vitiensis*, where both Reinecke (1898) and Smith (1991) suggested the species was androdioecious, with staminate (male) and cosexual (hermaphroditic) individuals. Symon (1985) suggested that many of the New Guinea species were probably dioecious but was not able to confirm this in the field. In *Solanum*, species exhibiting this set of traits were first described as androdioecious (see Symon 1970, 1979), a breeding system in which individual plants have either only staminate or only cosexual flowers. Further study, however, revealed that the putatively cosexual flowers had inaperturate pollen that functioned not in fertilisation but as a pollinator reward in these buzz-pollinated plants where pollen is the only reward (see Anderson 1979; Anderson and Symon 1989; Knapp et al. 1998; Martine et al. 2009; Anderson et al. 2015). All species of *Solanum* previously described as androdioecious have been shown to be dioecious, sometimes cryptically so (Anderson et al. 2015) with the floral morphology of staminate and pistillate flowers not markedly different. Staminate and pistillate plants of these cryptically dioecious species (e.g., *S.appendiculatum* Dunal, see Anderson 1979) were often described as different species, based on small differences in flower size. Dioecy has evolved in several *Solanum* lineages, and in some cases appears to be associated with islands (Anderson et al. 2015).

The putatively dioecious species of *Lycianthes* from Asia are like the cryptically dioecious species of *Solanum* in having staminate (male function) and pistillate (female function) flowers of very similar morphology, differing only in style length and occasionally in flower size. Field confirmation of the breeding system of these *Lycianthes* species is lacking, however, but from specimen evidence it appears that dioecy is the common state on New Guinea (Knapp 2022); it may be that the putatively dioecious species from Asia are closely related to this New Guinea radiation. Many of the dioecious solanums exhibit “leaky” dioecy, where the ability of staminate flowers to occasionally set fruit, perhaps enhancing their ability as colonists, either on islands (Anderson et al. 2015) or in new habitats (McDonnell et al. 2019). That there is a concentration of dioecious species of *Lycianthes* in the world’s largest tropical island means that these species perhaps represent an island radiation of dioecious taxa, like that seen in the spiny solanums of northern Australia (see Martine et al. 2009; Anderson et al. 2015). Field observations and studies of these largely rare forest plants are a priority for establishing the extent, degree, and evolution of the dioecious sexual system in *Lycianthes*.

### ﻿Conservation status

Most of the Asian species are widespread and relatively well-collected, with the exception of the range-restricted *L.bimensis* of the Sunda Islands. Preliminary conservation assessments for all species except the cultivated *L.rantonnetii* are presented in Table 3 and detailed in individual species treatments. None of these species have been formally assessed for the IUCN Red List.

**Table 3.** Preliminary conservation assessments for *Lycianthes* occurring in Asia. For *L.biflora* and *L.oliveriana* the New Guinea distribution is included.

Species	EOO (km^2^)	AOO (km^2^)	Preliminary assessment (EOO/AOO)
*Lycianthesbanahaensis*	1,652,883	228	LC/EN
*Lycianthesbiflora*	26,990,176	1,684	LC/VU
*Lycianthesbimensis*	38,448	24	NT/EN
*Lyciantheslaevis*	15,152,928	800	LC/VU
*Lyciantheslysimachioides*	14,512,768	372	LC/EN
*Lycianthesoliveriana*	1,006,290	156	LC/EN
*Lycianthesparasitica*	5,936,777	284	LC/EN
*Lycianthesschizocalyx*	7,731,851	368	LC/EN
*Lycianthesshunningensis*	1,388,419	72	LC/EN

## ﻿﻿Species concepts

My goal for this revision has been to provide circumscriptions for the members of this morphologically variable group of species. Delimitation of species here follows what is known as the “morphological cluster” species concept (Mallet 1995; Knapp 2008): i.e., “assemblages of individuals with morphological features in common and separate from other such assemblages by correlated morphological discontinuities in a number of features” (Davis and Heywood 1963). Biological (Mayr 1982), phylogenetic (Cracraft 1989) and the host of other finely defined species concepts (see Mallet 1995) are almost impossible to apply in practice and are therefore of little utility in a practical sense (see Knapp 2008). It is important, however, to clearly state the criteria for the delimitation of species, rather than dogmatically follow particular ideological lines (see Luckow 1995; Davis 1997). My decisions relied on clear morphological discontinuities to define the easily distinguished species. The probable dioecious nature of many of these species has complicated species recognition; staminate and pistillate (or cosexual) plants can have flowers of slightly different sizes, and the lack of fruit in many collections makes them difficult to place. Specific characters used for recognition are detailed with each species description and in the key. I have tried to emphasise similarities between populations instead of differences, which so often reflect incomplete collecting or local variation. My approach has been relatively conservative, defining as distinct entities those population systems (sets of specimens) that differ in several morphological characteristics. To some extent I have followed Joseph Dalton Hooker’s (Hooker and Thomson 1855) admonition to focus on similarities rather than local differences. In widespread species like *L.biflora* and *L.laevis* variation exists in certain characters, but inspection of large number of specimens reveals no apparent natural breaks in variation but rather a mixing between highly morphologically variable populations. Here the pattern of variation is such that no reliable units can be consistently extracted, nor is geography a completely reliable predictor of character states. These taxa might be best characterised as “ochlospecies” (Cronk 1998), whose taxonomy needs further investigation at a population level. This variation is described, while understanding that others may wish to interpret it differently.

## ﻿﻿Materials and methods

This revision is based on examination of herbarium material from 1,920 collections and observations (3,349 specimens) in the following 85 herbaria (herbarium acronyms follow Index Herbariorum, found on-line at http://sweetgum.nybg.org/science/ih/): A, AAU, AD, AHUC, AK, B, BISH, BKF, BM, BO, BR, BRI, BSHC, C, CAL, CANB, CAS, CHR, CINC, CORD, CTES, DAV, DD, E, F, FI, FLAS, FUEL, G, G-DC, GH, GXMG, GZU, HBG, HITBC, HN, IAC, IBK, IBSC, K, K-W, KAG, KIRI, KUN, L, LAE, LE, M, MBK, MBM, MEL, MICH, MO, MPU, MW, NDG, NSW, NY, NZFRI, P, PBL, PE, PERTH, PH, PNH, SI, SING, SP, SZ, TCD, TI, U, UBC, UBD, UC, US, USM, W, WAG, WELT, WIS, WU, WUK. Digital images of collections were consulted to add duplicates and new records; I have only included citations of digital images of which I am certain of the identification. The on-line resources at the Naturalis Biodiversity Research Center (https://bioportal.naturalis.nl/; U, L and WAG), the Muséum national d’Histoire Naturelle (https://science.mnhn.fr/institution/mnhn/collection/p/item/search/form; P), the United States National Herbarium at the Smithsonian Institution (https://collections.nmnh.si.edu/search/botany/; US) and the Chinese Virtual Herbarium (https://www.cvh.ac.cn/index.php) have been used extensively. Images of live plants on Co’s Digital Flora of the Philippines (Pelser et al. 2011 and onwards) have been hugely helpful for showing characters of living plants and are listed (along with other observational records) in in Suppl. material 1 as ‘Observation’.

An index to all numbered collections seen for this revision is presented in the body of the monograph. All collection events for all species of *Lycianthes* occurring as native plants (excluding cultivated plants) outside of the America are provided as a searchable csv file in Suppl. material 1. A searchable csv files of specimens examined for this monograph with barcodes and accession numbers if available is included as Suppl. material 2. All these files are also provided on the NHM Data Portal (https://doi.org/10.5519/xuvrw79j).

Measurements were made from dried herbarium material, with supplemental information on colour and texture taken from specimen labels. Specimens with coordinates on the labels were mapped directly, while the rest were georeferenced using the available locality data, sometimes supplemented by available collecting itineraries (van Steenis-Kruseman 1985). Maps were constructed with points in the centres of degree squares in a 1° square grid. Conservation threat status was assessed following the IUCN Red List categories and criteria (IUCN 2020) using the on-line assessment tools in GeoCat (http://geocat.kew.org; see Bachmann et al. 2011). The Extent of Occurrence (EOO) measures the range of the species, while the Area of Occurrence (AOO) represents the number of collecting points within the range based on a default grid of 2 km^2^; the AOO is highly sensitive to collecting bias, a known issue with plants generally.

Michel-Félix Dunal and Georg Bitter both worked extensively on *Solanum* (including *Lycianthes*) taxonomy. In their working practice they would cite multiple herbaria when they had seen specimens (duplicates) from several herbaria, but they often only cite a single herbarium (see nomenclatural notes for each species). Therefore, in cases where these authors cite a single herbarium and only a single specimen is found in that herbarium I have treated that as a holotype. I have searched each citation and not assumed that a single specimen exists. This resulted in usually finding many non-cited duplicate specimens, therefore very few of these names have holotypes (e.g., McNeill 2014).

Many of the names for Asian *Lycianthes* coined by Georg Bitter (Bitter 1919) are based on type specimens that were destroyed in the bombing of the Berlin herbarium (B) in the 1940s (see Vorontsova and Knapp 2010). Neither A.D.E. Elmer nor E.D. Merrill cited herbaria for the species they described from the Philippines; it is likely they used duplicated sets of material before distribution for these descriptions. Unfortunately, the Philippine National Herbarium (PNH) was destroyed by bombing during the Second World War (C.J. Luna, pers. comm. 20 Nov 2023), thus all duplicates are syntypes. Duplicates of some of these have been found and lectotypes selected from amongst these, and where I have not found duplicates I have neotypified these names with widely distributed collections that are congruent with the original descriptions and are from as near to the same area as the destroyed type specimen as possible. In all cases where possible, I have given preference to specimens held in herbaria in countries where these taxa are native.

For several of the species treated here various authors stated “type” or “holotype” and cited a single herbarium, inadvertently lectotypifying these names (Prado et al. 2015). I have indicated the original citations in square brackets. Type specimens with sheet numbers are cited with the herbarium acronym followed by the sheet number (e.g., SD [acc. # 6543]); barcodes are written as a continuous string in the way they are read by barcode readers (e.g., G00104280, MO-1781232). For those herbaria (e.g., A, GH, NY, US) where the barcode consists of only a number, I cite only the number string. Where herbaria have both barcodes and accession numbers, I always cite the barcode first, followed by the accession number (e.g., MO-503846, acc. # 3783069); this citation will allow users to access individual sheets where barcode numbers are not human-readable.

Citation of literature follows BPH-2 (Bridson 2004) with alterations implemented in IPNI (International Plant Names Index, http://www.ipni.org) and Harvard University Index of Botanical Publications (http://kiki.huh.harvard.edu/databases/publication_index.html). I have used the square bracket convention for publications in which a species is described by one author in a publication edited or compiled by another such as the traditional “in” attributions such as Dunal in DC. for those taxa described by Dunal in Candolle’s “*Prodromus Systematis Naturalis Regni Vegetabilis*”. This work is cited here as Prodr. [A.P. de Candolle] and the names are thus attributed only to Dunal. For “ex” attributions I cite only the publishing author, as suggested in the Code (Turland et al. 2018). Standard forms of author names are according to IPNI (International Plant Names Index; http://www.ipni.org).

## ﻿﻿Taxonomic treatment

### ﻿ Lycianthes

Taxon classificationPlantae

(Dunal) Hassl., Annuaire Conserv. Jard. Bot. Geneve 20: 180. 1917 nom. cons.

885D66B2-516A-59AC-8B86-2AD16970CB1C

Otilix Raf., Medical Fl. 2: 87. 1830, nom. utique rej. Type. Lyciantheslycioides (L.) Hassl. (as Solanumlycioides L.)
Solanum subsect. Lycianthes Dunal, Prodr. [A.P. de Candolle] 31(1): 29. 1852. Type (designated by D’Arcy 1972, pg. 211). Lyciantheslycioides (L.) Hassl. (as Solanumlycioides L.)
Parascopolia Baill., Hist. Pl. 9: 338. 1888, nom rej. Type. Lycianthesacapulcensis (Baill.) D’Arcy (as Parascopoliaacapulcensis Baill.)

#### Type.

*Lyciantheslycioides* (L.) Hassl. (as *Solanumlycioides* L.)

#### Description.

Perennial herbs, shrubs, vines, lianas or trees, sometimes epiphytic. Stems terete or angled, glabrous or pubescent with simple (unbranched), forked, dendritic or stellate trichomes, these usually eglandular, but sometimes glandular. New growth usually with minute papillae, these sometimes glandular. Sympodial units unifoliate or difoliate, if difoliate the leaves geminate and often differing in both size and shape (anisophyllous). Leaves simple, entire, glabrous or pubescent with simple (unbranched), forked, dendritic or stellate trichomes, these usually eglandular, but sometimes glandular; petioles well-developed or not. Inflorescences axillary or adnate to the stems and caulescent (*L.kaernbachii* of New Guinea only), fasciculate or with a short rhachis; pedicels articulated at the base. Flowers 4–6-merous, usually 5-merous, but some species (e.g., *L.banahaensis* often 4-merous), cosexual or heterostylous, long- and short-styled flowers borne on the same or different plants (in Australia, New Guinea and the Pacific probably dioecious). Calyx with various numbers (usually multiples of five, but sometimes fewer) appendages protruding from the calyx tube below or just at the truncate rim, or appendages lacking; appendages small bumps to obovoid to linear or linear subulate in shape. Corolla rotate to deeply stellate, white to deep purple or yellow (*L.banahaensis*), often with the midvein of the lobes darker and the centre paler or yellow-green, interpetalar tissue present or absent, the lobes minute (rotate corollas) or long-triangular, spreading, cupped or reflexed at anthesis. Stamens equal or unequal due to anther and/or filament differences; anthers plumply ellipsoid and obovate to tapering at the tips, usually dehiscing by apical pores opening to longitudinal slits with age or not, these occasionally longitudinally dehiscent. Ovary conical, glabrous; style straight or curved, the stigma minutely capitate, clavate or strongly bifid with diverging lobes. Fruit a berry, globose to ellipsoid to ovoid, green, orange, red or purple, sometimes with stone cells in the mesocarp. Seeds few to many, usually flattened, often triangular in outline, sometimes winged (e.g., *L.moszkowskii* of New Guinea). Chromosome number: n = 12, 24 (few species have chromosome counts).

#### Distribution and ecology.

Species of *Lycianthes* are found in the Americas, Asia, Australia, New Guinea and the islands of the Pacific. Species richness is concentrated in Mexico and Central America. No *Lycianthes* species are native to Africa, Europe or North America north of Mexico.

#### Discussion.

By far the greatest species diversity in *Lycianthes* occurs in the Americas (see Dean et al. 2020), but significant diversity occurs in Asia and New Guinea (Knapp 2022; see Fig. 1). The description above attempts to cover variation across the distribution, both in the Americas and outside.

As discussed above and in the Introduction, the species treated here are mostly found throughout Asia, and although they may not be a phylogenetically distinct group, they are geographically logical to treat as a unit.

##### ﻿﻿Artificial key to the species of *Lycianthes* in tropical Asia

**Table:** 

1	Corolla rotate; anthers yellow-orange, ellipsoid, slightly curved; berry yellow-green ellipsoid, with copious stone cells; stems angled and striped; cultivated plants	***Lycianthesrantonnetii***
–	Corolla stellate; anthers yellow or purple, ellipsoid or somewhat tapering, straight, often slightly beaked; berry variously coloured, without stone cells; stems terete, not angled and striped; native plants	**2**
2	Calyx appendages more than 5 (usually 10)	**3**
–	Calyx appendages 5 or fewer, or absent	**7**
3	Pubescence of mature leaves and stems of mixed simple uniseriate and branched (dendritic or forked) trichomes	***Lycianthesbiflora***
–	Pubescence of mature leaves and stems of simple uniseriate trichomes only or the plants glabrous	**4**
4	Herbs or at most sprawling subshrubs only woody at the base, rooting at the nodes; flowers 1(2) per fascicle; leaves of a geminate pair similar in shape often the same size	***Lyciantheslysimachioides***
–	Shrubs, the distal stems herbaceous but plants clearly woody at the base, not rooting at the nodes; flowers usually more than 2 per fascicle; leaves of a geminate pair different in size and shape	**5**
5	Calyx appendages of different lengths in the same flower; pedicels at anthesis (1)2–3 cm long; anthers 4–5 mm long	***Lycianthesschizocalyx***
–	Calyx appendages all the same length in the same flower; pedicels at anthesis 0.8–1.1 cm long; anthers 2–4.5 mm long	**6**
6	Leaves pubescent on both surfaces or glabrous, the bases attenuate or abruptly attenuate; calyx appendages arising from the rim of the calyx tube in flower, erect and spreading in fruit; anthers 2.5–4.5 mm long; seeds lacking “hairy” testal cell walls	***Lycianthesbiflora***
–	Leaves pubescent only on the adaxial surface (occasionally with a few trichome along the midrib abaxially), the bases acute or only somewhat attenuate onto the petiole; calyx appendages arising from below the calyx rim with a hyaline portion above their emergence; anthers 2–2.5 mm long; seeds with prominent “hairy” testal cell walls	***Lycianthesshunningensis***
7	Epiphytic shrubs; bark of mature stems pale, shiny and exfoliating; berries with few (2–4) seeds	***Lycianthesparasitica***
–	Trees, shrubs or lianas; bark of mature stems not as above; berries with 10+ seeds	**8**
8	Shrubs; corolla lobes membranous; flowers cosexual; berries with thin, shiny and translucent pericarp, juicy	***Lyciantheslaevis***
–	Trees or lianas; corolla lobes thick and fleshy; flowers heterostylous; berries with thicker, matte and opaque pericarp, not markedly juicy	**9**
9	New growth glabrous or only minutely papillate; flower buds plumply ellipsoid; calyx appendages absent; anthers 2–2.5 mm long; berries purple, acorn-like; lianas	***Lycianthesoliveriana***
–	New growth variously pubescent, usually with floccose trichomes; flower buds long-ellipsoid or slightly tapering; calyx appendages usually present as small nubs (longer in bud); anthers 2.5–4 mm long; berries orange or red; trees or treelets	**10**
10	Principal veins on mature leaves 6–7 pairs, not closely spaced; leaf bases acute to somewhat attenuate; style of pistillate flowers held almost within the anther cone, the stigma irregularly lobed; flowers white or yellow; leaf pubescence in herbarium specimens usually reddish brown	***Lycianthesbanahaensis***
–	Principal veins on mature leaves 10–14 pairs, closely spaced; leaf bases truncate; style of pistillate flowers exserted from the anther cone, the stigma clavate; flowers white or purple; leaf pubescence on herbarium specimens usually golden or pale tan	***Lycianthesbimensis***

##### ﻿﻿Synoptic character list for *Lycianthes* in Asia

This synoptical character list can be used as a multi-entry key for identification. I have only listed diagnostic characters here rather than the more common character state. For example, here I list inflorescences with more than 10 flowers, but not the more general case of few-flowered. For detailed distributional information please see Table 2. The list is intended to be used as a tool via a process of elimination; any character can be selected and in combination with other characters, a smaller selection of species can be obtained, for which the descriptions will be useful for coming to a final identification.

- Cultivated plants: rantonnetii
- Plants herbaceous and prostrate: lysimachioides
- Plants epiphytic shrubs: parasitica
- Plants woody lianas: oliveriana
- Plants small trees: banahaensis, bimensis
- Plants shrubby or sprawling shrubs: biflora, laevis, lysimachioides, rantonnetii, schizocalyx, shunningensis
- Pubescence on vegetative parts of dendritic (branched) trichomes: biflora
- Bark of stems pale, shiny and exfoliating: parasitica
- Bark of stems conspicuously lenticellate: banahaensis
- Calyx appendages absent, or apparently so: banahaensis, bimensis, laevis, oliveriana, parasitica
- Calyx appendages 5 or sometimes fewer: banahaensis, bimensis, laevis, parasitica
- Calyx appendages more than 5 (usually 10): biflora, lysimachioides, schizocalyx, shunningensis
- Calyx appendages markedly reflexed: shunningensis
- Calyx in fruit warty and partly enclosing the berry: oliveriana
- Flowers heterostylous: banahaensis, bimensis, oliveriana
- Corolla rotate: rantonnetii (cultivated)
- Corolla lobes somewhat fleshy and thick: banahaensis, bimensis, oliveriana
- Corolla yellow: banahaensis
- Anthers pubescent: biflora
- Berry bright red at maturity: banahaensis, bimensis, biflora, laevis, schizocalyx, shunningensis
- Berry purple or blackish purple at maturity: oliveriana
- Berry orange at maturity: parasitica
- Berry with copious stone cells: rantonnetii (cultivated)
- Berry with few seeds (2–4): parasitica
- Seeds with conspicuous “hairy” lateral cell walls: bimensis, parasitica, shunningensis

##### ﻿﻿Species descriptions

### ﻿﻿1. Lycianthes banahaensis

Taxon classificationPlantae

(Elmer) Bitter, Abh. Naturwiss. Vereins Bremen 24 [preprint]: 509. 1919.

FF9BC207-DD1E-5B41-84D3-42918FC7D192

**Figure 6.**
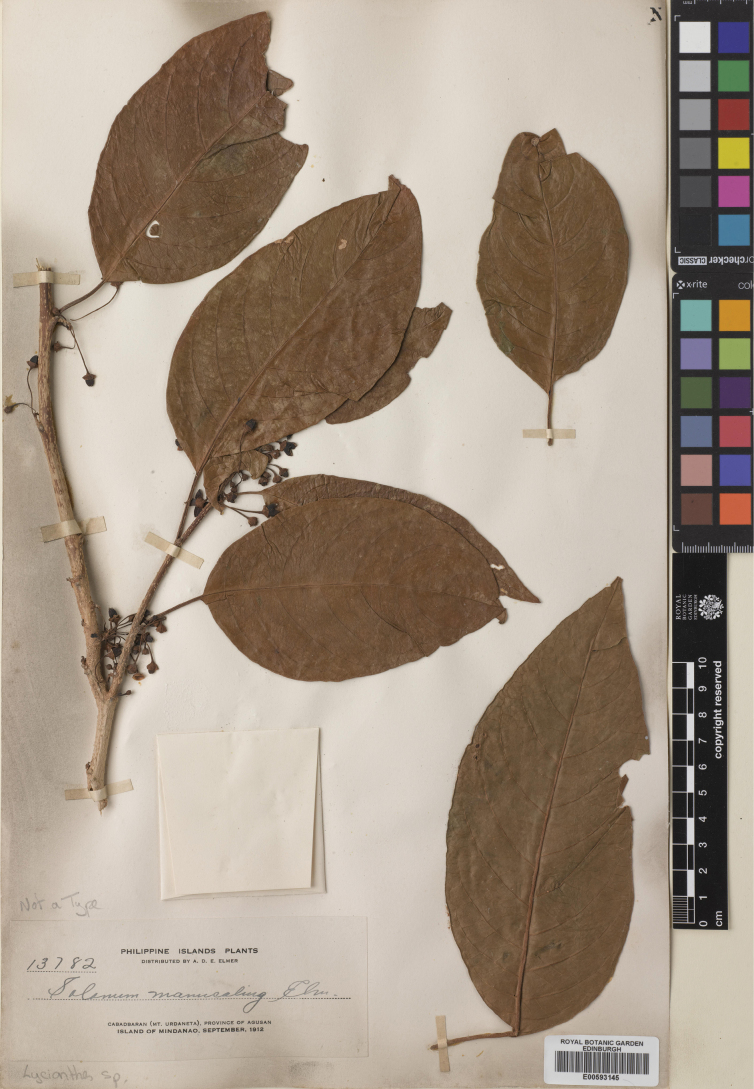
*Lycianthesbanahaensis* (Elmer) Bitter herbarium specimen. Philippines. Mindanao, *Elmer 13782* (E00593145, staminate specimen). Courtesy of Royal Botanic Garden, Edinburgh, reproduced with permission.

Figs 2A , 4A , 5A , 6

Solanum banahaense Elmer, Leaflets Philipp. Bot. 1: 341. 1908. Type. Philippines. Luzon [Calabarzon]: Tayabas, Lucban, May 1906, *A.D.E. Elmer 7492* (no herbaria cited; lectotype, designated here: A [00077843]; isolectotypes: CAL[CAL0000018718], E [E00273870], G [G00343322, G00415767], K [K000759394], L [L 0003585], LAE [acc. # 229579], LE, US [00027470, acc. # 629692], W [acc. # 1910-0010370]).
Solanum lagunense Elmer, Leaflets Philipp. Bot. 1: 341. 1908. Type. Philippines. Luzon [Calabarzon]: Los Baños, prov. of Laguna, Apr 1906, *A.D.E. Elmer 9425* (no herbaria cited; lectotype, designated here: K [K000759392]; isolectotype: E [E00426930], K [K000759393], LAE [acc. # 229581]).
Solanum manucaling Elmer, Leaflets Philipp. Bot. 2: 732. 1910. Type. Philippines. Mindanao [Davao]: Todaya (Mount Apo), Davao del Sur, May 1909, *A.D.E. Elmer 10489* (no herbaria cited; lectotype, designated here: BISH [BISH1005081, acc. # 581198]; isolectotypes: A [00077848], BM [BM000846477], CAL[acc. # 316482], E [E00273862], G [G00415768], GH [00077849], HBG [HBG-511353], K [K000759389], L [L 0003601], LE [LE00016976, LE00016977], MO [MO-716043, acc. # 3717134], NY [00172286], US [00027669, acc. # 873056], W [acc. # 1912-0001339]).
Solanum anisophyllum Elmer, Leaflets Philipp. Bot. 8: 2830. 1915. Type. Philippines. Mindanao [Caraga]: Cabadbaran, Mount Urdaneta [=Mount Hilong-Hilong or Masay], Agusan del Norte, Sep 1912, *A.D.E. Elmer 13887* (no herbaria cited; lectotype, designated here: A [00077840]; isolectotypes: BM [BM0001014585], CAL [acc. # 316453], E [E00196406], G [G00415766], GH [00077841], K [K000759392], L [L 0003597], LAE [acc. # 229574], LE [LE00016833], MICH [1109888], P [P00368935], U [U 0113983], US [00027453, acc. # 894985], W [acc. # 1915-0012862]).
Lycianthes aceratia Bitter, Abh. Naturwiss. Vereins Bremen 24 [preprint]: 508. 1919. Type. Indonesia. East Nusa Tenggara: Sumba [“Soemba, Parimbang” in protologue], *J.E. Teijsmann 8918* (holotype: BO [acc.# BO-1323491]; isotype: L [L.2859654]).
Lycianthes banahaensis (Elmer) Bitter subsp. manucaling (Elmer) Bitter, Abh. Naturwiss. Vereins Bremen 24 [preprint]: 509. 1919. Type. Based on Solanummanucaling Elmer
Lycianthes lagunensis (Elmer) Bitter, Abh. Naturwiss. Vereins Bremen 24 [preprint]: 511. 1919. Type. Based on Solanumlagunense Elmer
Lycianthes anisophylla (Elmer) Bitter, Abh. Naturwiss. Vereins Bremen 24 [preprint]: 512. 1919. Type. Based on Solanumanisophyllum Elmer
Lycianthes anisophylla (Elmer) Bitter var. masbateensis Bitter, Abh. Naturwiss. Vereins Bremen 24 [preprint]: 513. 1919. Type. Philippines. Luzon [Bicol]: Island of Masbate, Aug 1903, *E.D. Merrill 3046* (holotype: B [destroyed]; lectotype, here designated: US [0027867, acc. # 438017]; isolectotypes: BM [BM001019006], K [K000759391]).

#### Type.

Based on *Solanumbanahaense* Elmer

#### Description.

Trees or treelets, 3–10 m tall, to 15 cm diameter; stems terete, glabrescent, prominently white-lenticellate, the lenticels corky; new growth glabrous to densely pubescent with transparent, reddish yellow or brownish tan, simple, uniseriate 5–10-celled trichomes 0.2–0.75 mm long, the cells of trichomes small and beadlike (somewhat moniliform); bark of older stems glabrescent, pale greyish green or greyish white, prominently lenticellate. Sympodial units difoliate, the leaves geminate, the leaves of a pair differing in size and occasionally in shape. Leaves simple; blades of major leaves 5.5–30 cm long, 2.5–15 cm wide, elliptic, widest in the middle, discolorous, membranous or chartaceous, occasionally somewhat bullate; adaxial surfaces glabrous or with a few simple uniseriate trichomes like those of the new growth along the midrib, markedly shiny; abaxial surfaces glabrous or with scattered simple uniseriate trichomes on the midrib and major veins, not shiny; principal veins 6–7 pairs, glabrous or sparsely pubescent, drying darker abaxially; base acute and somewhat attenuate onto the petiole, or occasionally truncate (this only rarely and in very large leaves); margins entire; apex acute or acute with an attenuate acumen; petiole 0.6–5 cm long, glabrous or sparsely pubescent with simple uniseriate beadlike trichomes like those of the new growth; blades of minor leaves 3.5–9 cm long, 1.7–6 cm wide, elliptic to broadly elliptic or occasionally almost orbicular; surfaces like those of the major leaves; principal veins of minor leaves 4–5 pairs; base acute to truncate; margins entire; apex acute to somewhat attenuate; petiole of minor leaves 0.4–2 cm long, glabrous or sparsely pubescent. Inflorescences axillary, in fascicles or on a short rhachis 0.1–0.3 cm long, with 2–8(16) flowers, densely pubescent with simple uniseriate trichomes like those of the new growth and young stems; pedicels at anthesis 1.2–2 cm long, 0.5–0.75 mm in diameter at the base, 1.2–1.5 mm in diameter at the apex, spreading but held beneath the leaves, glabrous or more often sparsely pubescent with scattered simple uniseriate trichomes like those of the new growth and leaves, articulated at the base; pedicel scars tightly packed and overlapping on the short rhachis, somewhat corky. Buds ellipsoid, the corolla included in the calyx tube until just before anthesis, the calyx appendages prominent and often somewhat horn-like in bud. Flowers 4- or 5-merous, heterostylous and the plants probably dioecious, “with a peculiar smell” (fide *Adduru 109*). Calyx tube 2.5–3 mm long, 3–5 mm wide at the mouth, obconical, glabrous or sparsely pubescent with simple uniseriate trichomes like those of the pedicels, with 4–5 appendages arising 0.5–1 mm below the hyaline rim, the appendages 0.5–3.5 mm long, triangular to horn-like, usually perpendicular to the calyx tube, glabrous or with a few trichomes. Corolla 0.8–1 cm in diameter, white, cream (“milky”) or yellow, sometimes with a tinge of violet abaxially, stellate, lobed nearly to the base, abundant interpetalar tissue absent, the lobes 4–5 mm long, 2–4 mm wide, spreading, thick and fleshy, adaxially densely papillate, the midvein somewhat keeled in dry specimens, abaxially glabrous, the tips and margins densely papillate, the tips cucullate. Stamens equal and the same in short- and long-styled flowers; filament tube minute; free portion of the filaments less than 0.2 mm long, glabrous; anthers 2.5–4 mm long, 1.5–2.5 mm wide, plumply ellipsoid and somewhat beaked in some dry material, yellow, glabrous, poricidal at the tips, the pores tear-drop shaped and edged with white in dry material, lengthening to slits with age. Ovary conical, glabrous, vestigial in short-styled flowers; style in short-styled flowers absent, in long-styled flowers 2–3 mm long, held almost entirely within the anther cone, glabrous; stigma broadly clavate and irregularly lobed, bright dark green in live plants, the surfaces minutely papillate. Fruit a globose or occasionally somewhat ellipsoid berry, 1–2 cm in diameter, green when immature, orange to bright red when mature, the pericarp glabrous, thin, matte, and opaque; fruiting pedicels 2.5–4 cm long, 1–1.5 mm in diameter at the base, 2–3 mm in diameter at the apex, spreading, somewhat woody, green and prominently lenticellate; fruiting calyx not accrescent or expanding, a flattened disc below the berry. Seeds (5)20–60 per berry, ca. 3 mm long, ca. 2 mm wide, flattened and somewhat reniform, pale yellowish brown, the surfaces pitted, the testal cells sinuate in outline, the testal cell walls elongate but not prominently “hairy”. Stone cells absent. Chromosome number not known.

#### Distribution

**Figure 7.**
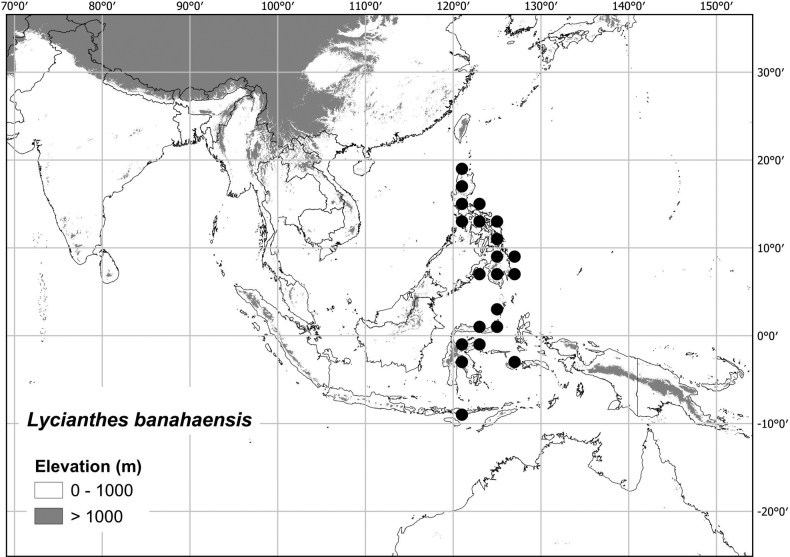
Distribution of *Lycianthesbanahaensis*.

**(Fig. 7).***Lycianthesbanahaensis* occurs in Indonesia and the Philippines; it is found on most islands of the Philippines and in eastern Indonesia from Sulawesi to the Sunda Islands, but not on Java or Borneo or in the Maluku Islands.

#### Ecology and habitat.

*Lycianthesbanahaensis* grows in low to middle elevation evergreen forests or forest edges, sometimes on limestone, from 50 to 1,500 m elevation.

#### Common names.

Indonesia. Sulawesi: onbeked (*Koorders 18050*), paratha kehu (Sangihe language, *Talangmin 51*), kaometi, makopi (Totemboan language, Koorders 1898, as “*S.minahassae*”).

#### Preliminary conservation assessment

**(IUCN 2020).**EOO (1,652,883 km^2^ - LC); AOO (228 km^2^ - EN). *Lycianthesbanahaensis* is known from more than five localities and is relatively widely distributed in the region. In the Philippines it is known from several protected areas (e.g., Mount Apo, Samar Island and the Northern Sierra Madre). The assessment of Endangered (EN) based on AOO is likely due to collecting bias, but also due to the island nature of the distribution. I therefore assign it a preliminary status of Least Concern (LC).

#### Discussion.

*Lycianthesbanahaensis* is a small tree (Fig. 2A) with shiny geminate leaves and prominently lenticellate stems and fruiting pedicels. The leaves are extremely variable in size; this has led to the description of the entity several times from different localities in the Philippines (see synonymy), as has variation in pubescence density on new growth. *Lycianthesanisophylla*, for example, was distinguished from *L.lagunense* in being “more glabrous” (Elmer 1915).

Plants appear to have either staminate flowers with vestigial ovaries or have berries, suggesting *L.banahaensis* is likely dioecious like many *Lycianthes* species from New Guinea (Knapp 2022). Flowers on fruiting specimens have short styles that are contained almost completely within the anther tube (see Fig. 4A); this means that recognising short- or long-styled flowers can be difficult, especially on dry specimens. Field verification of the reproductive biology of *L.banahaensis* is a priority (as is the case for most of these species outside the Americas).

Flowers of *L.banahaensis* are often tetramerous, and this character appears to be geographically fixed in populations, with many specimens from the Philippines mostly being 4-merous (Fig. 4A), while 5-merous collections are found in both the Philippines and Indonesia. The truly yellow flowers of many populations of *L.banahaensis* are unusual in *Lycianthes*, but cream or white flowers have been recorded on labels, so this appears not to be a constant character.

*Lycianthesbanahaensis* is most similar to *L.bimensis* of the Sunda Islands; both species are trees with fleshy, heterostylous flowers, dense fine pubescence on the new growth and orange or bright red mature berries. The prominently lenticellate stems and pale bark of *L.banahaensis* are diagnostic; *L.bimensis* has smooth stems and dark brown bark. *Lycianthesbanahaensis* has smaller flowers (corolla 0.8–1 cm in diameter versus 1.4–1.6 cm in diameter in *L.bimensis*) with the style of pistillate flowers held within the anther cone and strongly lobed (versus exserted and long-clavate in *L.bimensis*). Both species occur on the island of Flores (West Nusa Tenggara, Indonesia).

Adolph Elmer lived and collected in the Philippines from 1904 until his death in a Japanese internment camp in 1942. For the names coined by him (*S.banahaensis*, *S.lagunense*, *S.anisophyllum*), all specimens upon which they were based that were in his private collection at the Philippine National Herbarium (then the Philippine Bureau of Agriculture under United States jurisdiction) were destroyed by fire during the Japanese occupation of the Philippines in the Second World War just a day before the liberation of Manila (Schultes 1957). Duplicates of these are widely distributed, Elmer sold large numbers of specimens to Harvard, Geneva and Kew. For *Solanumbanahaense* I have chosen the duplicate held in the herbarium of the Arnold Arboretum at Harvard (A, barcode 00077843) as the lectotype; it is the best preserved of the single collection (*Elmer 7492*) cited in the protologue. Similarly, I have selected the more complete duplicate of *Elmer 9425* held at Kew (K000759392) as the lectotype for *S.lagunense*; other duplicates I have seen lack flowers or fruits. Elmer (1915) cited two collections in the protologue of *S.manucaling* (*Elmer 10489*, *Elmer 11693* – in flower and fruit respectively). I have selected the Bishop Museum duplicate of *Elmer 10489* (BISH1005081, acc. # 581198) as the lectotype, as it is one of the few duplicates of either of these collections that still has numerous flowers. For *S.anisophyllum*Elmer (1915) cited a single collection (*Elmer 13887*) now represented in many herbaria; most of the duplicates are sterile or only have flowers in packets. I have selected the Arnold Arboretum duplicate (A, barcode 00077840) as the lectotype as it has flowers attached to the stems.

Bitter (1919) cited a single collection in the Bogor herbarium (“Teysmann n. 8918! hb. Bogor”) in the protologue of *L.aceratia*. The Bogor specimen (acc.# BO-1323491) bears an annotation slip in Bitter’s distinctive hand, suggesting he had this material in his possession at some point. Bitter never travelled to Indonesia (Weber 1929) but must have had material on loan from Bogor itself; he never cites the Leiden collections in his *Lycianthes* monograph. This indicates he only saw the Bogor sheets of collections that are duplicated in Leiden, as when he examined sheets of the same collection from various herbaria his practice was to cite them all. I have therefore treated the Bogor sheet of *Teijsmann 8918* as the holotype of *L.aceratia*.

In the protologue of L.anisophyllavar.masbateensisBitter (1919) cited a duplicate of *Merrill 3046* at Berlin, now destroyed. I have selected the US duplicate (barcode 0027867, acc. # 438017) as the lectotype; Merrill worked for the United States government as the Director of the Philippine Bureau of Agriculture during the first quarter of the 20^th^ Century, leaving in 1923 to return to the United States (Schultes 1957). He regularly sent duplicates to the National Herbarium and the specimen there is the best preserved of those I have seen.

### ﻿﻿2. Lycianthes biflora

Taxon classificationPlantae

(Lour.) Bitter, Abh. Naturwiss. Vereins Bremen 24 [preprint]: 461. 1919.

23E2A03B-99C0-5BB6-B9FA-C1BF8737F2B0

**Figure 8.**
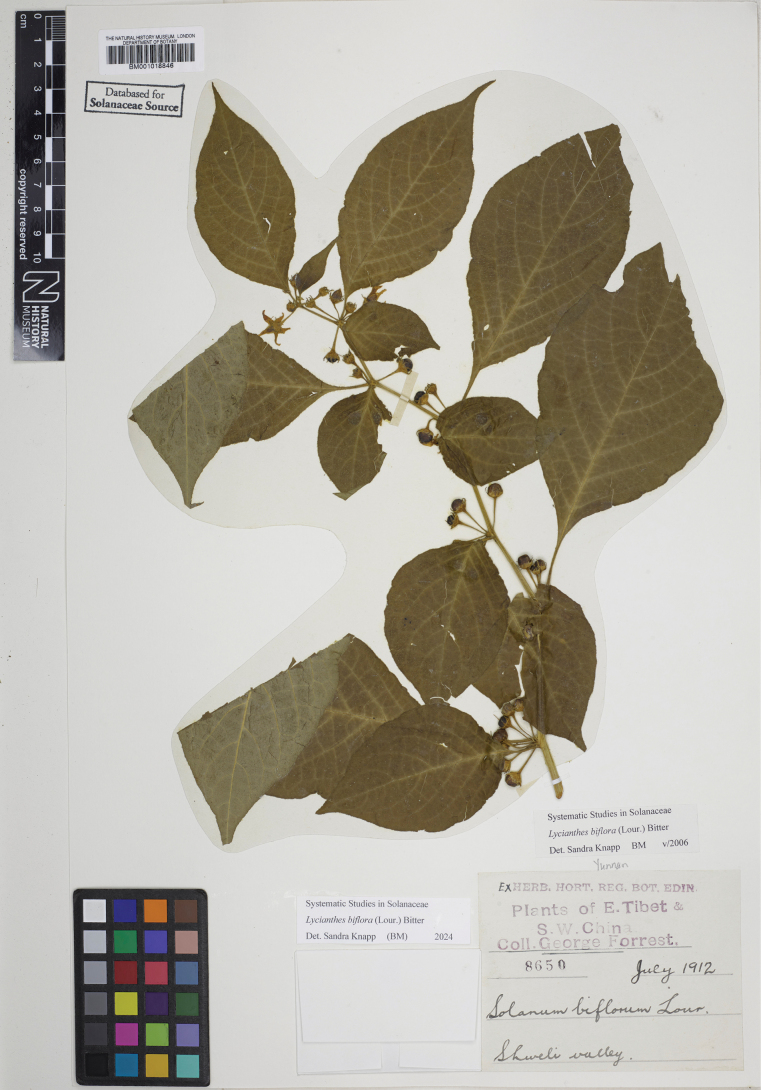
*Lycianthesbiflora* (Lour.) Bitter herbarium specimen. China. Yunnan, *Forrest 8650* (BM001018846). Courtesy of the Trustees of the Natural History Museum, London, reproduced with permission.

Figs 2B , 3A , 4B , 5B, C , 8

Solanum biflorum Lour., Fl. Cochinch. 129. 1790. Type. China. Guangdong: Guangzhou [“Pakwan supra Cantonem”], Jul 1869, *H.F. Hance 2128* (neotype, designated by Hul and Dy Phon 2014, pg. 57: P [P00058796]; isoneotypes: P [P00058797]).
Solanum decemdentatum Roxb. ex Wall., Fl. Ind. [ed. Carey & Wallich] 2: 247. 1824. Type. Singapore, Sep 1822, *N. Wallich cat. 2614 [A*] (lectotype, designated here: K-W [K001116583]; probable isolectotypes: BM [BM000900122], GZU [GZU000255527]).
Solanum denticulatum Blume, Bijdr. Fl. Ned. Ind. 13: 697. 1826. Type. Indonesia. Java [West Java]: Gagar Buiteng [in montosis Tjerimai et in cacuminae montis Gegar Bentin ex protologue], C.L. Blume s.n. (lectotype, designated here: L [L 0003595, L-bottom-most stem]).
Solanum mollissimum Blume, Bijdr. Fl. Ned. Ind. 13: 697. 1826. Type. No locality cited in protologue [Indonesia, Jav?], “in montanis”, C.L. Blume s.n. (lectotype, designated here: L [L 0003586]).
Solanum decemfidum Roxb. ex Nees, Trans. Linn. Soc. London 17(1): 43. 1834, nom. illeg. superfl. Type. Based on Solanumdecemdentatum Roxb. ex Wall. (cited in synonymy)
Solanum macrodon Wall. ex Nees, Trans. Linn. Soc. London 17(1): 43. 1834. Type. Bangladesh. [Chittagong: near Companiganj] [“Pundua, Wallich 2621, coll. Silva 1466”], F. de Silva s.n. [Wallich cat. 2621] (lectotype, designated by Deb 1978, pg. 293 [as “type collection”]: CAL [CAL0000017896]; isolectotypes: BM [BM001018882], E [E00273887], GZU [GZU000255675], K [K000923271], K-W [K001116626]).
Solanum zollingeri Dunal, Prodr. [A.P. de Candolle] 13(1): 176. 1852. Type. Indonesia. Java: [West Java], Tjidurian, Sep 1842, *H. Zollinger 723* (lectotype, designated here: G-DC [G00145615]; isolectotypes: BM [BM000778207], G [G00343041, G00357862], K [K000759388], P [P00369008]).
Solanum javanicum Dunal, Prodr. [A.P. de Candolle] 13(1): 176. 1852. Type. Indonesia. Java: sin. loc., *H. Zollinger 1981* (holotype: G-DC [G00145623]; isotypes: G [G00301655, G00343320], W [acc. # 1889-0149948, acc. # 1889-0149949]).
Solanum osbeckii Dunal, Prodr. [A.P. de Candolle] 13(1): 179. 1852. Type. Indonesia. Java: Prince’s Island, 4–14 Jan 1771, J. Banks & D. Solander s.n. (lectotype, designated here: BM [BM000778107]).
Solanum osbeckii Dunal var. stauntonii Dunal, Prodr. [A.P. de Candolle] 13(1): 179. 1852, as “Stauntoni”. Type. China. sin. loc., [s.d.], G. Staunton s.n. (lectotype, designated here: W [acc. # 0003086]).
Solanum calleryanum Dunal, Prodr. [A.P. de Candolle] 13(1): 178. 1852. Type. China. sin. loc. [protologue – “circa Macao”], *C. Gaudichaud 95* (lectotype, designated here: G-DC [G00145658]; isolectotype: G [G00415758]).
Solanum denticulatum Blume var. lanceolatum Miq., Fl. Ned. Ind. 2: 644. 1857. Type. Indonesia. Java: [protologue – “bij Lamadjang en Tenga”], H. Zollinger s.n. (no herbarium cited; lectotype, designated here: P [P00369015]; isolectotype: P [P00369014]).
Solanum biflorum Lour. var. mollissimum (Blume) Kuntze, Revis. Gen. Pl. 2: 453. 1891. Type. Based on Solanummollissimum Blume
Solanum biflorum Lour. forma pilosa Kuntze, Revis. Gen. Pl. 2: 453. 1891, as “Solanumbiflorumvar.mollisimumformapilosa” Type. Indonesia. Java: East Java, “Bromo 4,000” [=Mount Bromo], ca. 1220 m, *O. Kuntze 6007* (lectotype, designated here: NY [00172277]).
Lycianthes biflora (Lour.) Bitter var. sparsiloba Bitter, Abh. Naturwiss. Vereins Bremen 24 [preprint]: 464. 1919. Type. Indonesia. Java: “G. Tjibodas, Tjampen” [Cibodas], 200 m, *C.A. Backer 4658* (holotype: BO [acc. # BO-1911410]).
Lycianthes biflora (Lour.) Bitter var. mollissima (Blume) Bitter, Abh. Naturwiss. Vereins Bremen 24 [preprint]: 465. 1919. Type. Based on Solanummollissimum Blume
Lycianthes biflora (Lour.) Bitter var. grandifolia Bitter, Abh. Naturwiss. Vereins Bremen 24 [preprint]: 466. 1919. Type. Myanmar (Burma). “Papun bei Moulmein”, *A. Meebold 17068* (lectotype, designated here: CAL [acc. # 315674]; isolectotype: WRSL [destroyed]).
Lycianthes biflora (Lour.) Bitter var. subtusochracea Bitter, Abh. Naturwiss. Vereins Bremen 24 [preprint]: 466. 1919. Type. China. Yunnan: S of Red River, *A. Henry 13652* (holotype: B [destroyed]; lectotype, designated here: K [K000759409]).
Lycianthes biflora (Lour.) Bitter subsp. hupehensis Bitter, Abh. Naturwiss. Vereins Bremen 24 [preprint]: 466. 1919. Type. China. Hubei: sin. loc., “Faber in Henry’s Coll. from Centr. China” *A. Henry [Faber] 4304* (lectotype, designated here: W [acc. # 1886-0001758]; isolectotypes: B [destroyed], BM [BM001018849], CAL [acc. # 316326], E [E00426461], GH, K [K000759407], P [P00058798]).
Lycianthes biflora (Lour.) Bitter var. velutinella Bitter, Abh. Naturwiss. Vereins Bremen 24 [preprint]: 467. 1919. Type. Indonesia. Sulawesi: [Sulawesi Utara] “prov. Minahassa, Urwald bei Biwak Penamarangan bei Kajoevatoe [protologue]”, *S.H. Koorders 18041β* (no herbarium cited; lectotype, designated here: BO [acc.# BO-4324391]; isolectotype: L [L.2859422]).
Lycianthes biflora (Lour.) Bitter subsp. elongatidens Bitter, Abh. Naturwiss. Vereins Bremen 24 [preprint]: 468. 1919. Type. Indonesia. Sulawesi: [Sulawesi Utara] “Prov. Minahassa, Tomahon [Tomohon], 800 m”, *S.H. Koorders 18038β* (lectotype, designated here: BO [acc. # BO-1990064]; isolectotype: L [L.2859397]).
Lycianthes macrodon (Wall. ex Nees) Bitter, Abh. Naturwiss. Vereins Bremen 24 [preprint]: 468. 1919. Type. Based on Solanummacrodon Wall. ex Nees
Lycianthes macrodon (Wall. ex Nees) Bitter var. mollitersetosa Bitter, Abh. Naturwiss. Vereins Bremen 24 [preprint]: 470. 1919. Type. India. Sikkim: sin. loc., *T. Anderson 303* (lectotype, designated here: CAL [acc. # 315720]; isolectotype: B [destroyed]).
Lycianthes macrodon (Wall. ex Nees) Bitter var. sikkimensis Bitter, Abh. Naturwiss. Vereins Bremen 24 [preprint]: 470. 1919. Type. India. Sikkim: Toong, ca. 1,600 m, *A. Meebold 15768* (holotype: WRSL [destroyed], no duplicates found).
Lycianthes macrodon (Wall. ex Nees) Bitter var. manipurensis Bitter, Abh. Naturwiss. Vereins Bremen 24 [preprint]: 470. 1919. Type. India. Manipur: Ukrul Nagab, ca. 1,900 m, Dec 1907, *A. Meebold 6906* (holotype: WRSL [destroyed]; lectotype, designated here: CAL [no acc. #]).
Lycianthes denticulata (Blume) Bitter, Abh. Naturwiss. Vereins Bremen 24 [preprint]: 473. 1919. Type. Based on Solanumdenticulatum Blume
Lycianthes biflora (Lour.) Bitter subsp. yunnanensis Bitter, Repert. Spec. Nov. Regni Veg. 18: 319. 1922. Type. China. Yunnan: Mengtze, 1,570 m, *A. Henry 9160* (holotype: B [destroyed]; lectotype, designated here: K [K000922382]; isolectotypes: A [00619929], E [E00426448], US [02840689, acc. # 456785]).
Lycianthes macrodon (Wall. ex Nees) Bitter var. longifrons Bitter, Repert. Spec. Nov. Regni Veg. 18: 319. 1922. Type. India. Meghalaya: Birch Hill near Darjeeling, 2,200 m, 7 Nov 1896, H. Hallier s.n. (holotype: M [M-0166005]; isotype: CAL [no acc. #]).
Solanum boninensis Nakai ex Tuyama, Bot. Mag. (Tokyo) 50: 132, f. 27. 1936. Type. Japan. Ryuku Islands: Ins. Titizima [protologue, on label as “Bonin: Chichigima”], 8 Jul 1920, T. Nakai s.n. (holotype: TI [TI 00043095]).
Solanum biflorum Lour. var. glabrum Koidz. ex Hatus., J. Geobot. 17: 49. 1969. Type. Japan. Ryuku Islands: along the Kuira River, Isl. Iriomote, 6 Aug 1968, T. Narita s.n. (no herbarium cited in protologue; lectotype, designated here: KAG [acc. # 163736]; isolectotype: US [02840676, no accession number]).
Solanum biflorum Lour. var. kotoense Y.C.Liu & C.H.Ou, Quart. J. Chin. Forest. 7(4): 151. 1974, as ‘kotoensis’. Type. Taiwan. “Botel Tobago, low altitudes” [=Orchid Island], 7 Aug 1932, T. Sato s.n. (holotype: TAI [n.v.]).
Lycianthes biflora (Lour.) Bitter subsp. macrodon (Nees) Deb, Bot. J. Linn. Soc. 76: 293. 1978. Type. Based on Solanummacrodon Nees
Lycianthes hupehensis (Bitter) C.Y.Wu & S.C.Huang, Acta Phytotax. Sin. 16(2): 77. 1978. Type. Based on LycianthesbifloraLour.subsp.hupehensis Bitter
Lycianthes yunnanensis (Bitter) C.Y.Wu & S.C.Huang, Acta Phytotax. Sin. 16(2): 77. 1978. Type. Based on LycianthesbifloraLour.subsp.yunnanensis Bitter
Lycianthes laevis (Dunal) Bitter var. kotoensis (Y.C.Liu & C.H.Ou) T.Yamaz., Fl. Japan 193. 1993. Type. Based on SolanumbiflorumLour.var.kotoense Y.C.Liu & C.H.Ou
Solanum chingchunense S.S.Ying, New Taxa New Names 6(2): 311. 2023, as “*chingchunensis*”. Type. Taiwan. Hsinchu County: Wufeng Township, Chingchun, 550 m, 16 May 2023, S. S. Ying s.n. (holotype: NTUF [acc. # 112-073]).

#### Type.

Based on *Solanumbiflorum* Lour.

#### Description.

Small shrubs or herbs, 0.5–1.5 m tall, sometimes described as a vine or scrambler; stems terete, sparsely to densely pubescent with a mixture of transparent simple and/or forked or dendritic 3–10-celled uniseriate trichomes to 2 mm long, the dendritic trichomes antler-like or merely forked; new growth sparsely to densely pubescent with simple and dendritic trichomes like those of the stems, in plants with sparse pubescence the trichomes mostly confined to the leaf veins; bark of older stems pale brown, somewhat glabrescent. Sympodial units difoliate, the leaves geminate, the leaves of pair usually differing in size but usually not in shape. Leaves simple; blades of major leaves 3–17 cm long, 2–8 cm wide, ovate to elliptic or occasionally narrowly elliptic (e.g., *Chai 337360*) or broadly ovate (e.g., *Bodinier 799*), usually widest in the lower half but occasionally near the middle, somewhat discolorous or occasionally strikingly so (plants previously identified as var. subtusochracea), membranous, the leaves usually larger on lower branches; adaxial surfaces almost glabrous to evenly and moderately pubescent with transparent mixed simple and dendritic trichomes like those of the stems, these much denser along the veins; abaxial surfaces sparsely to moderately pubescent with the same trichomes as those of the adaxial surfaces, but the pubescence denser, markedly so on more distal leaves; principal veins 4–6 pairs, sparsely to densely pubescent, often drying yellow on both surfaces; base attenuate or occasionally abruptly attenuate, markedly decurrent onto the petiole; margins entire, markedly ciliate with transparent mixed simple and/or dendritic trichomes like those of the leaf surfaces; apex abruptly acuminate or acuminate; petiole 0.5–2.5 cm long, winged from the decurrent leaf bases, sparsely to densely pubescent like the stems and leaves; blades of minor leaves 1–5 cm long, 0.9–3 cm wide, shape, texture and pubescence like that of the majors; base attenuate onto the petiole; margins entire, ciliate; apex abruptly acuminate or acuminate; petiole 0.4–1(2.5) cm long, pubescent like the stems and leaves. Inflorescences axillary fascicles of (1)2–6 flowers, usually only one open at a time, sparsely to densely pubescent with mixed simple and dendritic trichomes like the stems; pedicels at anthesis 0.9–1.2 cm long, ca. 0.75 mm in diameter at the base, ca. 1.5 mm in diameter at the apex, nodding and the flowers borne below the leaves, sparsely to densely pubescent with transparent mixed simple and/or dendritic uniseriate like those of the stems and leaves, articulated at the base; pedicel scars tightly packed in the leaf axils. Buds elliptic, the corolla strongly exserted from the calyx tube before anthesis, the calyx appendages clasping the buds. Flowers 5-merous, apparently all cosexual. Calyx tube 2–3 mm long, 2.5–3.5 mm in diameter, obconical to openly cup-shaped, sparsely to densely pubescent like the stems and pedicels, with 10(12) linear awl-like appendages 1–9.5 mm long at anthesis, variable in length between plants and populations, the appendages emerging at the rim, pubescent like the rest of the calyx. Corolla 1.4–1.8 cm in diameter, white or lavender or purple, with a green central area, sometimes appearing as two green dots at the base of each lobe, stellate, lobed nearly to the base, interpetalar tissue absent or a thin edge on the lobes, the lobes 4–6 mm long, ca. 3 mm wide, spreading or slightly reflexed, membranous, adaxially glabrous, densely puberulent/papillate in the distal half abaxially, the tips and margins densely papillate. Stamens equal; filament tube minute; free portion of the filaments 0.5–1 mm long, glabrous; anthers 2.5–4.5 mm long, 1–1.5 mm wide, ellipsoid, the tips slightly pointed to a sharp beak to 0.5 mm long (the tips often drying paler than the rest of the anther), yellow, glabrous or variously pubescent with simple uniseriate trichomes to 0.2 mm long, these along the thecae edges or over the entire anther (e.g., *Cuong 417*), poricidal at the tips, the pores tear-drop shaped, distally directed, lengthening to slits with age. Ovary conical, glabrous; style 4.5–6 mm long, straight, glabrous; stigma small-capitate, the surfaces minutely papillate. Fruit a globose berry, 1–1.5 cm in diameter, bright red when ripe, changing from green to orange to red through development, the pericarp glabrous, thin, shiny and transparent; fruiting pedicels 1–1.8 cm long, ca. 1 mm in diameter at the base, ca. 1.5 mm in diameter at the apex, green, not markedly woody, erect with the fruits borne above the leaves; fruiting calyx a flat plate beneath the fruit, the calyx appendages elongating to ca. 2 times their length in flower, spreading and forming a star under the berry. Seeds 100+ per berry, 1.5–2 mm long, 1–1.5 mm wide, flattened and prismatically irregularly tear-drop shaped, straw-yellow, the surfaces deeply pitted, the testal cells sinuate in outline, prominent “hairy” testal cell walls absent. Stone cells absent. Chromosome number: n = 24 (Symon 1985; based on *Kairo & Symon 10652* from Papua New Guinea).

#### Distribution

**Figure 9.**
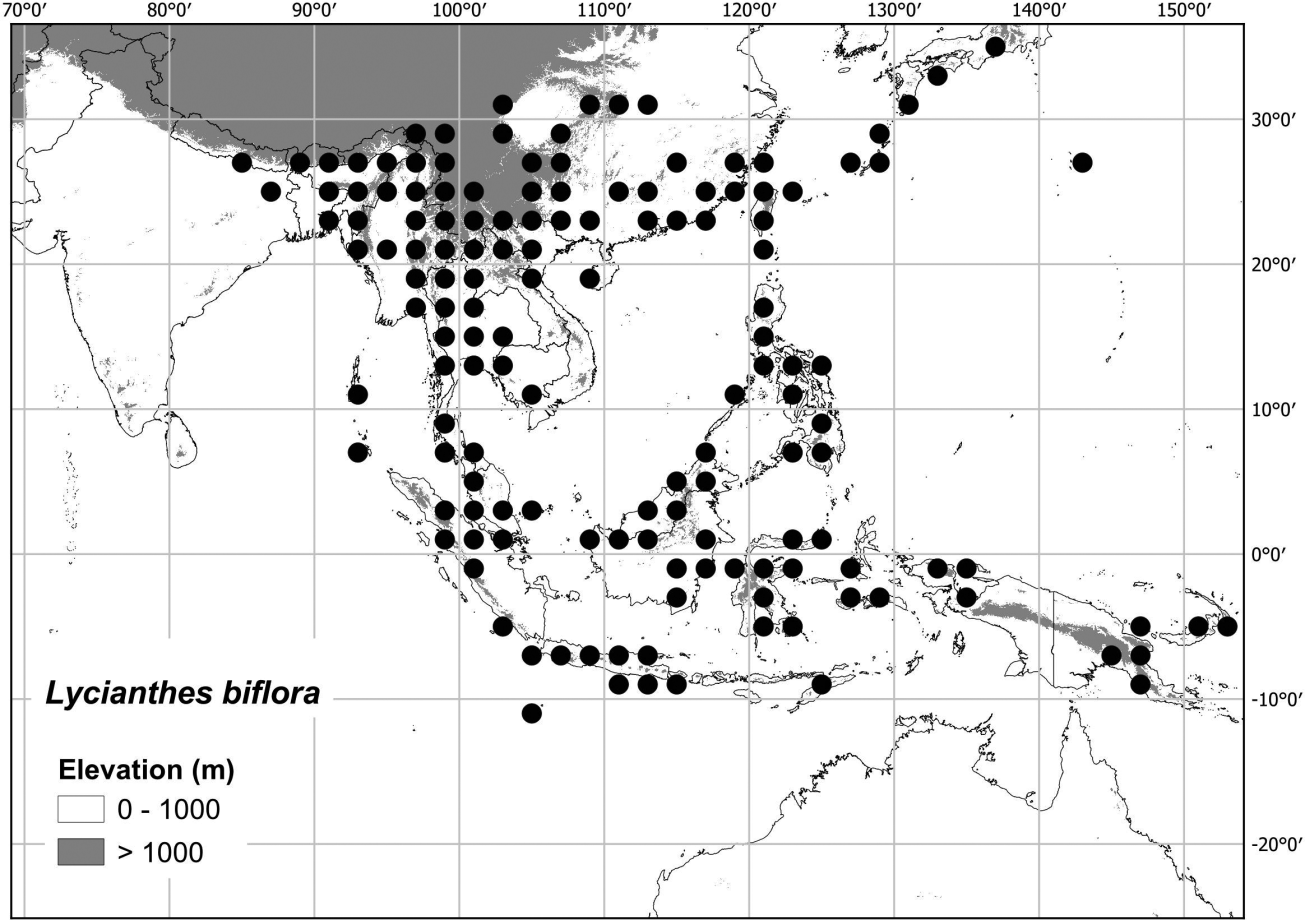
Distribution of *Lycianthesbiflora*.

**(Fig. 9).***Lycianthesbiflora* is widely distributed and very common; it occurs in Australia [Christmas Island only], Bangladesh, Bhutan, Brunei Darussalam, Cambodia, China, India, Indonesia, Japan, Laos, Malaysia, Myanmar (Burma), Nepal, Papua New Guinea, Philippines, Singapore, Taiwan, Thailand, Timor Leste and Vietnam.

#### Ecology and habitat.

*Lycianthesbiflora* grows in a wide variety of mostly disturbed habitats, in evergreen broadleaf or semi-evergreen broadleaf forests or in dry areas along roads and paths, from sea level to 2,300 m elevation. Most collections in the southern part of its range are from below 2,000 m elevation, but in the Himalaya it can grow at higher elevations.

#### Common names.

China. da chi hong si xian (as *L.macrodon*), dian hong si xian (as. *L.yunnanensis*), e hong si xian (as. *L.hupehensis*), hong si xian (Zhang et al. 1994); Guangdong: chicken eye clear, kai ngan ts’ing (*Lingnan University Herbarium 12648*); Hainan: shan nau kwo (*McClure 9600*). India. malum pavadam (*Wight 1569*); Sikkim: kolimbe (Lepcha language, *Srivastava 10301*). Indonesia. Sulawesi: lewa-lewsa-koelo, kamoeti (Totemboan language, Koorders 1898, as *S.denticulatum*), kalekamates (Tonsawant language, Koorders 1898, as. *S.denticulatum*), tahakkok, tohokok (Tombulu language, *Koorders 18308*), tampai (*Johansson 143*), wewelesan, wewelesan-in-taloen (Tombolu language, Koorders 1898, as *S.denticulatum*); Sumatra: akikaoe poga (*si Boeea 10780*), so haboe haboe (*si Boeea 10242*), si marpoga poga (*si Boeea 19071*), poga oetan (*si Boeea 10418*), poga (*si Boeea 6619*), poga poga (*si Boeea 8469*, *8765*, *9034*). Japan. Ryuku Islands: mejiro-hondzuki (*Amano 6961*). Malaysia. Sabah: lapak puru (Dusun language, *Bakia 612*), tensisiah (Dusun language, *Giking 236*), tutan (*Amin 93928*, Bundu Tutan language, *Aban Gibot 66986*), tutan geuton (Dusun language, *Surunda 100*), tutan puru (Dusun language, *Soibeh 789*); Sarawak: kerid pait (Kelabit language, *Christensen 71*), mata antu sebayan (Iban language, *Ashton 19162*), munting eja (*Yii 70379*). Nepal. banbi (*Mananduar 6805*). Philippines. Mindanao: beganbagan (Manobo language, *Elmer 13881*). Thailand. Central: ma wenj pa (*Put Phraisurind 156*). Vietnam. cà ngủ cuống to (as *L.macrodon*), cà ngủ (Hop 2017).

#### Preliminary conservation assessment

**(IUCN 2020).**EOO (26,990,176 km^2^ - LC); AOO (1,684 km^2^ - VU). *Lycianthesbiflora* is a weedy species known from more than five localities and is very widely distributed in the region. Throughout the range it is known from protected areas (e.g., Halimun National Park on Java, Lore Lindu National Park on Sulawesi, Indonesia; Iriomote National Park on Okinawa, Japan). The assessment of Vulnerable (VU) based on AOO is likely due to collecting bias, but also due to the island nature of the distribution. I therefore assign it a preliminary status of Least Concern (LC).

#### Discussion.

*Lycianthesbiflora* is a name that has been widely used for shrubby *Lycianthes* in tropical Asia. As discussed in the Introduction, Bitter (1919) characterised it as an “Overall species” (“Geasamart”) and split it into a multitude of infraspecific taxa, all of which are here recognised in synonymy. Most of these were distinguished based on geography or on pubescence variation, but examination of specimens from across the range show no real boundaries or diagnostic differences, something Bitter himself recognised was likely to occur with more material (Bitter 1919: 461).

*Lycianthesbiflora* is very similar to *L.schizocalyx*, which also has 10 linear to subulate calyx appendages (Fig. 3A, E). The appendages in *L.biflora* are all the same length in a single flower and arise from the edge of the calyx tube (Fig. 3A), leaving no hyaline rim above their emergence. The appendages of *L.schizocalyx*, in contrast are usually of variable length in a flower and at least some of them arise from below the edge of the tube, leaving a hyaline rim. The calyx appendages in *L.biflora* vary in length in individual plants and populations across the range (see Fig. 5B, C); plants from northern India with longer calyx appendages have been called *L.macrodon*. The flowering pedicels of *L.biflora* are 0.9–1.2 cm long, whereas those of *L.schizocalyx* are (1)2–3 cm long; individual specimens can sometimes be difficult to distinguish using this single character. It may also be that the species hybridise where they co-occur (i.e., places like Mount Kinabalu in Sabah, Malaysia). *Lycianthesshunningensis* is also a shrubby species with 10 calyx appendages, but these arise from well below the edge of the calyx tube and are distinctly turned downwards, especially in fruit (Fig. 5F). *Lycianthesbiflora* collections made from young plants can be difficult to distinguish from *L.lysimachioides*, but that species is a creeping or scandent herb, has a single flower per fascicle (only rarely more) and larger flowers (corolla 1.8–3 cm versus 1.4–1.8 cm in diameter in *L.biflora*).

Pubescence in *L.biflora* is remarkably variable; individual plants range from almost completely glabrous or with a few scattered simple uniseriate trichomes to densely pubescent. Densely pubescent individuals are found throughout the species range and have been called var. mollissima. Trichomes are either simple and uniseriate, often as many as 10 to 15 cells long, or variously branched. Branched trichomes in *L.biflora* usually bear only a few 1–2-celled branches but can be antler-like with many branches or merely forked (with a single branch). Density of pubescence is not a predictor of whether or not trichomes will be branched, and often a given plant will only have a few branched or forked trichomes with very short branches amidst denser simple uniseriate pubescence. Notations of glandular trichomes on some herbarium specimens are mistaken identification of fungal sporing bodies; I have seen no glandular trichomes in the specimens I have examined.

The anthers of some specimens of *L.biflora* are pubescent, with simple uniseriate trichomes along the dehiscence zone (e.g., *Forrest 24674* from Yunnan; *Chun 41918* from Hong Kong in China) or over the entire anther surface (e.g., *Cuong 417* from Vietnam). These hairs are reminiscent to those found in members of the Tomato clade of *Solanum* (Peralta et al. 2008), that hold the anthers together in a connivent structure that aids pollination. This would be an interesting species in which to investigate the genetic basis of the anther cone as has been done in *Solanum* (Glover et al. 2004).

In India, very pubescent individuals of *L.laevis* have often been annotated as *L.biflora*, but that species differs from *L.biflora* in its 5 calyx appendages that are either long and filiform or short and stubby. The name *L.denticulatum*, the type of which falls within my concept of *L.biflora*, was often applied to these southern Indian populations from the Courtallum area (Tamil Nadu).

The name *Solanumdecemdentatum* first occurred as a *nomen nudum* in Roxburgh’s Hortus Bengalensis (Roxburgh 1814) but was validated by Nathaniel Wallich in the edition of Flora Indica edited by him and William Carey (Carey and Wallich 1824). Here the name is attributed to Roxburgh, with citation of a plant seen by Wallich in Singapore and a reference to its similarity to *S.rumphii* Dunal (= *S.americanum* Mill., see Särkinen et al. 2018). A specimen in the Wallich herbarium at Kew (*Wallich cat. 2614*, K001116583) is labelled with both Singapore and a date preceding the description, matching the entry in Wallich’s distributed list of specimens (https://wallich.rbge.org.uk/index.php?section=entries&id=2614). I have selected this specimen as the lectotype of *S.decemdentatum*.

Blume (1826) recognised two varieties of his *S.denticulatum* as var. α and var. β, from different localities on Java. A sheet at Leiden (L 0003595) has three labels and three fragments of stem mounted together labelled “Solanumdenticulatum”. I have selected the lower L-most twig that seems to correspond to the adjacent label with the annotation “450/var. α /Gagar Bentang” as that bearing the most information linking it to the protologue. The other twigs mounted on the same sheet are likely to be syntypes; they are all labelled “Solanumdenticulatum” in a variety of different hands. An additional sheet (L 0003596) labelled “var. α” but does not have the same locality; it should also be considered a syntype. The description of *S.mollissimum* similarly does not cite a herbarium or collector (Blume 1826) nor is a locality named in the protologue. I have selected the sheet in Leiden from the Blume herbarium (L 0003586) labelled “Solanummollissimum Bl.” in Blume’s hand as the lectotype.

Dunal (1852) cited *Zollinger 723* from “v.s. in h. Boiss et DC” in the protologue of *S.zollingeri*. I have selected the better of these two specimens, that in G-DC (G00145615), as the lectotype for the species. The original collecting locality for this gathering, as is often the case for Zollonger’s collections, is on the specimen held in Paris. In describing *S.osbeckii*Dunal (1852) cited only “J.B. exs. in h. Banks”; the sheet at BM (BM000778107), collected by Joseph Banks and Daniel Solander is annotated in Dunal’s hand and is selected as the lectotype. In the protologue of S.osbeckiivar.stauntonii he cites a collection of “sir G. Staunton in h. Lambert”. The sheet in Vienna W (acc. # 0003086) is the only one I have seen that is unambiguously from Aylmer Bourke Lambert’s herbarium that was widely distributed by sale after his death (Miller 1970). Dunal (1852) described *S.calleryanum* from China, citing collections made by Joseph Callery housed in Paris and *Gaudichaud 95* from the De Candolle herbarium. Several un-numbered Callery collections are housed in Paris, all with different dates of collection. I have selected the sheet of *Gaudichaud 95* in G-DC (G00145658) as the lectotype, as it is well-preserved and unambiguously the specimen seen by Dunal.

Solanumdenticulatumvar.lanceolatum was described by Miquel (1864) with the citation of an un-numbered collection by H. Zollinger from “Lamadjang bei Tenga”. The only specimens I have seen with the correct locality and collector are in Paris and appear to be duplicates; one of these (P00369015) has an annotation in Miquel’s hand stating “Solanumdenticulatum Bl. var. lanceolt”. I select this sheet as the lectotype for the variety.

Kuntze’s herbarium is currently held at NY (Zanoni 1980); I have selected the sheet at NY (barcode 00172277) as the lectotype of S.biflorumformapilosa Kuntze, as it corresponds to the protologue and is annotated by Kuntze.

In his global monograph of *Lycianthes* Georg Bitter (1919) described many infraspecific taxa under both *L.biflora* and *L.macrodon*, here considered conspecific. Some of these cited a single specimen in a single herbarium (e.g., L.bifloravar.sparsiloba) and I have considered these holotypes. For those with single specimens cited from Berlin (where the Solanaceae were largely destroyed during World War II, see Vorontsova and Knapp 2010) or with multiple syntype collections cited, I have generally lectotypified these with specimens held in the countries where the plants are native, or from where they were described. For L.bifloravar.grandifoliaBitter (1919) cited two syntypes, *Meebold 17068* and *Meebold 17069*, both from WRSL; these specimens were destroyed in World War II and are no longer extant. A duplicate of *Meebold 17068* in CAL is designated the lectotype of this variety. Bitter (1919) cited a single specimen of *Henry 13652* from “herb. Berol.” (=Berlin) in the protologue of L.bifloravar.subtusochracea ; I have selected a sheet of this collection at Kew (K000759409) as the lectotype for var. subtusochracea. For L.biflorasubsp.hupehensisBitter (1919) cited two specimens of *Henry 4304* (as “Faber in Henry’s Coll. from Centr, China”), from “herb. Berol., Vindob.”. I have designated the syntype cited by Bitter from Vienna (W acc. # 1886-0001758) as the lectotype for this Chinese subspecies. No herbarium was mentioned for the only collection cited in the description of L.bifloravar.velutinella, *Koorders 18041β*. I have chosen the specimen in Bogor as the lectotype for var. velutinella (BO acc. # BO-4324391) as it is housed in Indonesia, where the type was collected. Bitter (1919) cited two collections in the protologue of L.biflorasubsp.elongatidens, *Koorders 18038β* from “herb. Bogor.) and *Sarasin 365* from Berlin. I have chosen the Bogor specimen of *Koorders 18038β* (acc. # BO-1990064) as the lectotype. Several collections were cited in the protologue of L.macrodonvar.mollitersetosa, *Anderson 303*, *1025* and *1026* all from Berlin and a collection from an un-named collector (“*Sammler 12011*”) from Bogor (Bitter 1919). The collection in Bogor (acc. # BO-1593104) has a label where the collector name is torn off the corner, but the handwriting and label style is unambiguously that of C.B. Clarke. Since this variety was described from Indian collections, I have selected the duplicate of *Anderson 303* held in Calcutta (CAL acc. # 315720) as the lectotype. Lycianthesmacrodonvar.sikkimensis was described (Bitter 1919) from *Meebold 15768* and cited as seen in WRSL. These collections are now destroyed, and I have found no duplicates. I have not designated a neotype in the hopes that a duplicate will be found. Lycianthesmacrodonvar.manipurensis was similarly described from a sheet in WRSL, *Meebold 6906*, but a duplicate of this gathering is held at CAL (without an accession number or barcode) and is here designated the lectotype.

Bitter later (Bitter 1922) described L.biflorasubsp.yunnanensis citing only *Henry 9160* from Berlin; I have selected the most complete of the several duplicates of this collection I have seen (with both flowers and fruits) as the lectotype (K000922382). In the same publication Bitter (1922) described L.macrodonvar.longifrons citing a Hallier collection in Munich that had on it two twigs. He differentiated the two as different varieties by stating (transl. from the original German by S. Knapp) “Next to the twig described above is a second fruiting twig, apparently collected there by Hallier, which has much richer and stronger hairs on the internodes, the fruit stalks and calyxes, and on both leaf surfaces; (Leaves considerably wider; lam. maj. 12:6.2 cm lam. min. 8:5 cm); this second specimen belongs to the var. mollitersetosa Bitter)” (Bitter 1922: 320). These two are likely to be duplicates.

The protologue of S.biflorumvar.glabrum (Hatushima 1969) has no herbarium cited, but Hatushima worked at Kagushima University and a specimen held there (KAG acc. # 163736) is a match for material held in US (barcode 02840676) that has attached a letter from S. Hatushima to E.H. Walker mentioning that this new variety would be described soon. I have selected the KAG specimen as the lectotype of this variety. Hatushima (1969) placed *S.schizocalyx* in synonymy with his new variety, based on the lack of pubescence mentioned in the protologue of *S.schizocalyx* (Merrill and Merritt 1910).

### ﻿﻿3. Lycianthes bimensis

Taxon classificationPlantae

(Miq.) Bitter, Abh. Naturwiss. Verein Bremen 24 [preprint]: 490. 1919.

D9E6791D-5413-5AC5-8DE2-3C4C9F0D3C76

Fig. 10

Solanum bimense Miq., Fl. Ned. Ind. 2: 642. 1857. Type. Indonesia. [West Nusa Tenggara]: Lesser Sunda Islands, Sumbawa, [“in Montosis Oö, Ins. Bima, +/- 2400” on BO label], ca. 730 m, 10 Oct 1847, *H. Zollinger 3458* (lectotype, designated here: BO [acc. # BO-1323395]; isolectotypes: BM [BM001019008], G [G00415764], GH, L [L.2881720], P [P00369045, P00369046, P00369047, P00369048], U [U 0113982]).

#### Type.

Based on *Solanumbimense* Miq.

#### Description.

Trees or treelets, 3–15 m tall, to 20 cm diameter; stems terete, glabrescent, sparsely pubescent with weak-walled transparent simple uniseriate 2–10-celled trichomes when young; new growth densely floccose pubescent with transparent, golden, simple, uniseriate 2–10-celled trichomes to 0.7 mm long, the trichomes moniliform and tangled; bark of older stems glabrescent, brown or greyish brown, smooth. Sympodial units difoliate, the leaves geminate, the leaves of a pair differing in size and occasionally in shape. Leaves simple; blades of major leaves 9–24 cm long, 4.5–14 cm wide, elliptic to elliptic-ovate, widest in the middle or just below, somewhat discolorous, membranous or chartaceous; adaxial surfaces glabrous to sparsely pubescent with simple uniseriate trichomes like those of the stems, these denser along the veins; abaxial surfaces glabrous to sparsely pubescent with simple uniseriate trichomes like those of the stems, if the lamina glabrous the trichomes confined to the principal veins and midrib; principal veins 10–14 pairs, glabrous or pubescent, the tertiary venation prominent, drying dark abaxially; base truncate and usually somewhat oblique; margins entire; apex acute to acuminate; petiole 2–7 cm long, glabrous or sparsely pubescent with simple uniseriate trichomes like those of the new growth; blades of minor leaves 3.5–9 cm long, 1.9–8 cm wide, ovate to orbicular; surfaces like those of the major leaves; principal veins of minor leaves 6–9 pairs; base truncate; margins entire; apex acute; petiole of minor leaves 0.7–2.5 cm long, glabrous or sparsely pubescent. Inflorescences axillary, in fascicles or on a short rhachis 0.2–0.5 cm long, with 8–16 flowers, glabrous or densely floccose pubescent with simple uniseriate trichomes like those of the new growth and young stems; pedicels at anthesis 1.4–1.5 cm long, ca. 0.5 mm in diameter at the base, ca. 1.5 mm in diameter at the apex, spreading, glabrous to densely floccose pubescent with simple uniseriate trichomes like those of the new growth and leaves, articulated at the base; pedicel scars tightly packed and overlapping on the short rhachis, somewhat corky. Buds long-ellipsoid, the corolla included in the calyx tube when young, ca. halfway exserted before anthesis, the calyx appendages more prominent in bud. Flowers 5-merous, heterostylous and the plants probably dioecious. Calyx tube 3–3.5 mm long, 3–4 mm wide at the mouth, obconical, glabrous to densely pubescent with simple uniseriate trichomes like those of the pedicels, with 5 appendages arising 0.1–0.5 mm below the hyaline rim, the appendages ca. 0.5 mm long, triangular to subulate, usually perpendicular to the calyx tube, glabrous or with a few trichomes. Corolla 1.4–1.6 cm in diameter, deep to pale violet, stellate, lobed nearly to the base, abundant interpetalar tissue absent, the lobes 7–8 mm long, 2–2.5 mm wide, spreading, thick and fleshy, glabrous on both surfaces, but with a few papillae on the slightly keeled midvein adaxially, the tips and margins densely papillate, the tips cucullate. Stamens equal and the same in short- and long-styled flowers; filament tube minute; free portion of the filaments ca. 0.5 mm long, glabrous; anthers 3.5–4 mm long, 1.2–1.5 mm wide, ellipsoid, yellow (?), glabrous, poricidal at the tips, the pores tear-drop shaped and edged with white in dry material, lengthening to slits with age. Ovary conical, glabrous, vestigial in short-styled flowers; style in short-styled flowers ca. 2.5 mm long, in long-styled flowers 8–10 mm long, exserted from the anther cone, glabrous; stigma ellipsoid to long-clavate, the tip sometimes 2-lobed, the surfaces minutely papillate. Fruit a globose berry, 0.8–1.2 cm in diameter, green when immature, bright red when mature, the pericarp glabrous, thin, matte, and opaque; fruiting pedicels 2–2.5 cm long, 1–1.5 mm in diameter at the base, 2.5–3 mm in diameter at the apex, spreading, somewhat woody and verrucose; fruiting calyx not accrescent or expanding, a flattened disc below the berry. Seeds 60–80+ per berry, 2.5–3 mm long, ca. 2.5 mm wide, rounded and not flattened, pale straw-colored, the surfaces deeply pitted, the testal cells pentagonal in outline and with prominent “hairy” lateral walls. Stone cells absent. Chromosome number not known.

**Figure 10.**
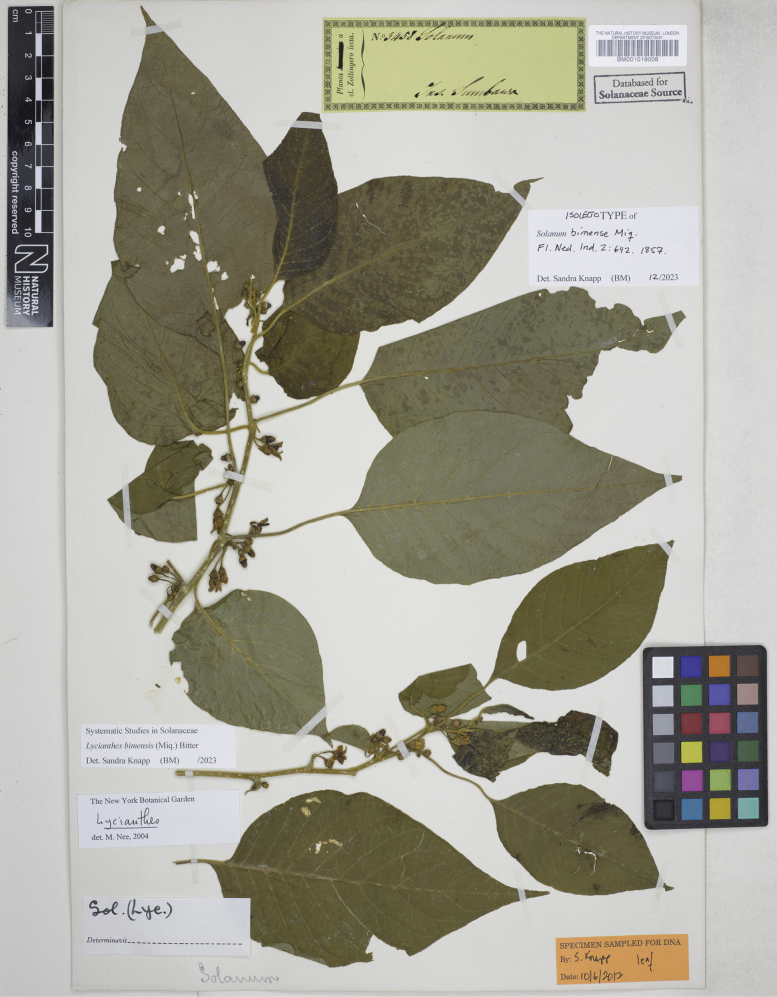
*Lycianthesbimensis* (Miq.) Bitter herbarium specimen. Indonesia. Sumbawa, *Zollinger 2057* (BM001019008; isolectotype of *S.bimense* Miq.). Courtesy of the Trustees of the Natural History Museum, London, reproduced with permission.

#### Distribution

**Figure 11.**
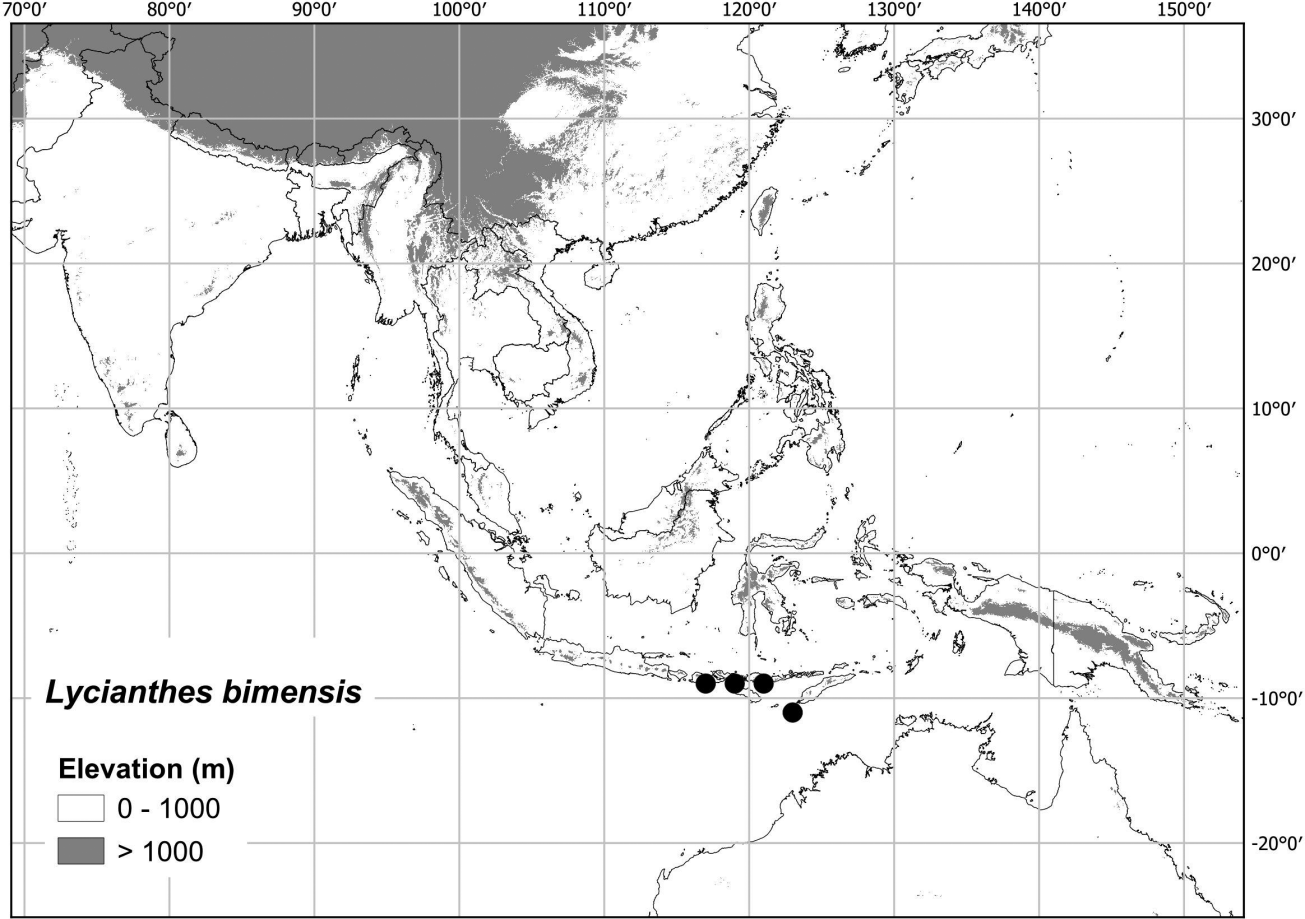
Distribution of *Lycianthesbimensis*.

**(Fig. 11).***Lycianthesbimensis* is confined to the lesser Sunda Islands of Flores, Sumbawa and Timor in the Indonesian provinces of East and West Nusa Tenggara.

#### Ecology and habitat.

*Lycianthesbimensis* is a forest treelet in moist to wet tropical forest on mountainsides, growing from 400 to 1,000 m elevation.

#### Common names.

None recorded.

#### Preliminary conservation assessment

**(IUCN 2020).**EOO (38,498 km^2^ - NT); AOO (24 km^2^ - EN). *Lycianthesbimensis* is a species known from only five localities and has a narrow distribution in the region. It occurs within the boundaries of Mount Tambora National Park on Sumbawa, but collections from there date to the 19^th^ century. The assessment of Vulnerable (EN) based on AOO is likely due to collecting bias, but also due to the island nature of the distribution. But based on the number of localities and threats to the habitat I consider *L.bimensis* to be of some conservation concern and therefore suggest a preliminary status of VU (B2a, b[iii]).

#### Discussion.

*Lycianthesbimensis* is a distinctive plant with long-petiolate leaves with prominent and crowded principal veins (Fig. 10). The flowers are heterostylous, and I have not seen berries or developing berries on specimens with short-styled flowers, suggesting *L.bimensis* is dioecious. The calyx of *L.bimensis* has 5 small appendages borne somewhat below a hyaline rim; it can be distinguished from other species with 5 calyx appendages such as *L.laevis* in its tree habit, fleshy corolla lobes (versus membranous lobes) and heterostylous flowers. The calyx tube in *L.bimensis* is usually larger (3–3.5 mm versus 1.5–2.5 mm) and more robust than that of *L.laevis*.

*Lycianthesbimensis* is most similar to *L.banahaensis*, both species are trees with fleshy, heterostylous flowers, dense fine pubescence on the new growth, 5 calyx appendages and orange or bright red mature berries. *Lycianthesbimensis* has smooth stems and dark brown bark versus the diagnostic prominently lenticellate stems and pale bark of *L.banahaensis*. Leaf petioles in *L.bimensis* are generally longer relative to leaf size than in *L.banahaensis*, and the flowers of *L.bimensis* are larger (corolla 1.4–1.6 cm in diameter versus 0.8–1 cm in diameter in *L.banahaensis*). The style of long-styled flowers of *L.bimensis* is exserted from the anther cone and long-clavate whereas that of *L.banahaensis* has the style of long-styled flowers contained within the anther cone and strongly lobed. Both species occur on the island of Flores (West Nusa Tenggara, Indonesia).

The type collection of *L.bimensis* is from an expedition to Mount Tambora on the island of Sumbawa and was made by the prolific Swiss collector Heinrich Zollinger, who studied in Geneva with the de Candolles and whose collections are widely distributed. The massive eruption of Tambora in 1815 is the largest historic volcanic eruption (Oppenheimer 2003), expelling more volume of rock and ash and with more fatalities than that of the more well-known Krakatoa. In his time collecting in Java and the surrounding area in the 1840s, Zollinger wished to study Tambora after the eruption to assess its effects on the local ecosystem and to document plant recovery (Zollinger 1855). In 1847 he and his companions were the first scientists to ascend the volcano after the eruption. The summit was still wreathed in smoke but he records vegetation recovery at various levels, and also reflects that he occasionally fell through a crust to land in a warm sulphurous powder. The type collection of *S.bimense* is not on Tambora itself, but on an adjacent peak (Doro Omomboha, on Zollinger’s map labelled as Buha) nearer to the town of Bima.

I have lectotypified *S.bimense* with the sheet of *Zollinger 3458* in the Bogor herbarium (BO acc. # BO-1323395) as no herbarium was mentioned in the protologue (Miquel 1864) and the specimen is housed in Indonesia, where *L.bimensis* is endemic. Miquel never travelled to Indonesia (Stafleu 1966); it is likely that he either saw material in Leiden, where he was director of the Rijksherbarium, or sets of duplicates readied for distribution.

The designation “*Solanumfloccosum* Zipp.” was published in a list of names with no description or diagnosis (Spanoghe 1841) rendering it a nomen nudum.

### ﻿﻿4. Lycianthes laevis

Taxon classificationPlantae

(Dunal) Bitter, Abh. Naturwiss. Verein Bremen 24 [preprint]: 484. 1919.

CB5CD75D-B3FE-5A21-A23D-EA351755BC48

Figs 3B , 4C , 12

Solanum laeve Dunal, Encycl. [J. Lamarck & al.] Suppl. 3: 751. 1814. Type. Indonesia. [Java]: Goudolone, *J.B.L. Leschenault de la Tour 683* (lectotype, designated here: P [P000578637]).
Solanum violaceum Blume, Cat. Gew. Buitenzorg 55. 1823, nom illeg. not Solanumviolaceum Ortega (1798). Type. Indonesia. “Java”, C.L. Blume s.n. (no specimens cited; neotype, designated here: L [L 0003600]).
Solanum crassipetalum Wall., Fl. Ind. (Carey & Wallich ed.) 2: 256. 1824. Type. Nepal. “Sheopar”, 1821, R. Blinkworth s.n. [Wallich cat. 2618] (lectotype, designated by Deb 1978, pg. 293 [as “type” and with collector R. Brown]: CAL [CAL0000017942]; isolectotypes: BM [BM000617159], E [E00273881, E00273882], G-DC [G00131653], K [K0009234440], K-W [K001116619, K001116620]).
Solanum blumei Nees ex Blume, Bijdr. Fl. Ned. Ind. 13: 696. 1826, as “*blumii*”. Type. Based on Solanumviolaceum Blume
Solanum pachypetalon Spreng., Syst. Veg. ed. 16 [Sprengel] 4 (2): 72. 1827, nom. illeg. superfl. Type. Nepal. Based on Solanumcrassipetalum Wall. (cited in synonymy).
Solanum membranaceum Wall. ex Nees, Trans. Linn. Soc. London 17(1): 41. 1834. Type. India. [probably southern India], *N. Wallich Cat. 2625A* (lectotype, designated here: K-W [K001116640]).
Solanum bigeminatum Nees, Trans. Linn. Soc. London 17(1): 42. 1834. Type. India. “Travancore”, Wallich cat. suppl., Oct 1814, Anon. s.n. [*Herbarium Rottlerianum*] (lectotype, designated by Hepper 1987, pg. 388: K [K000759406]).
Solanum neesianum Wall. ex Nees, Trans. Linn. Soc. London 17(1): 42. 1834. Type. India. [Khasi Hills] “Mt Sylhet”, N. Wallich [G. Gomez s.n.] Cat. Suppl. 248 (lectotype, designated here: GZU [GZU000255706].
Solanum zollingeri Dunal var. multiflorum Dunal, Prodr. [A.P. de Candolle] 13(1): 176. 1852. Type. Indonesia. Java: [no locality in protologue] East Java, in summo montis Taroeb [=Gunung Tarub], prov. Probolingo, 4 Jan 1845 [from P00369020], *H. Zollinger 2597* (holotype: G-DC [G00145616]; isotypes: BM [BM001018925], G [G0343077, G000357860], P [P00369013, P00379534 (as *2597 bis*), P [P00369020, as *2597Z*]).
Solanum subtruncatum Dunal, Prodr. [A. P. de Candolle] 13(1): 180. 1852. Type. Bangladesh. “In India orientalis, Silhet, Wall. cat. 2620”, F. de Silva s.n. [Wallich cat. 2620] (holotype: G-DC [G00145656]; isotypes: BM [BM000778231], CAL [CAL0000071895], GZU [GZU000255845], K [K001116625]).
Solanum kaitisis Dunal, Prodr. [A. P. de Candolle] 13(1): 157. 1852. Type. India. Tamil Nadu: Nilghiri, Kaithi [“ad montis Nilgherry circa Kaitis” – protologue], 1840, *G.S. Perrottet 270 [as 230*] (holotype: P [P00058859]).
Solanum gouakai Dunal, Prodr. [A. P. de Candolle] 13(1): 177. 1852, as “Gouakai”. Type. India. [Tamil Nadu]: “Nillgherry” [Nilgiri Hills], *J.B.L. Leschenault de la Tour 311* (lectotype, designated here: P [P00057927]).
Solanum gouakai Dunal var. angustifolium Dunal, Prodr. [A. P. de Candolle] 13(1): 177. 1852. Type. India. [Tamil Nadu]: “Nillgherry” [Nilgiri Hills], *J.B.L. Leschenault de la Tour 311* (lectotype, designated here: P [P00057928]).
Solanum gouakai Dunal var. latifolium Dunal, Prodr. [A. P. de Candolle] 13(1): 177. 1852. Type. India. [Tamil Nadu]: “Nillgherry” [Nilgiri Hills], *J.B.L. Leschenault de la Tour 311* (lectotype, designated here: P [P00057929]).
Bassovia wallichii Dunal, Prodr. [A. P. de Candolle] 13(1): 409. 1852, nom. illeg.superfl. Type. Based on S.crassipetalum Wall. (“Solanumcrassipetalum, Wall. cat. 2618”, see discussion).
Solanum blumei Nees var. parvifolium Miq., Fl. Ned. Ind. 2: 642. 1857, as “*parvifolia*”. Type. Indonesia. Sin. loc. “S. Eschweilerianum”, A. Zippelius s.n. (no herbaria cited; lectotype, designated here: L [L 0003598]; isolectotype: L [L 0003599]).
Solanum blumei Nees var. grandifolium Miq., Fl. Ned. Ind. 2: 642. 1857, as “grandifolia”. Type. Indonesia. Sin. loc., T. Horsfield s.n. [1421] (neotype, designated here: BM [BM001018973]).
Solanum bigeminatum Nees var. zeylanica C.B.Clarke, Fl. Brit. India [J. D. Hooker] 4: 231. 1883. Type. Sri Lanka. Sin. loc., [no date], *G. Gardner 628* (lectotype, designated here: K [K000923382]; isolectotypes: BM [BM000900109], K [K000923458], TCD [TCD51387]).
Solanum blumei Nees var. erythrocarpum Kuntze, Revis. Gen. Pl. 2: 453. 1891. Type. India. Sikkim: sin. loc., “5,000 ft”, 21 Nov 1875, *C.E.O. Kuntze 6841* (lectotype, designated here: NY [00172265]).
Solanum blumei Nees var. xanthocarpum Kuntze, Revis. Gen. Pl. 2: 453. 1891. Type. Indonesia. Java: West Java, “Wilisgebirge” [Mount Wilis], “5,000 ft”, 29 Aug 1875, *C.E.O. Kuntze 5855* (lectotype, designated here: NY [00172280]).
Solanum mindanaense Elmer, Leafl. Philipp. Bot. 8: 2832. 1915. Type. Philippines. Mindanao, prov. Agusan, Cabadbaran (Mt. Urdaneta), Sep 1912, *A.D.E. Elmer 13828* (syntype: E [E00273869]).
Lycianthes pachypetala (Spreng.) Bitter, Abh. Naturwiss. Verein Bremen 24 [preprint]: 475. 1919. Type. Based on Solanumpachypetalon Spreng.
Lycianthes pachypetala (Spreng.) Bitter var. intermedia Bitter, Abh. Naturwiss. Verein Bremen 24 [preprint]: 477. 1919. Type. India. Sikkim: Tashok [as Pashok in protologue] (=Tshoka), 1,594 [1,600 in protologue] m, *T. Anderson 1030* (lectotype, designated here: CAL [acc. # 316742]).
Lycianthes pachypetala (Spreng.) Bitter var. grandis Bitter, Abh. Naturwiss. Verein Bremen 24 [preprint]: 478. 1919. Type. India. “Sikkim”, Feb 1909, “Native collector” s.n. (holotype: WU [WU0155118]; isotype: W [acc. # 1909-0009669]).
Lycianthes subtruncata (Dunal) Bitter, Abh. Naturwiss. Verein Bremen 24 [preprint]: 478. 1919. Type. Based on Solanumsubtruncatum Dunal (as Solanumsubtruncatum Wall.)
Lycianthes subtruncata (Dunal) Bitter var. hypolasia Bitter, Abh. Naturwiss. Verein Bremen 24 [preprint]: 480. 1919. Type. India. “Assam”, Masters s.n. (“ex herb hort. Calcutta, hb. Bogor”; not found at BO, no duplicates found).
Lycianthes bigeminata (Nees) Bitter, Abh. Naturwiss. Verein Bremen 24 [preprint]: 480. 1919. Type. Based on Solanumbigeminatum Nees
Lycianthes bigeminata (Nees) Bitter subsp. nodocalyx Bitter, Abh. Naturwiss. Verein Bremen 24 [preprint]: 481. 1919. Type. India. Karnataka: “In campis aridis prope Mercara”, *R.F. Hohenacker 803* (lectotype, first stage designated by Hepper 1987, pg. 390 [as “type”], second stage designated here: K [K000759401]; isolectotypes: BM [BM000900101], FI [FI009598], M [M00165986], SLO [n.v.]).
Lycianthes bigeminata (Nees) Bitter subsp. kaitisis (Dunal) Bitter, Abh. Naturwiss. Verein Bremen 24 [preprint]: 481. 1919, as ‘Kaitisis’. Type. Based on Solanumkaitisis Dunal
Lycianthes bigeminata (Nees) Bitter var. parvifrons Bitter, Abh. Naturwiss. Verein Bremen 24 [preprint]: 482. 1919. Type. India. Tamil Nadu: Nilghiri Hills, Ooty [“Ootacamund, Nilgiri Hill, prov. Madras. 7,000 ft”], Jun 1882, *D. Brandis 350* (lectotype, designated here: HBG [HBG-511357]).
Lycianthes bigeminata (Nees) Bitter var. calycodonta Bitter, Abh. Naturwiss. Verein Bremen 24 [preprint]: 482. 1919. Type. India. Karnataka: Kulhutty, “Kulhutty, Bababood”, ca. 1,900 m, Oct 1908, *A. Meebold 8878* (lectotype, designated here: CAL [acc. # 315662]).
Lycianthes bigeminata (Nees) Bitter forma gouakai (Dunal) Bitter, Abh. Naturwiss. Verein Bremen 24 [preprint]: 483. 1919, as “Gouakai”. Type. Based on Solanumgouakai Dunal
Lycianthes laevis (Dunal) Bitter var. brevipedicellata Bitter, Abh. Naturwiss. Verein Bremen 24 [preprint]: 487. 1919. Type. Indonesia. “Sumatra? Poeding zimbo! hb. Bogor” (no specimens found at BO).
Lycianthes laevis (Dunal) Bitter var. inaequidens Bitter, Abh. Naturwiss. Vereins Bremen 24 [preprint]: 487. 1919. Type. Indonesia. [Java]: “am Berg Tamp” [protologue] East Java, in summo montis Taroeb [=Gunung Tarub], prov. Probolingo, 4 Jan 1845 [from P00369020], *H. Zollinger 2597* (lectotype, designated here: P [P00379534, labelled later as *2597 bis*]; isolectotypes: BM [BM001018925], G [G0343077, G000357860], G-DC [G00145616], P [P00369013, P00369020, as *2597Z*])).
Lycianthes laevis (Dunal) Bitter subsp. crassipetala (Wall.) Deb., Bot. J. Linn Soc. 76: 293. 1978. Type. Based on Solanumcrassipetalum Wall.
Lycianthes laevis (Dunal) Bitter subsp. bigeminata (Nees) Deb, Bot. J. Linn. Soc. 76: 293. 1978. Type. Based on Solanumbigeminatum Nees
Lycianthes laevis (Dunal) Bitter subsp. subtruncata (Wall. ex Dunal) Deb, Bot. J. Linn. Soc. 76: 293. 1978. Type. Based on Solanumsubtruncatum Dunal
Lycianthes laevis (Dunal) Bitter subsp. kaitisis (Dunal) Deb, Bot. J. Linn. Soc. 76: 294. 1978. Type. Baes on Solanumkaitisis Dunal
Lycianthes laevis (Dunal) Bitter var. gouakai (Dunal) Deb, Bot. J. Linn. Soc. 76: 294. 1978. Type. Based on Solanumgouakai Dunal
Lycianthes laevis (Dunal) subsp. kaitisis (Dunal) Bole & Ameida, J. Bombay Nat. Hist. Soc. 81(2): 378. 1984, nom. illeg., not Lyciantheslaevissubsp.kaitisis (Dunal) Deb (1978). Type. Based on Solanumkaitisis Dunal
Lycianthes marlipoensis C.Y.Wu & S.C.Huang, Acta Phytotax. Sin. 16(2): 78. 1978. Type. China. Yunnan: Malipo, 1,100–1,400 m, 13 Nov 1937, *G. Feng [Feng Guomei or K.M. Feng] 13227* (holotype: KUN [no acc. #]; isotype: PE [00633459]).
Lycianthes subtruncata (Wall.) Bitter var. paucicarpa C.Y. Wu & S.C.Huang, Acta Phytotax. Sin. 16(2): 79. 1978. Type. China. Yunnan: Longling, 1,600 m, 21De 1933, *H.T Tsai [Cai Xitao] 56685* (holotype: KUN [KUN1278691, acc. # 182540]; isotypes: A [00077124], LBG [00095865], PE [00031304],
Lycianthes crassipetala (Wall.) R.R.Mill, Edinburgh J. Bot. 57(3): 465. 2000, as “crassipetalum”. Type. Based on Solanumcrassipetalum Wall.

#### Type.

Based on *Solanumlaeve* Dunal

#### Description.

Shrubs or lax subshrubs, 1–3 m tall; stems terete, glabrous or variously pubescent with whitish grey translucent simple uniseriate 2–8-celled trichomes 0.5–1.5 mm long, these appressed or spreading, older stems glabrescent; new growth glabrous or sparsely to densely pubescent with translucent, simple, uniseriate 2–8-celled trichomes 0.5–1.5 mm long; bark of older stems glabrescent, yellow-green to greyish brown or dark brown. Sympodial units difoliate, the leaves geminate, the leaves of a pair differing in size and occasionally in shape, if different in shape slightly more ovate. Leaves simple; blades of major leaves (2–)5–22 cm long, (1–)2.5–11 cm wide, elliptic to occasionally narrowly elliptic, widest in the middle or just below, concolorous, but occasionally somewhat discolorous in live plants, membranous; adaxial surfaces glabrous or sparsely to moderately evenly pubescent with simple uniseriate trichomes like those of the stems, these denser along the veins, the lamina always visible; abaxial surfaces glabrous to moderately and evenly pubescent with simple uniseriate trichomes like those of the stems, if pubescent the lamina clearly visible; principal veins 5–10 pairs, glabrous or sparsely to moderately pubescent, the tertiary venation drying darker; base attenuate and somewhat decurrent onto the petiole; margins entire or in some populations (see discussion) toothed in the distal half, in pubescent plants ciliate with simple uniseriate trichomes like those of the stems to 1 mm long, if toothed the teeth to ca. 1 cm long, ca. 1.5 cm wide, broadly triangular with acute tips; apex acute to acuminate; petiole 0.9–3.5 cm long, glabrous or pubescent with simple uniseriate trichomes like those of the stems and new growth; blades of minor leaves (1–)3–10 cm long, (0.5–)1–6 cm wide, elliptic to narrowly elliptic or sometimes almost ovate; surfaces like those of the major leaves, the minor leaves often deciduous; principal veins of minor leaves 3–6 pairs; base attenuate or sometimes only acute; margins entire or very occasionally toothed, usually entire even when the margins of major leaves are toothed; apex acute to acuminate; petiole of minor leaves 0.5–1 cm long, glabrous or sparsely pubescent like the stems. Inflorescences axillary, in fascicles or on a fleshy stub ca. 1 mm long, with (1)4–10(16) flowers, glabrous or with a few trichomes at the pedicel bases; pedicels at anthesis 1–(1.5)2–3 cm long, ca. 0.5 mm in diameter at the base, ca. 1 mm in diameter at the base, ca. 1.5 mm in diameter at the apex, spreading, glabrous to variously pubescent with simple uniseriate trichomes like those of the stems, articulated at the base; pedicel scars tightly packed and overlapping. Buds ellipsoid, strongly tapered and pointed, the corolla never completely included, even in small buds, strongly exserted from the calyx tube before anthesis, the calyx appendages usually more apparent in bud. Flowers 5-merous, cosexual. Calyx tube 1.5–2.5 mm long, 2.5–3 mm wide at the mouth, obconical or an open cuplike structure, sometimes weakly 5-ridged, glabrous or sparsely pubescent with simple uniseriate trichomes like those of the pedicels, with 5 appendages arising at or ca. 0.5 mm below the hyaline rim, these more visible in bud, the appendages 0.5–4 mm long, ca, 0.5 mm wide, small nubs to curved and linear, usually erect or spreading and parallel to the calyx tube, glabrous or pubescent with a few simple uniseriate trichomes like those of the stems and pedicels. Corolla 1.3–2.5(3) cm in diameter, white to variously purple, stellate, lobed 3/4 or nearly to the base, interpetalar tissue mostly absent but a thin edge of tissue apparent on lobe margins, the lobes 6–9 mm long, 1.5–3.5 mm wide, spreading, glabrous on both surfaces, the tips and margins densely papillate, the tips somewhat cucullate. Stamens equal; filament tube minute; free portion of the filaments 0.5–1 mm long, glabrous; anthers 2.5–3.5 mm long, 1–2 mm wide, ellipsoid and tapering to a beak-like apex, tightly connivent, yellow, glabrous, poricidal at the tips, the pores tear-drop shaped and edged with white in dry material, lengthening to slits with age. Ovary conical, glabrous; style 5.5–10 mm long, exserted from the anther cone, glabrous; stigma prominently capitate, the surfaces minutely papillate. Fruit a globose berry, 0.8–1.3 cm in diameter, green when immature, bright red when mature, the pericarp glabrous, thin, shiny, and transparent at fruit maturity; fruiting pedicels 1.8–3 cm long, ca.1 mm in diameter at the base, ca. 2 mm in diameter at the apex, spreading or erect; fruiting calyx not accrescent or expanding, but remaining a plate-like structure, often reflexed below berry in dry specimens, the appendages spreading. Seeds 16–60 per berry, 2–2.5 mm long, 2–2.5 mm wide, flattened and triangular or somewhat tear-drop shaped, pale straw-colored or yellow, the surfaces deeply pitted, the testal cells rectangular to pentagonal, the lateral walls very thick, prominent “hairy” appendages absent. Stone cells absent. Chromosome number not known.

**Figure 12.**
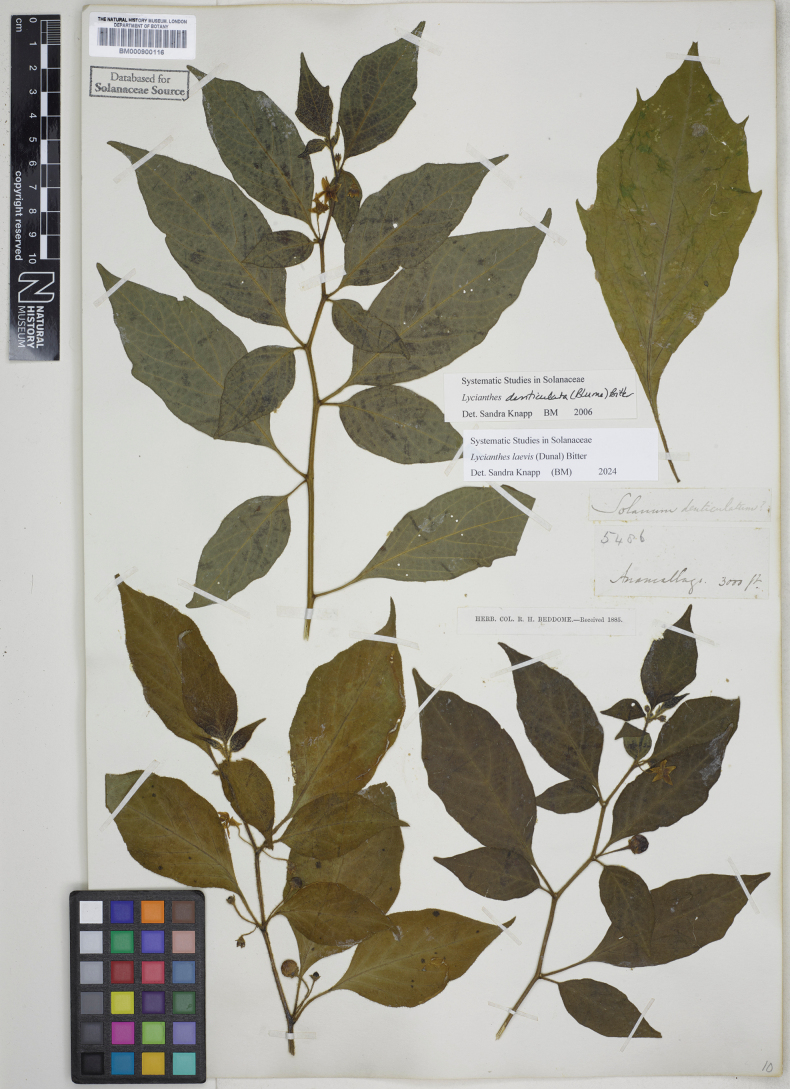
*Lyciantheslaevis* (Dunal) Bitter herbarium specimen. India. Tamil Nadu, *Beddome 5486* (BM00900116). Courtesy of the Trustees of the Natural History Museum, London, reproduced with permission.

#### Distribution

**Figure 13.**
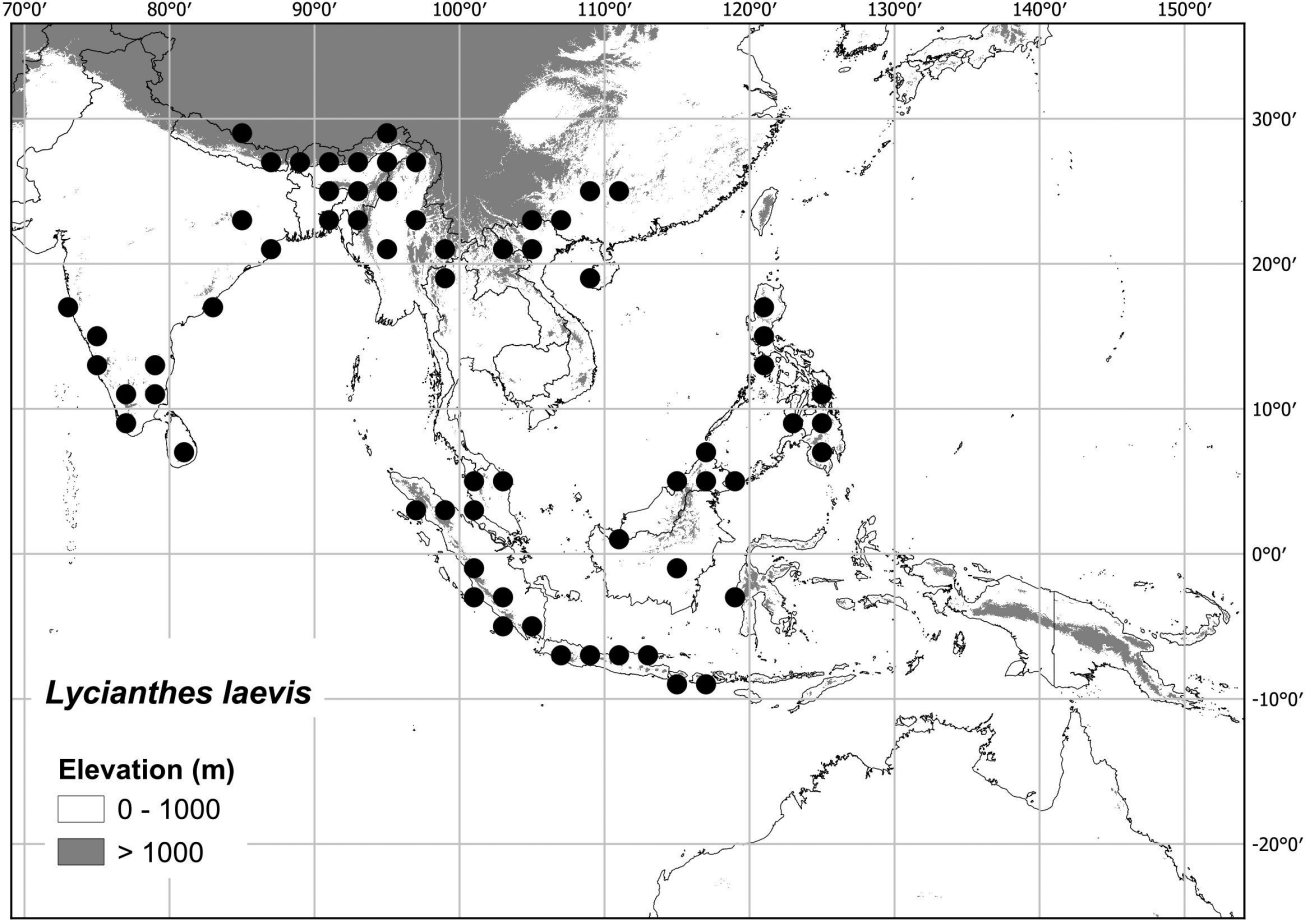
Distribution of *Lyciantheslaevis*.

**(Fig. 13).***Lyciantheslaevis* is widely distributed across tropical and subtropical Asia. It occurs in Bangladesh, Bhutan, Brunei Darussalam, China, India, Indonesia, Japan, Laos, Malaysia, Myanmar (Burma), Nepal, Philippines, Sri Lanka, Thailand and Vietnam.

#### Ecology and habitat.

*Lyciantheslaevis* grows in a wide variety of forest types from montane forest to evergreen and semi-evergreen forest to dry hillsides. It is often found in shrubby undergrowth or in light gaps and along paths, from (90–)500 to 2,100 m elevation. Most collections are from between 900 and 2,000 m elevation.

#### Common names.

China. ma po hong six ian (as *L.marlipoensis*), que chi hong si xian (Zhang et al. 1994). India. Mizaram: vainan (*Mrs. Parry 405*); Karnataka: kandukachi (*Raghavan 67850*); “Sikkim”: kanom bi bur, knombi (both *Anon. s.n*.); Tamil Nadu: gouakai (*Leschenault de la Tour 311*). Indonesia. bu-lum (Blume1823, as *S.violaceum*); Sumatra: ikkaoe-paet (*si Boeea 10641*), kajoe paga (*si Boeea 7082*), poga poga batoe (*si Boeea 10563*). Malaysia. bulung (Sundanese language, Burkill 1935, as *S.blumei*), tĕrong chator (language uncertain, Burkill 1935, as *S.blumei*); Selangor: bayma hutan (Temuan language, *Millard 1792*). Vietnam. cà ngủ cặp đôi (as *L.bigeminata*), cà ngủ nhẵn (Hop 2017). The leaves were eaten and cooked locally in northern India (*Anon. s.n.*). Burkill (1935) records in Java the use of leaves as a vegetable and the sweet, edible nature of the fruits; he also recorded the use of the seeds in cure of toothache but was unsure if it is this species being used for this purpose.

#### Preliminary conservation assessment

**(IUCN 2020).**EOO (15,152,928 km^2^ - LC); AOO (800 km^2^ - VU). *Lyciantheslaevis* is a weedy species known from more than five localities and is very widely distributed in the region. Throughout the range it is known from protected areas (e.g.,Gunung Gede Pangrango on Java, Kerinci Seblat on Sumatra, Indonesia; Western Ghats in Tamil Nadu, India). The assessment of Vulnerable (VU) based on AOO is likely due to collecting bias, but also due to the island nature of the distribution. I therefore assign it a preliminary status of Least Concern (LC).

#### Discussion.

*Lyciantheslaevis* is a widespread species with considerable regional variation. It can be recognised by its calyx with 5 appendages, these are usually quite short, but in southern India and Indonesia can be filiform and up to several mm long (Figs 3B, 4C). The calyx appendages always appear longer in bud. A collection from Mount Makiling in the Philippines (*Sulit 8499*) has a single flower with 7 appendages, but this is the only example of *L.laevis* I have seen with more than 5 calyx appendages. *Lyciantheslaevis* can be distinguished from other species in the area with 5 calyx appendages (e.g., *L.banahaensis*, *L.bimensis*) by its shrubby versus tree habit and in its cosexual flowers with membranous (versus thick and somewhat fleshy) corolla lobes. Like those of *L.schizocalyx*, the pedicels in fruit can be elongate, in both species reaching to 3 cm long. The berries of *L.laevis* are marginally smaller (0.8–1.3 cm in diameter versus 1–1.3 cm in diameter) but the number of calyx appendages (10 in *L.schizocalyx* versus 5 in *L.laevis*) is distinct.

Like *L.biflora*, pubescence density in *L.laevis* is extremely variable. Many specimens are nearly glabrous (including the type of *S.laeve*) whereas others (e.g., *Brandis 350*, the type of var. parvifrons) are densely and uniformly pubescent with simple uniseriate trichomes. I have never seen branched trichomes on plants of *L.laevis*, unlike in *L.biflora*, where they are common. Similarly, pubescent anthers have not been seen in *L.laevis*.

Flowers from some (but not all) populations in the north of the range in Nepal and northwestern India tend to be fleshier and somewhat larger than those from southern India and Sri Lanka and have been called *L.crassipetalum* (e.g., Mill 2001) When material is examined from across the area, however, this variation appears to be continuous or merely populational. This continuity has been noted by others, on a specimen of *L.laevis* in CAL (acc. # 315562, *Hooker & Thomson s.n.* from “Khasia”) an annotation by J.S. Gamble reads “intermediate between *S.subtruncatum* and *S.crassipetalum*” suggesting he too thought the variation in this species was continuous and these taxa were not distinct. Collections from Mount Salak in Java (Indonesia) also have large flowers (e.g., *Kuntze 4575*, *Kurz 462*); these may be virally infected but further field investigation is needed.

Leaves in some populations of *L.laevis* are shallowly lobed in the distal third with irregular, broad teeth arising from the vein endings (see Fig. 12); these have been called *S.gouakai* in southern India. Some local floras in southern India (Henry et al. 1987) recognise these leaf variants at the subspecific and varietal level in *L.laevis* using Dunal’s (1852) epithets, whereas others recognise a single taxon (*L.bigeminata*, Nair and Nayar 1987; *L.laevis*, Mohanan and Henry 1994). These leaf types occur most frequently in southern India (e.g., *Bourne 2088*) and Sri Lanka (*Walker 65*), but I have also seen collections with lobed leaves from western Myanmar (e.g., *Fujikawa et al. 094293*). Toothed and entire leaves occur on the same plant as is common in the Morelloid clade of *Solanum* (Knapp et al. 2023), in many species of spiny solanums (McClelland et al. 2020; Aubriot and Knapp 2022) and in *Lycianthes* series *Tricolores* Bitter of Mexico (Dean et al. 2017). Sometimes juvenile leaves in *Solanum* can be more lobed or toothed than leaves on mature branches (Roe 1966); this may be the case here, but it is not clear. Field surveys of these populations in Tamil Nadu would be very informative.

*Lyciantheslaevis* has a wide distribution, overlapping with most of the other *Lycianthes* species in Asia. It is more westerly than *L.biflora* or *L.schizocalyx* but does co-occur with both those taxa. Hybridisation is often cited as the reason for wide ranges of intraspecific variation, but the calyx appendage character is constant across the range of *L.laevis*; populations in sympatry should be studied in the field. One such, where *L.laevis* and *L.schizocalyx* co-occur and have been mixed in collections is on Mount Santo Tomas in the Philippines (e.g., *Williams 1334* and *1334[A*]). At first glance these plants look very similar, but closer examination show that not only do the calyx appendages differ (5 versus 10) but the pubescence also differs. The element of this gathering that represents *L.schizocalyx* was labelled with the same number (as a presumed duplicate) in pencil, possibly long after collection (see discussion of *L.schizocalyx*).

*Solanumlaeve* was described by Dunal (1814) based only on a collection seen by him in Paris from Java labelled “Bondolene” which Dunal took to be the local name. A specimen collected by J.B. Leschenault de la Tour in Java labelled “Goudolene” (misread by Dunal as Bondolene) does not bear a label in Dunal’s hand but corresponds to the protologue in being in fruit and is here selected as the lectotype (*Leschenault de la Tour 683*, P000578637). In the Prodromus (Dunal 1852), the collector of the type is mentioned.

Blume (1823) published the homonym *S.violaceum* Blume (not *S.violaceum* Ortega, an Asian spiny solanum; Aubriot and Knapp 2022) without citing a locality or herbarium. A sheet in Leiden collected by Blume in Java has an annotation “Solanum Blumii Nees” and a much later annotation as S.violaceum. I select this sheet (L 0003600) here as a neotype because there is no evidence that it was used by Blume in the description of *S.violaceum*, which could have been described from living material. Blume and Nees van Esenbeck were correspondents (van Steenis 1989) and the replacement name for the illegitimate *S.violaceum* Blume was coined by Nees van Esenbeck in Blume’s honour and published with a description by Blume (Blume 1826) as “Solanum blumii Nees ab Esenb.”. The annotation of “Solanum Blumii Nees” on the lectotype may be in Nees van Esenbeck’s hand.

Wallich’s designation “Solanummembranaceum” comprised three elements (Wallich 1828–1849: 80) was validated by Nees van Esenbeck (1834) where one of these (2625B from “herb. Wight”) was excluded and referred to *S.laeve.*Nees van Esenbeck (1834) indicated that only the specimens labelled 2625A and 2625C were part of his *S.membranaceum*. In the Wallich herbarium at Kew there are three specimens labelled 2625 (K001116640, K001116641, K0011166420) none of which bears the annotation corresponding to the catalogue entry. One of these is unambiguously labelled 2625A (K001116640) and has an annotation in Nees van Esenbeck’s hand and it also has the catalogue entry affixed to the sheet; I have selected this sheet as the lectotype of *S.membranaceum*. All three of these specimens are conspecific.

Nees van Esenbeck (1834) attributed the name *S.neesianum* to Wallich, but there is no mention of that name in Wallich’s catalogue (M. Watson, pers. comm. 7 Feb 2024). The specimen cited is “*Wall. Catal. Suppl. n. 248*/Crescit in montibus Silhet *Guil. Gomez*” suggesting the collector was William Gomez who had collected in the area and whose collections are listed on page 248 of the catalogue (https://wallich.rbge.org.uk/index.php?section=pages&id=1279). A specimen in Nees van Esenbeck’s personal herbarium now held in GZU is clearly original material (GZU000255706); it is labelled with the name, has an annotation “248” and a locality “Mt Sylhet FD”. There is clearly a mistake in the published protologue as to collector; “FD” indicates the specimen was collected by Francis de Silva, one of Wallich’s collectors in the Silhet region. In the absence of any more specific material, I have designated this sheet collected by Francis de Silva in GZU (GZU000255706) as the lectotype. I suspect this specimen belongs to the set “Wallich Cat. 2620”, collected at the same place by the same person (“Mont. Sillet, FD”) as the type of *S.subtruncatum.*

Dunal (1852) validated Wallich’s designation “Solanumsubtruncatum” citing the specimen “Wallich cat. 2620” from G-DC; Deb’s apparent lectotypification (by the citation of the collection Wallich cat. 2620 in CAL) of *S.subtruncatum* was not necessary. In describing *S.kaitisis*Dunal (1852) cited a collection *Perrottet 230* from Paris; the sheet labelled *Perrottet 230* (P00248294) corresponds to *Uvarianarum* (Dunal) Blume (Annonaceae). Dunal also worked with Annonaceae (Dunal 1817). I am treating the collection number *230* in the protologue as an error to be corrected to *Perrottet 270*, a sheet (P00058859) that is annotated by Dunal as *Solanumkaitisis*. Dunal (1852) described *S.gouakai* and its two varieties (var. angustifolium and var. latifolium) from a single collection made by J.B. Leschenault de la Tour in southern India (*Leschenault de la Tour 311*), that he saw in Paris. I have used the three sheets of this collection In P as lectotypes for the species and each of the two varieties; that chosen as the lectotype for var. angustifolium (P0057928) is annotated “α angustifolium” by Dunal, that chosen for var. latifolium (P00057929) is annotated “β latifolium”) by Dunal and that for the species is only annotated with the specific epithet (P00057927). The three sheets vary somewhat in leaf width but clearly are from the same gathering.

*Bassoviawallichii* is an illegitimate superfluous name for *S.crassipetalum* Wall. in that Dunal (1852) based the name on “Solanumcrassipetalum Wallich cat. 2618 in G-DC” (Turland et al. 2018: Art. 52.2(e)).

Of the two varieties of *S.blumei* described by Miquel (1864) I have found original material for only S.blumeivar.parvifolium. Two sheets of the specimen “S. Eschweilerianum Zipp. herb.” mentioned in the protologue are held in Leiden, one (L 0003598) is annotated by Miquel and is a better specimen and I have designated this the lectotype. The other is an isolectotype (L 0003599). I have found no unambiguous original material for var. grandifolium. Among material cited was something collected by “Horsf.” a reference to Thomas Horsfield who collected in Java (van Steenis-Kruseman 1985); a specimen of Horsfield’s corresponding to the protologue (BM001018973) is here selected as the neotype for S.blumeivar.grandifolium.

Clarke (1883) cited collections from “*Gardner, Wight, Thwaites*” in the protologue of S.bigeminatumvar.zeylanica but with no mention of collection numbers or herbaria. I have selected the sheet of *Gardner 628* from the Hooker herbarium at Kew (K000923382) as the lectotype for var. zeylanica, it is annotated as var. zeylanica in Clarke’s hand and has flowers and fruits. Other duplicates at Kew are less complete. Hepper (1987) mentioned a Gardner collection in his description of var. zeylanica, but without citing a herbarium or number.

Kuntze (1891) described two varieties for *S.blumei* based on fruit colour from his own collections. In the very short protologue for var. erythrocarpum he states the distribution as Java and Sikkim; I have selected a specimen from Kuntze’s herbarium (NY barcode 00172265) now at NY annotated by Kuntze from Sikkim as lectotype for this variety. For var. xanthocarpum he states the distribution as “Wilis” (=Wilisgebirge, or Mount Wilis in West Java); here a single specimen in NY from Kuntze’s herbarium (barcode 00172280) from the correct locality and annotated by Kuntze is selected as the lectotype for the variety.

Elmer (1915) cited a single collection (*Elmer 13828*) in the protologue of *S.mindanaense* but no herbarium; specimens upon which Elmer’s names were based were in his private collection at the Philippine National Herbarium (then the Philippine Bureau of Agriculture under United States jurisdiction) that was destroyed by fire during the Japanese occupation of the Philippines in the Second World War just a day before the liberation of Manila (Schultes 1957). The only duplicate I have seen is a sterile specimen at Edinburgh (E00273869); intensive searches at other herbaria where Elmer sent collections have not yet been successful. Elmer suggested his plant differed from *S.crassipetalum* (=*L.laevis*) in “a number of minor characters” (Elmer 1915: 2833). I place *S.mindanaense* in synonymy here based on the description but will continue to search for a fertile duplicate with which to lectotypify this name.

Bitter (1919) described three infraspecific taxa for L.bigeminata from India; subsp. nodocalyx , var. parvifrons and var. calycodonta. Hepper (1987) lectotypified L.bigeminatasubsp.nodocalyx with the collection *Hohenacker 803* but in citing both K and SLO necessitated a second-step lectotypification here. I have selected the specimen at Kew (K00759401) as the second-step lectotype. For L.bigeminatavar.parvifronsBitter (1919) cited many different collections (*Wight 2021*, *2025*, *1569*; *Hooker & Thomson s.n*; *Brandis 350*, *s.n.*). The collections of Robert Wight are widely distributed, but whether they are real duplicates is not clear (Noltie 2005). I have therefore selected *Brandis 350* in Hamburg (HBG-511357), cited as from “herb. Hamb.” and annotated by Bitter, as the lectotype for var. parvifrons. Many collections were also cited in the protologue of var. calcodonta mostly from “herb. Berol.” (*Hohenacker 1415*; *Thomson s.n.*; *Meebold 8878*, *9456*, *11696*, *12346*; *Saulière 88*), plus a sheet of *Wight 2012* from Copenhagen. I have selected a duplicate of *Meebold 8878* held in Calcutta (CAL acc. # 315662) as the lectotype for var. calycodonta; it is a well-preserved specimen and is held in an Indian herbarium.

As part of his concept of *L.laevis*Bitter (1919) published several infraspecific taxa. For L.laevisvarbrevipedicellata he cited a specimen from Bogor “Sumatra Poeding zimbo! hb. Bogor” of which no material has been found despite an extensive search in BO and in Leiden. I have not neotypified this name here, as material may be found in the future. The protologue of L.laevissubsp.inaequidens includes citations of duplicates of *Zollinger 2597* from Bogor and Paris (although he cites the Berlin specimen of *Zollinger 2597* under *L.laevis*) together with a number of collections seen from “hb. Bog.” Searches at BO have turned up only *Koorders 19891* and *31886* of all the collections cited; both are scrappy specimens with little reproductive material. The sheet at Paris labelled *Zollinger 2597 bis* with an undated annotation slip by Bitter (P00379534) is therefore selected as the lectotype for var. inaequidens. All the sheets of *Zollinger 2597* I have seen appear to be from the same gathering (although only P000369020 from the Drake herbarium in P, labelled “2597 Z” in an unknown hand, has the original locality label) and I consider *S.zollingeri* var. multiflorum and L.laevissubsp.inaequidens to be homotypic.

### ﻿﻿5. Lycianthes lysimachioides

Taxon classificationPlantae

(Wall.) Bitter, Abh. Naturwiss. Verein Bremen 24 [preprint]: 491. 1919.

A17CFAFB-6EE4-54AB-9051-0E8F375F40C1

Figs 2C , 14

Solanum lysimachioides Wall., Fl. Ind. (Carey & Wallich ed.) 2: 257. 1824. Type. Nepal. [Bagmati prov.]: “Chondaghery” [Chandragiri], Feb 1821, *N. Wallich Cat. 2609* (lectotype, designated by Deb 1978, pg. 293 [as “type”]: CAL [CAL0000071943, acc. # 315728]; isolectotypes: BM [BM001018867], K [K000923249], K-W [K001116562, K001116563]).
Solanum caulorhizum Dunal, Prodr. [A. P. de Candolle] 13(1): 181. 1852. Type. Indonesia. Java: “prov. Badong”, 1847, *H. Zollinger 705* (holotype: G-DC [G00145653]; isotypes: BM [BM000778224], G [G00301677], MPU [MPU012644], P [P00369006]).
Solanum nematosepalum Miq., Fl. Ned. Ind. 2: 643. 1857. Type. “Solanum ciliatum Blume in herb. Reg. L.B.” (no specimens cited or located); Indonesia. [Java]: sin. loc., C.L. Blume s.n. (neotype, designated here: L [L.2881683]).
Solanum macrodon Wall. ex Nees var. lysimachioides (Wall.) C.B.Clarke, Fl. Brit. India [J. D. Hooker] 4: 232. 1883. Type. Based on Solanumlysimachioides Wall.
Lycianthes lysimachioides (Wall.) Bitter var. caulorhiza (Dunal) Bitter, Abh. Naturwiss. Verein Bremen 24 [preprint]: 493. 1919. Type. Based on Solanumcaulorhizum Dunal
Lycianthes lysimachioides (Wall.) Bitter var. sinensis Bitter, Abh. Naturwiss. Verein Bremen 24 [preprint]: 493. 1919. Type. China. Hubei: sin. loc., 1885, *A. Henry 6080* (lectotype, designated here: BM [BM001018842]; isolectotypes CAL [acc. # 316339, 316340], E [E00426492], GH [no barcode],US [02840640, acc. # 801545]).
Lycianthes lysimachioides (Wall.) Bitter var. formosana Bitter, Repert. Spec. Nov. Regni Veg. 18: 320. 1922. Type. Taiwan. (“Formosa”) Arisan, 2,500 m, 1914, *U. Faurie 640* (holotype: G [G00415805]; isotypes: BM [BM001018839], GH [no barcode]).
Solanum debilissimum Merr., Philipp. J. Sci. 23: 265. 1923. Type. China. Hainan: “Ng Chi Leng [Five-Finger Mountain]”, 11 May 1922, *F.A. McClure 9532* (no herbarium cited; lectotype, designated here: E [E00426499]; isolectotypes: A [0077823], BISH [BISH1005077, acc. # 182918], BM [BM001018850], K [K000759408], MO [MO-503598, acc. # 915771]).
Numaeacampa kerrii Gagnep., Bull. Soc. Bot. France 95(1): 33. 1948. Type. Laos. Vienchan: Pu Tat (Phu That), “Wiengchan” [Vienchan], 21 Apr 1932, *A.F.G. Kerr 21186* (holotype: P [P00236810]; isotypes: BM [BM001018901], K [K000923331, K000923332]).
Lycianthes solitaria C.Y.Wu & A.M.Lu, Acta Phytotax. Sin. 16(2): 76. 1978, nom. illeg., not Lycianthessolitaria Standl. (1927). Type. China. Tibet: Zayu, 1,700 m, 14 Jul 1973, *Qinghai-Tibet group 73-672* (holotype: KUN [acc. # 484269]; isotype: KUN [acc. # 484270]).
Lycianthes lysimachioides (Wall.) Bitter var. purpuriflora C.Y.Wu, Acta Phytotax. Sin. 16(2): 79. 1978. Type. China. Sichuan: Emei, 1,200 m, 26 May 1935, *Du Dahua 418* (lectotype: PE [00031385]; isolectotypes: IBK [IBK00381736], PE [00013186, 0013187, 0031388]).
Lycianthes lysimachioides (Wall.) Bitter var. cordifolia C.Y.Wu & S.C.Huang, Acta Phytotax. Sin. 16(2): 79. 1978. Type. China. Sichuan: Emei, 1952, *J. Xiong [Xiong Jihua], X. Zhang [Zhang Xiushi] & X. Jiang [Jiang Xinglin] 32197* (holotype: PE [00031390, acc. # 267671]).
Lycianthes lysimachioides (Wall.) Bitter var. rotundifolia C.Y.Wu & S.C.Huang, Acta Phytotax. Sin. 16(2): 79. 1978. Type. China. Shaanxi: Xianyang, 1,000 m, 23 Sep 1933, *T.P. Wang [Wang Zuobin] 857* (lectotype, designated here: PE [00031306]; isolectotype: PE[00031307]).
Lycianthes tibetica Li Bing Zhang & Yi F.Duan, Phytotaxa 170(4): 280. 2014. Type. Based on (replacement name for) Lycianthessolitaria C.Y.Wu & A.M.Lu

#### Type.

Based on *Solanumlysimachioides* Wall.

#### Description.

Prostrate or creeping herbs, the stems to 1.5 m long, occasionally described as subshrubs to 1 m; stems terete, collapsing as if hollow in most specimens, rooting at the nodes or near the nodes along the stem, densely pubescent to glabrescent, if pubescent the trichomes whitish transparent, simple, uniseriate 5–8-celled, to 2 mm long; new growth glabrous to densely pubescent with whitish transparent, simple uniseriate 5–8-celled trichomes to 2 mm long; surfaces (bark) of older stems glabrescent to variously pubescent, the trichomes sparser on older stems, pale greyish brown or greenish brown. Sympodial units difoliate, the leaves geminate, the leaves of a pair differing in size and occasionally in shape, when minor leaves are similar in size the leaves appear opposite. Leaves simple; blades of major leaves 1.8–14 cm long, 1.4–8.7 cm wide, elliptic to ovate (occasionally narrowly elliptic), widest in the middle or in the lower half, concolorous but occasionally purple or purple-veined abaxially (e.g., *Sino-American Guizhou Botanical Expedition 1240*), thin and membranous; adaxial surfaces glabrous to evenly and sparsely to moderately pubescent with simple uniseriate trichomes like those of the stems, these denser along the veins, the lamina always visible; abaxial surfaces glabrous to evenly and moderately pubescent with simple uniseriate trichomes like those of the stems, the lamina clearly visible, the trichomes denser on the principal veins and midrib; principal veins 3–5 pairs, glabrous or variously pubescent, the venation not markedly coloured; base attenuate to truncate to cordate; margins entire, in pubescent plants ciliate with simple uniseriate trichomes to 1 mm long; apex acuminate; petiole 0.9–6.3 cm long, glabrous or sparsely pubescent with simple uniseriate trichomes like those of the stems and new growth; blades of minor leaves 0.8–9 cm long, 0.6–5.5 cm wide, elliptic to ovate, sometimes almost orbicular; surfaces like those of the major leaves; principal veins of minor leaves 3–4 pairs; base attenuate to truncate or cordate; margins entire; apex acute to acuminate; petiole of minor leaves 0.4–2.1 cm long, glabrous or sparsely pubescent like the stems. Inflorescences axillary, in fascicles, with 1(2) flowers, glabrous; pedicels at anthesis 0.6–2.3 cm long, ca. 0.5 mm in diameter at the base, 1–1.5 mm in diameter at the apex, spreading and sometimes apparently bent (geniculate) in the upper third, glabrous to pubescent with simple uniseriate trichomes like those of the stems, articulated at the base; pedicel scars tightly packed in the leaf axils. Buds strongly tapered and pointed, the corolla strongly exserted from the calyx tube before anthesis, the calyx appendages enclosing the bud only partially. Flowers 5-merous, cosexual. Calyx tube 1.9–2.5 mm long, 2.5–3.5 mm wide at the mouth, obconical or an open cuplike structure, 10-ridged, glabrous or pubescent with simple uniseriate trichomes like those of the pedicels, the trichomes often denser on the ridges, with 10 appendages arising ca. 0.5 mm below the hyaline rim, the appendages 3–6 mm long, 0.5–0.7 mm wide, subulate, parallel to the calyx tube, glabrous or pubescent with simple uniseriate trichomes like those of the stems and pedicels. Corolla 1.8–3 cm in diameter, white, “pink” or pale violet (lilac), with green dots at the base of the lobes, stellate, lobed nearly to the base, interpetalar tissue absent but a thin edge of tissue apparent on lobe margins, the lobes 8–15 mm long, 2.5–3.5 mm wide, spreading, glabrous on both surfaces, the tips and margins sparsely and minutely papillate. Stamens equal; filament tube minute; free portion of the filaments 0.75–1 mm long, glabrous; anthers 2–4 mm long, 0.75–1.5 mm wide, ellipsoid and tapering to a beak-like apex, tightly connivent, yellow, glabrous, poricidal at the tips, the pores tear-drop shaped and edged with white in dry material, lengthening to slits with age. Ovary conical, glabrous; style 4–9 mm long, exserted from the anther cone, glabrous; stigma prominently capitate, the surfaces minutely papillate. Fruit a globose berry, 0.6–0.8 cm in diameter, green when immature, bright red when mature, the pericarp glabrous, thin, shiny, and transparent; fruiting pedicels 1–3 cm long, 0.5–1 mm in diameter at the base, 1.5–2 mm in diameter at the apex, spreading; fruiting calyx not accrescent or expanding, but remaining a cup covering ca. 1/4 of the berry, the appendages spreading like a star beneath the berry. Seeds 10–40 per berry, 2–2.5 mm long, 1.2–2 mm wide, flattened-reniform, pale straw-coloured or yellowish tan, the surfaces pitted, the testal cells sinuate in outline in the centre, rectangular on the margins, prominent “hairy” appendages absent. Stone cells absent. Chromosome number not known.

**Figure 14.**
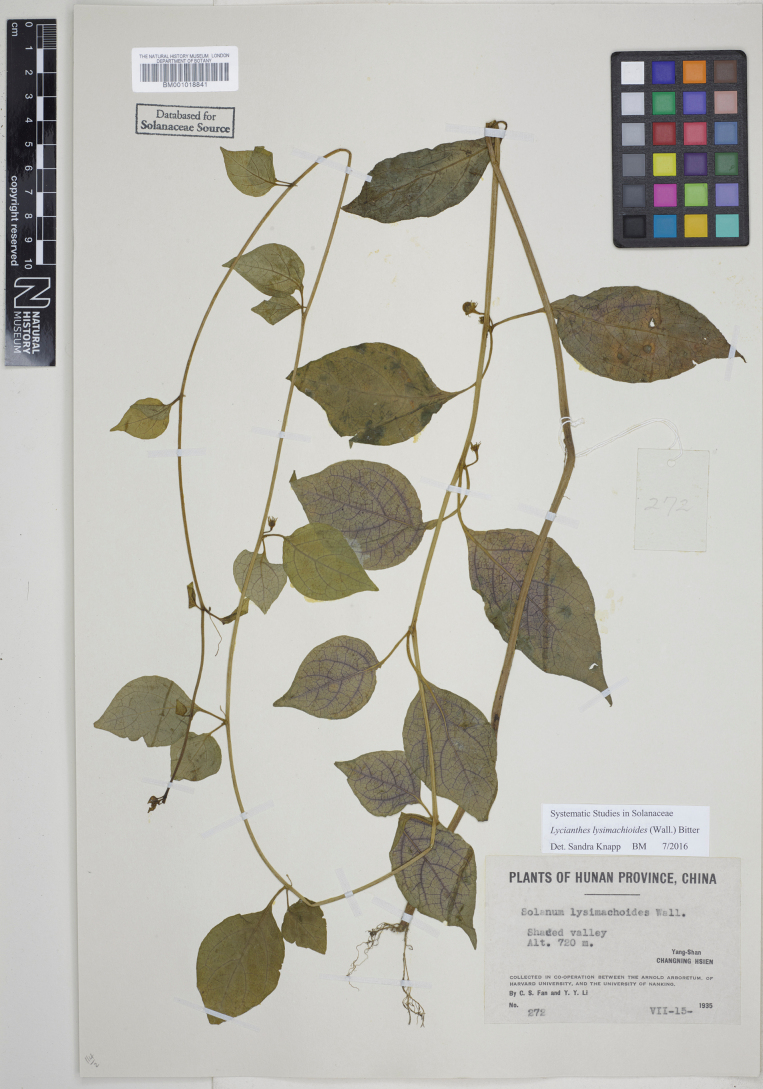
*Lyciantheslysimachioides* (Wall.) Bitter herbarium specimen. China. Hunan, *Fan & Li 272* (BM001018841). Courtesy of the Trustees of the Natural History Museum, London, reproduced with permission.

#### Distribution

**Figure 15.**
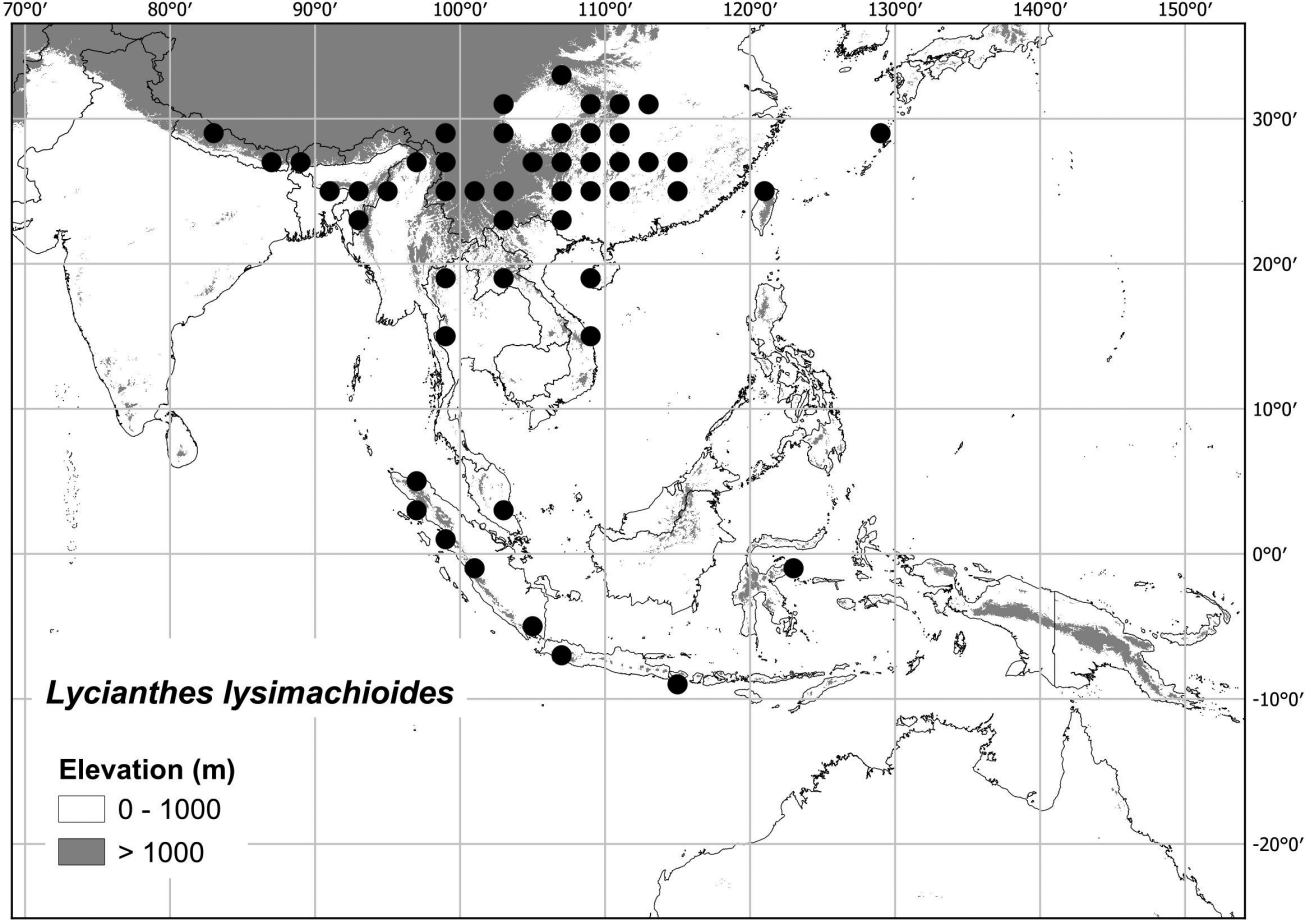
Distribution of *Lyciantheslysimachioides*.

**(Fig. 15).***Lyciantheslysimachioides* occurs in China, India, Indonesia, Japan, Laos, Myanmar (Burma), Nepal, Taiwan, Thailand and Vietnam. I have not seen specimens from Cambodia, but it is likely to occur there.

#### Ecology and habitat.

*Lyciantheslysimachioides* is usually found growing in deep shade in damp places along streams or in forest understory, often as dense patches, from 600 to 2,400 m elevation.

#### Common names.

China. dan guo hong si xian (as *L.solitaria*), dan hua hong si xian (Zhang et al. 1994). Indonesia. Sulawesi: kamoenti (Koorders 1898, as *S.lysimachioides*). Vietnam. cà ngủ dạng trân châu (Hop 2017).

#### Preliminary conservation assessment

**(IUCN 2020).**EOO (14,512,768 km^2^ - LC); AOO (372 km^2^ - EN). *Lyciantheslysimachioides* is a species known from more than ten localities and is quite widely distributed in the region. Throughout the range it is known from protected areas (e.g., Upper Shillong Protected Forest in India; Emeishan in Sichuan, China). The assessment of Endangered (EN) based on AOO is likely due to collecting bias, but also due to the island nature of the region. I therefore assign it a preliminary status of Least Concern (LC).

#### Discussion.

*Lyciantheslysimachioides* is distinctive amongst Asian species of *Lycianthes* in being a creeping herb of forest understories and shady places (Fig. 2C); it only occasionally attains shrubby proportions, but if so then it is less than a metre tall. The specific epithet reflects its striking similarity with species of *Lysimachia* L. (Primulaceae). The geminate leaves of *L.lysimachioides* can be mistaken for the opposite leaves of *Lysimachia* and coupled with the creeping habit, leads to confusion in herbarium specimens. I have seen several specimens of *Lysimachiaevalvis* Wall. identified as *Lyciantheslysimachioides*; the plants are very similar at first glance (see image of K0007507178 at https://powo.science.kew.org/taxon/urn:lsid:ipni.org:names:701116-1). Like most of the species treated here, it is extremely variable in pubescence density, with some individuals densely pubescent and others nearly glabrous. Trichomes of *L.lysimachioides* are simple and uniseriate, and usually composed of many small cells, I have never seen branched trichomes in this species. Leaf shape in *L.lysimachioides* is quite variable, some specimens have almost orbicular, cordate leaves (Fig. 2C), whereas others are almost narrowly elliptic with acute or attenuate bases. Most of the infraspecific taxa placed in synonymy here have been based on pubescence or leaf shape differences. There appears to be no particular geographical or habitat correlation with leaf shape or pubescence density.

*Lyciantheslysimachioides* is sometimes mistaken for *L.biflora* but is a creeping or scandent herb rooting at the nodes rather than an erect woody shrub, has a single flower per fascicle (only rarely more) and larger flowers (corolla 1.8–3 cm versus 1.4–1.8 cm in diameter in *L.biflora*). Identification of sterile or poor specimens of *L.lysimachioides* can be helped by looking for roots at the nodes and stems that collapse in dried specimens. The single collection I have seen from peninsular Malaysia (*Henderson SF-22378* from Pahang) is somewhat woody but has clear roots emerging at the nodes and along the stems. Plants identified and depicted as *L.lysimachioides* from Manipur, northeastern India by Eshuo (2023) are part of the concept of *L.biflora* as treated here.

*Lycianthesschizocalyx* has similar calyx appendages in which at least some arise below the calyx rim leaving a hyaline edge; it can be distinguished from *L.lysimachioides* by its shrubby habit, calyx appendages of variable length in a single flower and larger berries (1–1.3 cm versus 0.6–0.8 cm in diameter.

Dunal (1852) described *Solanumcaulorhizum* based on the collection *Zollinger 705* in the de Candolle herbarium (G-DC); a duplicate in the Paris herbarium (P00369007) is a specimen of *L.biflora* and is excluded from the isotypes here (this sheet has been designated *Zollinger 705 [a*] in the material examined).

*Solanumnematosepalum* was described only referring to a specimen “Solanum ciliatum Blume in herb. reg. L.B.”(Miquel 1864) from Java. Searches in both Bogor and Leiden have revealed no specimens so labelled, so I here designate a neotype from the Blume herbarium (L.2881683) collected in Java that corresponds to the protologue, represents this species and is labelled in what looks like Miquel’s handwriting.

In his description of L.lysimachioidesvar.sinensisBitter (1919) cited a number of collections (*Henry 7207*, *3063*, *6080*; *Wilson 1374*, *2158*; *Rosthorn s.n*.) all from “herb. Berol.” From these large-leaved specimens I have selected *Henry 6080* (BM001018842) as the lectotype, as this is the best-preserved specimen, and duplicates of this collection are widely distributed.

Merrill (1923) cited no herbaria in his description of *S.debilissimum*, and only a single collection, *McClure 9532*. Material Merrill examined may have been destroyed in Manila (see under *L.banahaensis*) but duplicates of this material are widely distributed. I have selected the sheet in Edinburgh (E00426499) as the lectotype for this species, as this specimen has flowers and a developing fruit, whereas others have only flowers or are sterile.

The varieties of *L.lysimachioides* described by Wu and Huang (1978) are generally referred to only using the word “type” and cite only the herbarium where the type is housed (i.e., var. purpurifolia, var. rotundifolia); where multiple sheets are housed in the cited herbaria (i.e., PE) I have selected that annotated as “holotype” as the lectotype. Cases where only one specimen is housed in the cited herbarium are clear.

### ﻿﻿6. Lycianthes oliveriana

Taxon classificationPlantae

(Lauterb. & K.Schum.) Bitter, Abh. Naturwiss. Vereins Bremen 24 [preprint]: 504. 1919, as “ Oliveriana”.

1916794A-5F13-57E1-A3A4-033328A1C9C6

Figs 3D , 16

Solanum oliverianum Lauterb. & K.Schum., Fl. Schutzgeb. Südsee [Schumann & Lauterbach] 535. 1901, as “Oliverianum”. Type. Papua New Guinea. Sanduan/East Sepik: “Kaiser Wilhelmsland, Augustafluss”, Sep 1887, *M. Hollrung 776* (lectotype, designated by Symon 1985, pg. 56: K [K000759399]; isotypes: HBG [HBG-511470], L [L0003651], LE [LE00016994], MEL [MEL104160], P [P00379610]).
Solanum memecylonoides Bitter & Schltr., Bot. Jahrb. Syst. 55: 93. 1917. Type. Papua New Guinea. Sanduan: “Kaiser Wilhelmsland, Torricelli-Geb[irges]”, 800 m, 18 Sep 1909, *F.R.R. Schlechter 20256* (holotype: B [destroyed]; lectotype, designated by Knapp 2022, pg. 81: P [P00379576]; isolectotype: BR [BR0000005528844]).
Solanum memecylonoides Bitter & Schltr. var. finisterrae Bitter, Bot. Jahrb. Syst. 55: 94. 1917. Type. Papua New Guinea. Madang: “Kaiser Wilhelmsland, Finisterre-Gebirge”,1,000 m, 3 Jul 1908, *F.R.R. Schlechter 17961* (holotype: B [destroyed]; lectotype, designated by Knapp 2022, pg. 81: P [P00379575]; isolectotype: UC [cited by Symon 1985, not seen nor on UC/JEPS database]).
Solanum balanidium Bitter, Bot. Jahrb. Syst. 55: 95. 1917. Type. Papua New Guinea. East Sepik: “Hunsteinspitz” [Mount Hunstein], 1300 m, Feb-Mar 1913, *C.L. Ledermann 11332* (holotype: B [destroyed]). Papua New Guinea. East Sepik: Hunstein range, (Mt. Samsai) at site “Camp 3” on slopes above main streamcourse, 450 m, 17 Jul 1990, *W.N. Takeuchi 6156* (neotype, designated by Knapp 2022, pg. 81: LAE [acc. # 293351]; isoneotypes: A [00619947, 00619957], BISH [acc. # 618017], K [K001153745, K000922457, K000922458], L [L.2881432, L.2882048], MO [acc. # 4235181], NSW [NSW825821], NY [01404956, 02286515], US [01253664, acc. # 3723521]).
Solanum ledermannii Bitter, Bot. Jahrb. Syst. 55: 107. 1917, as “Ledermannii”. Type. Papua New Guinea. East Sepik: “Etappenberg” [between Kamelrücken and Bambooberg 4°38'S, 142°29'E, fide Veldkamp et al. 1988], 850 m, Oct 1912, *C.L. Ledermann 9214* (holotype: B [destroyed]). Papua New Guinea. East Sepik: Amboin, Angoram subdistrict, 90 m, 29 Jul 1967, *A.N. Millar & A.W. Dockrill NGF-35176* (neotype, designated by Knapp 2022, pg. 81: LAE [acc. # 89947]; isoneotypes: BRI [n.v.], L [L.2881436]).
Lycianthes balanidium (Bitter) Bitter, Abh. Naturwiss. Vereins Bremen 24 [preprint]: 504. 1919. Type. Based on Solanumbalanidium Bitter
Lycianthes ledermannii (Bitter) Bitter, Abh. Naturwiss. Vereins Bremen 24 [preprint]: 504. 1919, as “Ledermannii”. Type. Based on Solanumledermannii Bitter
Lycianthes memecylonoides (Bitter & Schltr.) Bitter, Abh. Naturwiss. Vereins Bremen 24 [preprint]: 504. 1919. Type. Based on Solanummemecyloniodes Bitter & Schltr.

#### Type.

Based on *Solanumoliverianum* Lauterb. & K.Schum.

#### Description.

Woody climbers or lianas, sometimes described as shrubs, to 3+ m tall (often described on labels “beautiful” e.g., *van Royen & Sleumer 7716* from New Guinea); stems terete, glabrous; new growth glabrous or minutely papillate with tiny 1–2-celled weak simple uniseriate trichomes less than 0.2 mm long, these soon deciduous; bark of older stems whitish grey, peeling and flaking, somewhat rugose and thick. Sympodial units difoliate, the leaves geminate, the leaves of a pair differing in size but not in shape. Leaves simple; blades of major leaves (6.5)9–25 cm long, (2.8)3–10 cm wide (perhaps larger but not collected), elliptic, slightly discolorous, thick and coriaceous or chartaceous; adaxial surfaces glabrous, somewhat shiny; abaxial surfaces glabrous; principal veins 6–8 pairs, the midrib slightly keeled above, sometimes drying yellowish tan; base acute, often somewhat oblique; margins entire, revolute; apex acute or acuminate with an elongate drip-tip; petioles 1–2.5 cm long, glabrous; blades of minor leaves 4–9 cm long, 2.5–5 cm wide, shape, texture and pubescence like that of the major leaves; base acute; margins entire, revolute; apex acute or acuminate, occasionally rounded; petioles 0.6–1 cm long, glabrous. Inflorescences dense axillary fascicles, occasionally woody and enlarged with what appear to be short, fat branches, these to 0.3 cm long, with 10–20-flowers, several open at the same time, glabrous; pedicels at anthesis 1–1.4 cm long, ca. 0.5 mm in diameter at the base, ca. 1 mm in diameter at the apex, spreading, glabrous, articulated at the base; pedicel scars tightly packed on the woody fascicle base. Buds plumply ellipsoid, the corolla ca. halfway exserted from the calyx tube before anthesis. Flowers 5-merous (4-merous in *Takeuchi 23389*), heterostylous, specimens with either all short-styled flowers or long-styled flowers and fruit, the plants probably dioecious. Calyx tube 2.5–3 mm long, 3–3.5 mm in diameter, deeply cup-shaped, usually described as purple or purplish blue, thick and fleshy, densely verrucose/tuberculate, without appendages, the rim somewhat hyaline ca. 0.5 mm wide, sparsely papillate. Corolla 0.8–1.1 cm in diameter, white or purple, stellate, lobed nearly to the base, interpetalar tissue absent, the lobes 2–5 mm long, 1–2 mm wide, spreading or reflexed, thick and fleshy (live plants), appearing woody in dry material, adaxially glabrous to densely papillate with a few weak trichomes distally, abaxially densely papillate somewhat verrucose, the tips and margins densely papillate, the midvein raised especially adaxially, the tips cucullate. Stamens equal; filament tube minute; free portion of the filaments 1–1.5 mm long, glabrous; anthers 2–2.5 mm long, ca. 1 mm wide, plumply ellipsoid or slightly obovoid, creamy white, yellow or purple, glabrous, poricidal at the tips, the pores round, distally directed, not elongating with age. Ovary conical, glabrous, vestigial in short-styled flowers; styles less than 0.2 mm long and vestigial in short-styled flowers, 5–6 mm long in long-styled flowers, straight, glabrous; stigma slightly bilobed, the surfaces minutely papillate. Fruit a globose berry, 0.7–1 cm in diameter, green and becoming bluish black when ripe, the pericarp glabrous, thick and appearing woody in dry material, matte, opaque; fruiting pedicels 1.1–1.5 cm long, 1–1.5 mm in diameter at the base, 1.5–2 mm in diameter at the apex, spreading or erect (?), woody, corky and markedly verrucose/tuberculate; fruiting calyx a cup surrounding ca. the lower half of the berry (making the fruit look like an acorn), woody (fleshy in live plants) and verrucose/tuberculate both adaxially and abaxially, green flushed with purple (fide *Polak 864*). Seeds 20–40 per berry, 3–3.5 mm long, 2–2.5 mm wide, flattened reniform or slightly tear-drop shape, reddish brown, the surfaces at the margins deeply pitted with pentagonal testal cells, the seed centre only shallowly pitted and the cells not clear, prominent “hairy” testal cell walls absent. Stone cells absent. Chromosome number not known.

**Figure 16.**
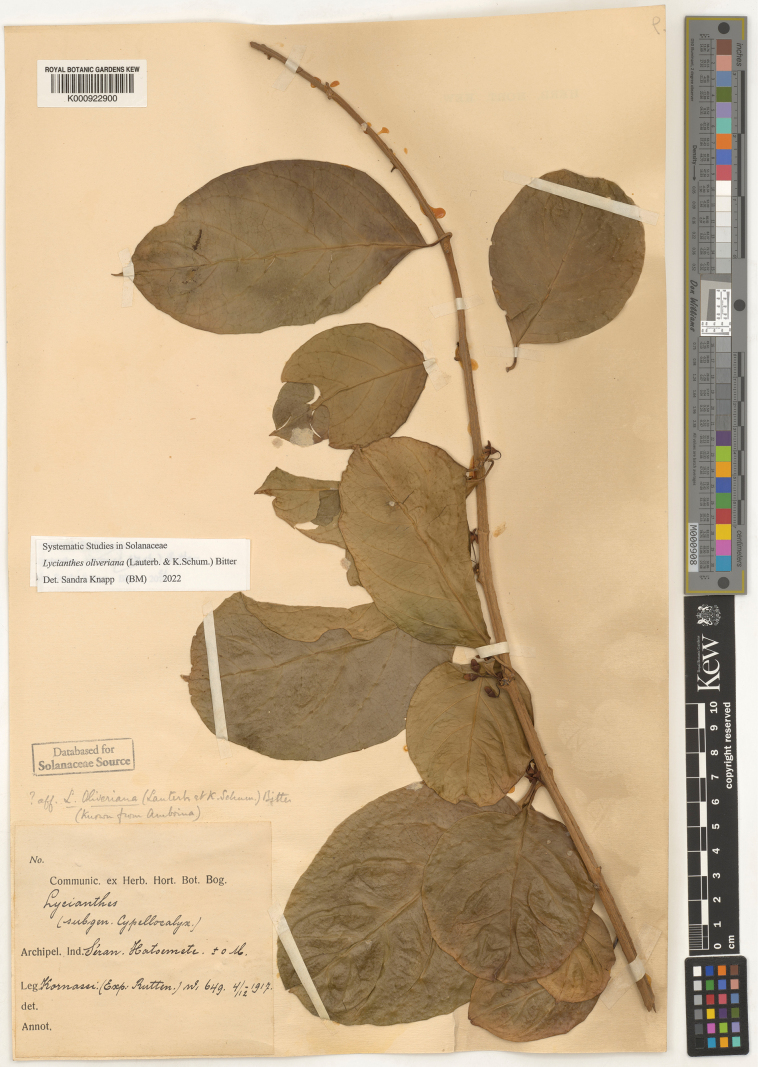
*Lycianthesoliveriana* (Lauterb. & K. Schum) herbarium specimen. Indonesia. Maluku, *Kornassi 649* (K000922900). Copyright Board of Trustees of the Royal Botanic Garden, Kew, reproduced with permission. http://specimens.kew.org/herbarium/K000922900.

#### Distribution

**Figure 17.**
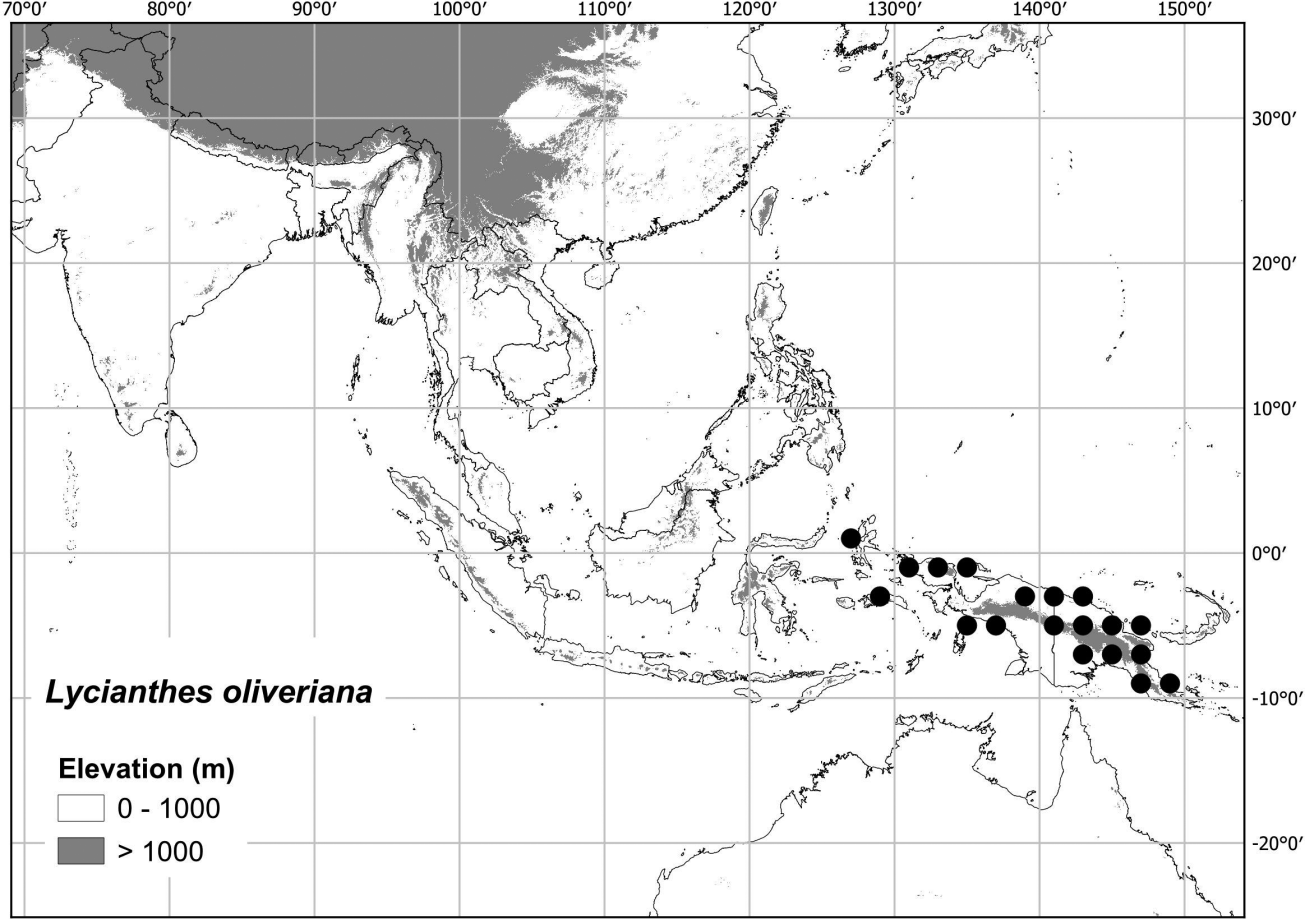
Distribution of *Lycianthesoliveriana* (including distribution on the island of New Guinea).

**(Fig. 17).** In Asia *Lycianthesoliveriana* occurs only in Indonesia on the Maluku Islands (Seram [Maluku] and Halmahera [Maluku Utara]) but is widespread on the island of New Guinea in both Papua New Guinea and Indonesia (Knapp 2022).

#### Ecology and habitat.

*Lycianthesoliveriana* grows in lowland to montane and premontane rainforests, from almost sea level to 2,300 m elevation. On New Guinea this wide elevational range is accompanied by much variation in leaf size and shape (Knapp 2022).

#### Common names.

None recorded.

#### Preliminary conservation assessment

**(IUCN 2020).**EOO (1,006,290 km^2^ - LC); AOO (156 km^2^ - EN). *Lycianthesoliveriana* is known from more than 10 localities at a wide variety of elevations; the assessment here is based on its entire distribution. It occurs within protected areas in both Indonesia and Papua New Guinea (see Knapp 2022). This suggests a preliminary threat status of either Least Concern (LC) or Near Threatened (NT).

#### Discussion.

*Lycianthesoliveriana* is a distinctive species with many-flowered axillary inflorescences, relatively small (ca. 1 cm in diameter) flowers with valvate aestivation and thick fleshy corollas, plump, ellipsoid to slight obellipsoid anthers and somewhat warty calyces with no appendages (Fig. 3D). The berries appear to have a slightly woody pericarp; this woodiness coupled with the somewhat accrescent calyx tube gives the fruits of *L.oliveriana* the look of tiny acorns. *Lycianthesoliveriana* appears to be dioecious, with long- and short-styled flowers on different plants. This needs confirmation in the field, and *L.oliveriana* would be an ideal subject for a reproductive biology study since it is relatively common and widely distributed.

*Lycianthesoliveriana* could potentially be confused with *L.parasitica*, a species with a wider distribution in tropical Asia outside of New Guinea. Berries of *L.oliveriana* are somewhat woody on dry specimens and have many seeds, while those of *L.parasitica* are juicy with a translucent pericarp and only have two (to very occasionally four) seeds. Flowers of *L.parasitica* are cosexual while those of *L.oliveriana* are either long- or short-styled. On New Guinea *L.oliveriana* has a wide variety of leaf shapes (Knapp 2022). The Malaku specimens are of plants with broadly elliptic leaves (Fig. 16).

### ﻿﻿7. Lycianthes parasitica

Taxon classificationPlantae

(Blume) Bitter, Abh. Naturwiss. Vereins Bremen 24 [preprint]: 504. 1919.

CDEE34B6-90CC-566A-AAA0-EF54BADAF369

**Figure 18.**
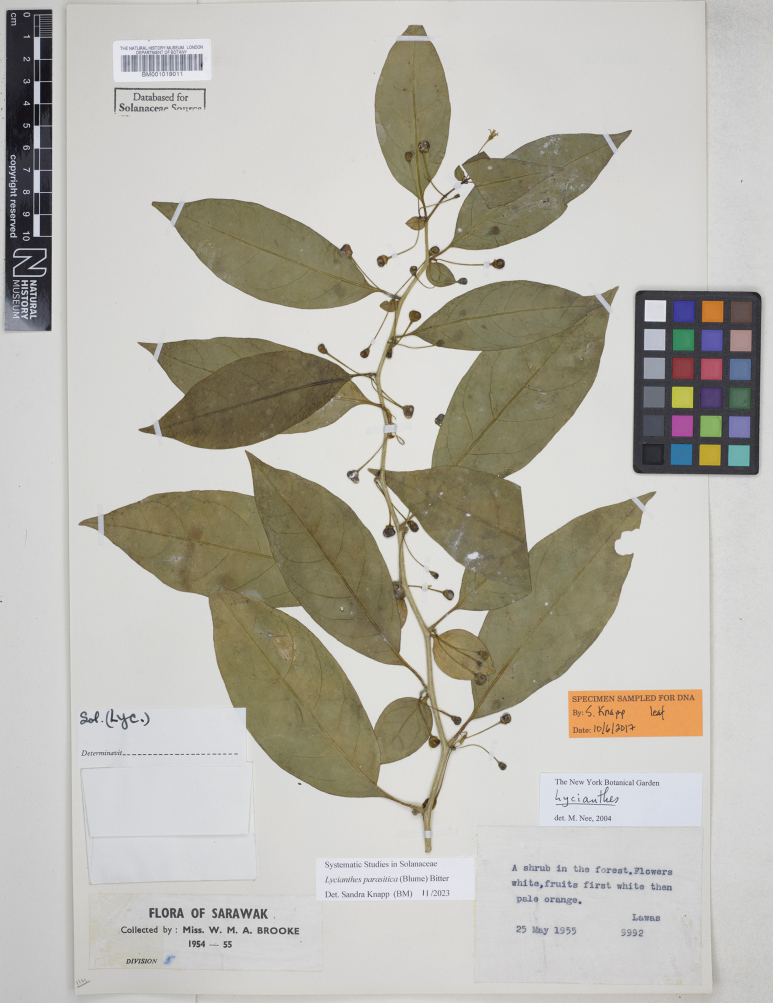
*Lycianthesparasitica* (Elmer) Bitter herbarium specimen. Malaysia. Sarawak, *Brooke 9992* (BM001019011).

Figs 2D , 3C , 4D , 5D, E , 18

Solanum parasiticum Blume, Cat. Gew. Buitenzorg 55. 1823. Type. Indonesia. [Java]: sin. loc. [Blume (1826) cites “montis Salak”], C.L. Blume s.n. (neotype, designated here: L [L 0003603]).
Solanum angatii Elmer, Leaflets Philipp. Bot. 2: 731. 1910, as “Angatii”. Type. Philippines. Mindanao [Davao]: Todaya [Mount Apo], district of Davao, May 1909, *A.D.E. Elmer 10762* (lectotype, designated here: US [00027451, acc. # 779392]; isolectotypes: BISH [BISH1005075], BM [BM001014586], CAL [acc. # 316454], E [E00273867], F [v 0073457F, acc. # 290902], G [G00343323], HBG [HBG-511360], K [K000759398], L [L 003583], LAE [acc. # 229575], LE [LE00016835, LE00016836], MO [MO-503797, acc. # 04871604], NY [00172271], W [acc. # 1912-0001593]).
Solanum epiphyticum Merr., Philipp. J. Sc., C 7: 350. 1912. Type. Philippines. Luzon: sin. loc. [“prov. of Albay” in protologue], 1841, *H. Cuming 837 [as “873*”] (lectotype, designated here: BM [BM001018999); isolectotypes: G [G00415763], K [K000759396, K000759397], LAE [acc. # 229577, 231304]).
Lycianthes parasitica (Blume) Bitter var. campylorhachis Bitter, Abh. Naturwiss. Verein Bremen 24 [preprint]: 505. 1919. Type. Indonesia. Java: [West Java] Gunung Salak, Tjaepoes. 3 Nov 1912, *S.H. Koorders 40378* (lectotype, designated here: BO [acc. # BO-1414436]; isolectotypes: BO [acc. # 1414437, 1911596]).
Lycianthes parasitica (Blume) Bitter var. epiphytica (Merr.) Bitter, Abh. Naturwiss. Verein Bremen 24 [preprint]: 506. 1919. Type. Based on Solanumepiphyticum Merr.
Lycianthes parasitica (Blume) Bitter var. angatii (Elmer) Bitter, Abh. Naturwiss. Verein Bremen 24 [preprint]: 507. 1919, as “Angatii”. Type. Based on Solanumangatii Elmer
Lycianthes parasitica (Blume) Bitter var. praelongipedicellata Bitter, Abh. Naturwiss. Verein Bremen 24 [preprint]: 507. 1919. Type. Indonesia. Sulawesi: [Sulawesi Utara], Tomohon, s.d., *P.B. Sarasin & F.K. Sarasin 375* (holotype: B [destroyed]; no duplicates found).
Lycianthes parasitica (Blume) Bitter var. plurifolia Bitter, Repert. Spec. Nov. Regni Veg. 18: 321. 1922. Type. Philippines. Visayas: Eastern Visayas, Leyte Island, 19 Sep 1919, *C.A. Wenzel 500* (holotype: G [G00415812]; isotypes: BM [BM001018987], F [acc. # 423585], MO [acc. # 80818, 80819]).

#### Type.

Based on *Solanumparasiticum* Blume

#### Description.

Epiphytic shrubs, 1–2 m tall, to 20 m high in trees; stems terete, glabrescent, occasionally (in eastern Sabah, see discussion) sparsely pubescent with weak-walled transparent simple uniseriate 2–10-celled trichomes when young, often markedly zig-zag; new growth glabrous or occasionally pubescent with transparent, simple uniseriate 8–10-celled trichomes to 0.2–1 mm long; bark of older stems glabrous or glabrescent, papery-white, markedly exfoliating. Sympodial units difoliate, the leaves geminate, the leaves of a pair differing in size and occasionally in shape, the minor leaves often apparently absent. Leaves simple; blades of major leaves 7–21 cm long, 2–8.4 cm wide, narrowly elliptic to elliptic, widest in the middle, concolorous and shiny, membranous or somewhat fleshy; adaxial surfaces glabrous or occasionally sparsely pubescent with simple uniseriate trichomes like those of the stems, these denser along the veins; abaxial surfaces glabrous to sparsely pubescent with simple uniseriate trichomes like those of the stems, if the lamina glabrous the trichomes confined to the principal veins and midrib; principal veins 5–6 pairs, glabrous or pubescent, the tertiary venation not visible; base acute to attenuate; margins entire; apex acute to acuminate; petiole 0.7–2 cm long, fleshy, glabrous or sparsely pubescent with simple uniseriate trichomes like those of the new growth; blades of minor leaves 1–3 cm long, 1–2.5 cm wide, elliptic to orbicular; surfaces like those of the major leaves, the minor leaves often deciduous; principal veins of minor leaves 3–4 pairs; base acute to rounded-truncate; margins entire; apex obtuse or somewhat rounded; petiole of minor leaves 0.5–0.7 cm long, glabrous or sparsely pubescent. Inflorescences axillary, in fascicles or on a short rhachis to 0.5 cm long, with 3–8 (16) flowers, glabrous; pedicels at anthesis 1–1.5 cm long, ca. 0.5 mm in diameter at the base, ca. 1 mm in diameter at the apex, spreading, glabrous and shiny or occasionally pubescent with simple uniseriate trichomes like those of the stems, articulated at the base; pedicel scars tightly packed and overlapping on the short rhachis, somewhat corky. Buds ellipsoid, the corolla completely included in the calyx tube when young, ca. halfway exserted before anthesis, the calyx appendages only apparent in bud. Flowers 5-merous, cosexual. Calyx tube 1.5–2.5 mm long, 2.5–3.5 mm wide at the mouth, obconical, glabrous or occasionally pubescent with simple uniseriate trichomes like those of the pedicels, with 5 small appendages arising ca. 0.5 mm below the hyaline rim and only visible in bud, the appendages ca. 0.2 mm long, triangular nubs, usually perpendicular to the calyx tube, glabrous or with a few trichomes. Corolla 0.9–1 cm in diameter, white or pale violet (lilac), stellate, lobed nearly to the base, interpetalar tissue absent but a thin edge of tissue apparent on lobe margins, the lobes 4–4.5 mm long, 1.2–2 mm wide, spreading, glabrous on both surfaces, the tips and margins densely papillate, the tips cucullate. Stamens equal; filament tube minute; free portion of the filaments ca. 1 mm long, glabrous; anthers 2–2.3 mm long, 1–1.5 mm wide, ellipsoid and somewhat tapering, yellow, glabrous, poricidal at the tips, the pores tear-drop shaped and edged with white in dry material, lengthening to slits with age. Ovary conical, glabrous; style 4.5–5 mm long, exserted from the anther cone, glabrous; stigma prominently capitate, the surfaces minutely papillate. Fruit a globose berry, 0.5–0.8 cm in diameter, green when immature, becoming white, then orange or red when mature, the pericarp glabrous, thin, shiny, and transparent; fruiting pedicels 1.5–2.2 cm long, 0.5–1 mm in diameter at the base, ca. 2 mm in diameter at the apex, spreading; fruiting calyx not accrescent or expanding, but remaining a cup beneath the berry, 2–2.5 mm long, 3–4 mm wide at the mouth. Seeds 2(-4) per berry, 3.5–6 mm long, ca. 2.5–4.5 mm wide, ovoid-reniform, pale straw-colored, the surfaces deeply pitted, the testal cells sinuate in outline and with prominent “hairy” appendages on the thickened lateral walls. Stone cells absent. Chromosome number not known.

#### Distribution

**Figure 19.**
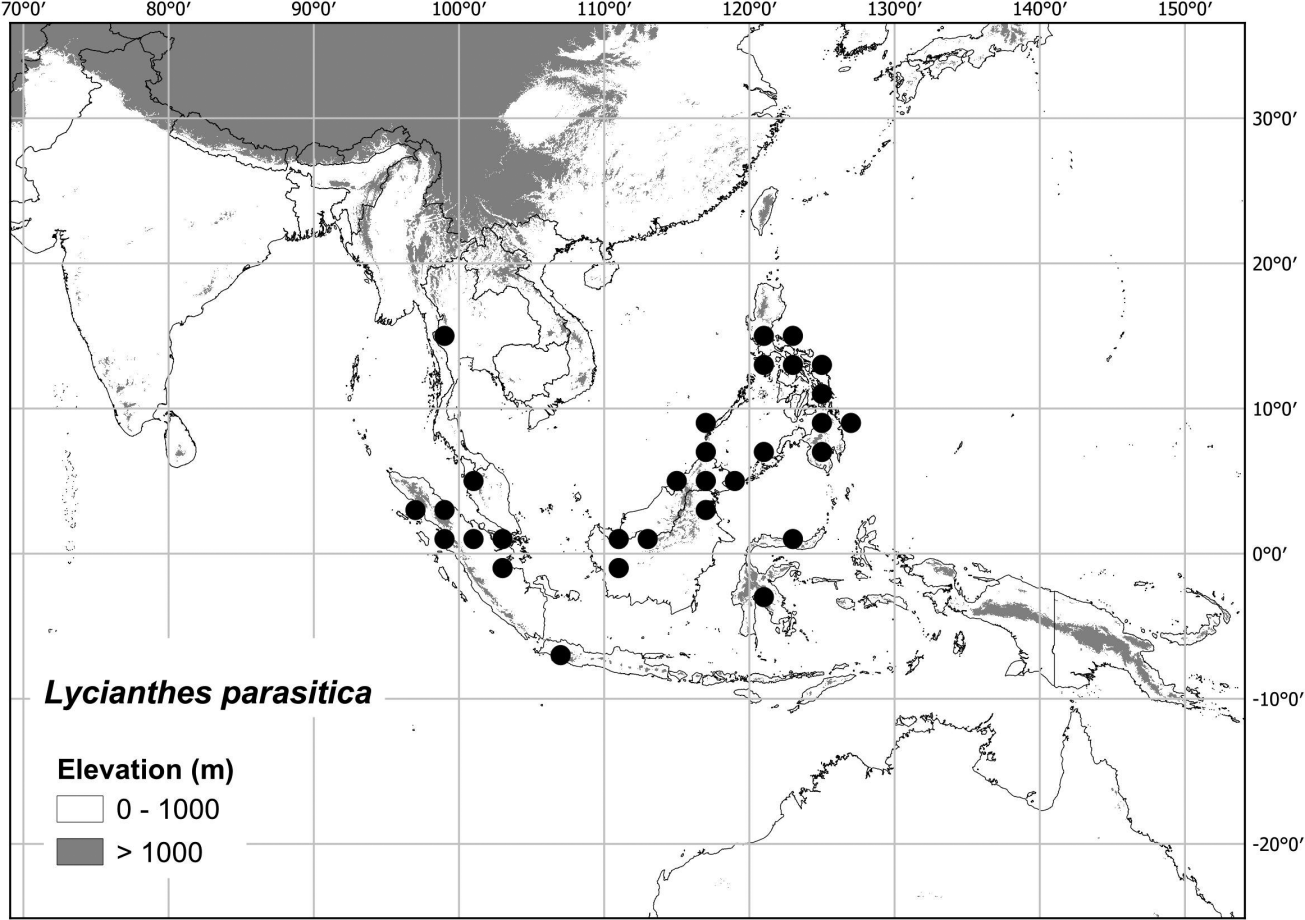
Distribution of *Lycianthesparasitica*.

**(Fig. 19).***Lycianthesparasitica* occurs in Brunei Darussalam, Indonesia, Malaysia, the Philippines and Thailand (a single sterile specimen only, *Put Phraisurind 122*).

#### Ecology and habitat.

*Lycianthesparasitica* grows as an epiphytic shrub in wet forests, usually at low elevations along rivers and streams and in swamp forests, from 100 to 1,000 m elevation.

#### Common names.

Brunei Darussalam: usak oncom payo (Dusun language, *Bernstein 322*). Indonesia. Java: terong-an (Blume 1823); Sumatra: sarindon (*si Boeea 6246*, *9081*). Malaysia. Sabah: akar (Bakar 25023), akar tumpeng (Sungei Kinabatangan language, *Enggoh 7345*). Philippines. Mindanao: lawmoos (Bagóbo language, Elmer 1910), sisumbidan (Manobo language, *Elmer 13572*).

#### Preliminary conservation assessment

**(IUCN 2020).**EOO (26,990,176 km^2^ - LC); AOO (1,684 km^2^ - VU). *Lycianthesparasitica* is a species known from more than five localities and is relatively widely distributed in the region, though as an epiphyte is rarely collected. Throughout the range it is known from protected areas (e.g., Mount Kinabalu and the Sepilok Forest Reserve, Sabah, Malaysia; Sohoton National Park on Samar Island in the Philippines). The assessment of Vulnerable (VU) based on AOO is likely due to collecting bias, but also due to the island nature of the distribution. I therefore assign it a preliminary status of Least Concern (LC).

#### Discussion.

*Lycianthesparasitica* is distinctive in its epiphytic habit, usually strongly zig-zagging stems with markedly exfoliating white bark and berries with few (usually only two), large seeds (Figs 2D, 5E). The epiphytic habit has evolved independently several times in the Solanaceae (Orejuela 2021). The leaves are shiny on the adaxial surfaces and quite fleshy, in dried specimens the lower orders of venation are barely visible. In flower it is possible that *L.parasitica* could be confused with *L.laevis*, which similarly has 5 calyx appendages, but that species is a shrub, not an epiphyte, has larger flowers and berries with more than 20 rather than only 2 or 3 seeds. The calyx appendages of *L.parasitica* are usually not prominent in flower (Figs 3C, 4D). The berries of *L.parasitica* change from green to white, then orange or bright red during maturation (Fig. 5D), whereas those of *L.laevis* turn only from green to orange or red (Fig. 2B).

The minor leaves of the geminate pair are often deciduous in older stems of *L.parasitica*; this was the principal feature used by Merrill (1912) in distinguishing his new species *S.epiphyticum*.

In the Sandakan area of Sabah (Malaysian Borneo) several collections of *L.parasitica* (e.g., *Evangelista 883*, *Pereira & Postar SAN-151226* and *Sinclair 9347*) are distinctive in being densely pubescent with simple uniseriate trichomes on all parts. These collections correspond to the glabrous individuals of *L.parasitica* in flower and leaf size and in numbers of seeds in berries, so I have retained them here, recognising that pubescence in *Lycianthes* in Asia is extremely variable. Further investigation of these populations would be of interest.

*Solanumparasiticum* was described in a footnote to a species list of plants found near the botanic gardens in Bogor (Blume 1823) without citation of specimens, nor were specimens cited in his later treatment (Blume 1826). No specimens collected by Blume are extant in the Bogor herbarium (A. Kartonegoro, pers. comm.). When he left Indonesia for the Netherlands Blume took with him several cases of specimens he and others had collected (van Steenis 1989). I have selected a neotype from among the specimens at Leiden (*Blume s.n*., L 0003603) corresponding to the description in the protologue.

Like other species described by Elmer (1910), no herbarium was cited in the protologue of *S.angatii*. I have selected the best preserved of the duplicates of the single collection cited (*Elmer 10762*) as the lectotype. This species was named in honour of Elmer’s Bagóbo guide Angat from the village of Todaya, for whom he had great praise (Elmer 1910).

Merrill (1912) described *S.epiphyticum* from several collections, citing one of them (“*Cuming 873*”) as “type”, but with no herbarium mentioned. There are no collections of *Cuming 873* that are Solanaceae, but *Cuming 837* is from the type locality, and I am considering Merrill’s (1912) citation of “*Cuming 837*” as an error to be corrected. Since the herbarium in Manila was burnt during the Second World War and all specimens held there were destroyed (see discussion under *L.banahaensis*), I have selected the sheet at BM (BM001018999) as the lectotype; it has well-preserved material with both flowers and fruits.

Bitter (1919) synonymised *S.angatii* and *S.epiphyticum* under his concept of *L.parastica*, and described two additional infraspecific taxa, vars. *campylorhachis* and *praelongipedicellata*. He cited a single specimen from Bogor (“Koorders 40378β ! (hb. Bogor)”) for var. campylorhachis; I have selected the best preserved and most complete of the three duplicates of this collection (BO acc. # BO-1414436) as the lectotype. A collection made by the cousins Karl F. and Paul B. Sarasin (“it. celeb. Sarasinorum n. 375!”) from Berlin was cited by Bitter (1919) for var. praelongipedicellatum; Berlin specimens were destroyed and most of this material is lost (van Steenis Kruseman 1985). I have seen no duplicates of any Sarasin collections. A neotype should be sought from the area where the type was gathered (https://openlibrary.org/books/OL16289390M/Reisen_in_Celebes#overview), I have seen no specimens of *L.parasitica* from Sulawesi.

Bitter (1922) later described L.parasiticavar.plurifolia, citing a single specimen in “herb. Delessert”, held in G; in the absence of any evidence that he could have seen others of this collection (*Wenzel 500*), I suggest the specimen at G (G00415812) is the holotype.

### ﻿﻿8. Lycianthes rantonnetii

Taxon classificationPlantae

(Carrière) Bitter, Abh. Naturwiss. Vereins Bremen 24 [preprint]: 332. 1919.

C6EFD43A-E9B3-521F-8F0E-6897BC409E41

Figs 4E , 20

Solanum rantonnetii Carrière, Rev. Hort. [Paris] 32: 135. 1859, as “rantonnei”. Type. Cultivated in Paris (lectotype, designated by Dean et al. 2020, pg. 180: [illustration] Carrière, Rev. Hort. [Paris] 32: fig. 32. 1859).
Solanum corniculatum Hiern, Vidensk. Meddel. Naturhist. Foren. Kjobenhavn 1877–1878: 45. 1877, nom. illeg., not S.corniculatum Huber (1865). Type. Brazil. Rio de Janeiro: sin. loc., 1867, *A. Glaziou 1078* (lectotype, designated by Dean et al. 2020, pg. 180: C [C10019192]; isolectotypes: BR [BR00000552267, BR00000552234], P [P00325613, P00325614, P00430738]).
Solanum urbanum Morong, Ann. New York Acad. Sci. 7: 177. 1893. Type. Paraguay. Central: streets of Asunción, Nov 1888, *T. Morong 147* (lectotype, designated by Barboza 2013, pg. 29: NY [00172225]; isolectotypes: MO [MO-503602, acc. 2495263], NDG [NDG45160], PH [00030498], US [0027939, acc. # 1324871], WIS [v0004256WIS]).
Solanum muticum N.E.Br., Bull. Misc. Inform. Kew 85: 6. 1894. Type. Uruguay. Montevideo: cultivated in Montevideo, originally from Paraguay, Mar 1858, *E.J. Gibert 56* (lectotype, designated by Barboza 2013, pg. 29: K [K000585755]).
Solanum urbanum Morong var. foliosum Chodat, Bull. Soc. Bot. Genève, ser. 2, 8: 152. 1916. Type. Paraguay. Paraguarí: Paraguary, Cerros de Paraguarí, Sep 1914, *R. Chodat & W. Vischer 60* (lectotype, designated by Knapp 2022, pg. 93: G [G00392293]).
Solanum urbanum Morong var. nervosum Chodat, Bull. Soc. Bot. Genève, ser. 2, 8: 152. 1916. Type. Paraguay. Paraguay. Cordillera: “in valle fluminis Y-acá, pr[ope] Valenzuela”, Jan 1900, *É. Hassler 7024* (lectotype, designated by Dean et al. 2020, pg. 180: G [G00390048]; isolectotypes: BM [BM000087583], G [G00392285, G00392288, G00392290], P [P03852955], W [acc. # 1904-804]).
Solanum urbanum Morong var. subtomentosum Chodat, Bull. Soc. Bot. Genève, ser. 2, 8: 152. 1916. Type. Paraguay. Misiones: San Ignacio, Oct 1914, *R. Chodat & W. Vischer 61* (lectotype, designated by Knapp 2022, pg. 93: G [G00392295]).

#### Type.

Based on *Solanumrantonnetii* Carrière

#### Description.

Shrubs 0.5–3 m tall, with multiple stems from the base, these arching and sometimes scandent and sprawling; stems 3–4-angled, the angles yellowish green in live plants and paler than the rest of the stem, sparsely to moderately pubescent with spreading transparent simple uniseriate 1–4-celled trichomes to 0.5 mm long, these occasionally forked or dendritic, glabrescent with age; new growth moderately pubescent with transparent simple uniseriate or occasionally dendritic trichomes like those of the stems; bark of older stems pale greyish brown, prominently angled. Sympodial units unifoliate or more usually difoliate, the leaves usually geminate, if paired the leaves similar in shape and size. Leaves simple; blades of major leaves (1)4–15.5 cm long, (0.5)3.5–7.5 cm wide, ovate, rhombic-elliptic, elliptic or occasionally almost lanceolate, broadest in the upper half or rarely at the middle, membranous, concolorous; adaxial surfaces sparsely and evenly pubescent with 1–3-celled simple uniseriate trichomes, these denser along the midrib; abaxial surfaces sparsely to moderately and evenly pubescent with 1–3-celled simple uniseriate trichomes, these denser along the midrib; principal veins 3–7 pairs, more pubescent than the lamina. drying yellowish green abaxially; base attenuate onto the petiole; margins entire or somewhat undulate; apex acute to acuminate; petiole 0.5–2.4(4) cm long, winged from the attenuate leaf base, pubescent with simple uniseriate (or occasionally dendritic) trichomes like those of the stems and leaves; blades of minor leaves similar in size and shape to those of the major leaves, or slightly smaller; petioles 0.5–3 cm long, winged. Inflorescences axillary fascicles with (1)2–7 flowers, pubescent with transparent trichomes like those of the new growth and stems; pedicels 1.2–1.7 cm long, ca. 1 mm in diameter at the base, ca. 2 mm in diameter at the apex, spreading at anthesis, sparsely to moderately and evenly pubescent with transparent simple (occasionally dendritic) uniseriate 1–3-celled trichomes like those of the stems, articulated at the base; pedicels scars tightly packed in the leaf axils. Buds ellipsoid to fusiform with pointed tips, the corolla more than halfway exserted from the calyx tube before anthesis. Flowers 5-merous, all apparently cosexual. Calyx with the tube 1.5–4 mm long, 2.5–4.5 mm wide, openly cup-shaped, with (5)10 linear subulate appendages of variable length 0.25–5.2 mm long, arising ca. 0.25–1 mm from the hyaline rim, usually alternating long and short, sparsely to moderately pubescent with simple trichomes like those of the pedicels. Corolla 1.2–2 cm in diameter, violet with the midveins dark purple and the centre yellow, rotate, lobed less than 1/10 of the way to the base, interpetalar tissue abundantly present, the lobes ca. 1 mm long, ca. 1 mm wide and mere acumens from the rotate corolla, glabrous on both surfaces except for the densely papillate, cucullate tips (acumens). Stamens unequal; filament tube minute; free portion of the filaments of two lengths, three long filaments 2–3 mm long, two short filaments 0.8–1.5 mm long, glabrous or adaxially pubescent with tangled weak-walled uniseriate simple trichomes; anthers ellipsoid and slightly curved, orange-yellow, glabrous, poricidal at the tips, the pores round, distally directed, not elongating to slits with age. Ovary conical, glabrous; style 3.5–5.5 mm long, slightly curved in the same direction as the anthers, glabrous; stigma slightly clavate and bilobed, the surface minutely papillate. Fruit a compressed-ellipsoid or compressed globose berry, 2–3 cm long, 1.3–1.5 cm in diameter (usually absent or smaller and seedless in cultivated plants), yellow or yellowish orange when mature, the pericarp glabrous, thin, shiny and translucent; fruiting pedicels 2.5–4 cm long, ca. 1.5 mm in diameter at the base, ca. 3 mm in diameter at the apex, somewhat woody, spreading or hanging from the weight of the berries; fruiting calyx a plate with the appendages somewhat longer than in flower, spreading and often broken off, stiff and woody. Seeds 20–100 per berry (many fewer in cultivated plants), 2–3.5 mm long, 1.5–3.5 mm wide, rounded and compressed, reddish tan, the surfaces minutely pitted, the testal cells with sinuate margins, “hairy” extensions of lateral testal cell walls absent. Stone cells more than 20 per berry, ca. 0.5–1.5 mm in diameter. Chromosome number: 2n = 24 (Gerasimenko and Reznikova 1968 [cited in D’Arcy 1974] as *Solanumrantonnetii*; Acosta et al. 2005, as *L.rantonnei*, voucher *Moscone 4260* [CORD]).

#### Distribution.

*Lycianthesrantonnetii* is widely cultivated in the tropics and subtropics (and even into the temperate zone as a short-lived perennial) worldwide. In this region I have only seen specimens from India and Pakistan. It is native to southern South America (Argentina, Bolivia, southern Brazil and Paraguay).

#### Ecology and habitat.

In its native range *L.rantonnetii* is a plant of semi-moist, seasonal forests and open areas; from (sea level) 100 to 2,000 m elevation.

#### Common names.

In its native range in Argentina *L.rantonnetii* is called meloncillo del aire (Barboza 2013).

#### Preliminary conservation assessment

**(IUCN 2020).** Not applicable to this species for this region.

#### Discussion.

*Lycianthesrantonnetii* is native to South America (Barboza 2013) but widely cultivated in subtropical and temperate areas worldwide. It is the only species occurring in the region treated here that has stone cells in the berries, but in cultivation it rarely sets fruit. It can easily be distinguished from all native species by its rotate corollas with copious interpetalar tissue (Fig. 4E), orange-yellow anthers that are slightly curved and angled, and somewhat striped stems. *Lycianthesrantonnetii* has variable length calyx appendages like *L.schizocalyx*, but the two species are not easy to confuse; *L.schizocalyx* has small bright red berries without stone cells whereas *L.rantonnetii* rarely sets fruit in cultivation, but, when it does, the berries are dirty yellow and have copious stone cells. In Asia I have only seen specimens of *L.rantonnetii* from India and Pakistan, but I would expect it to be in cultivation anywhere in subtropical areas of Asia.

**Figure 20.**
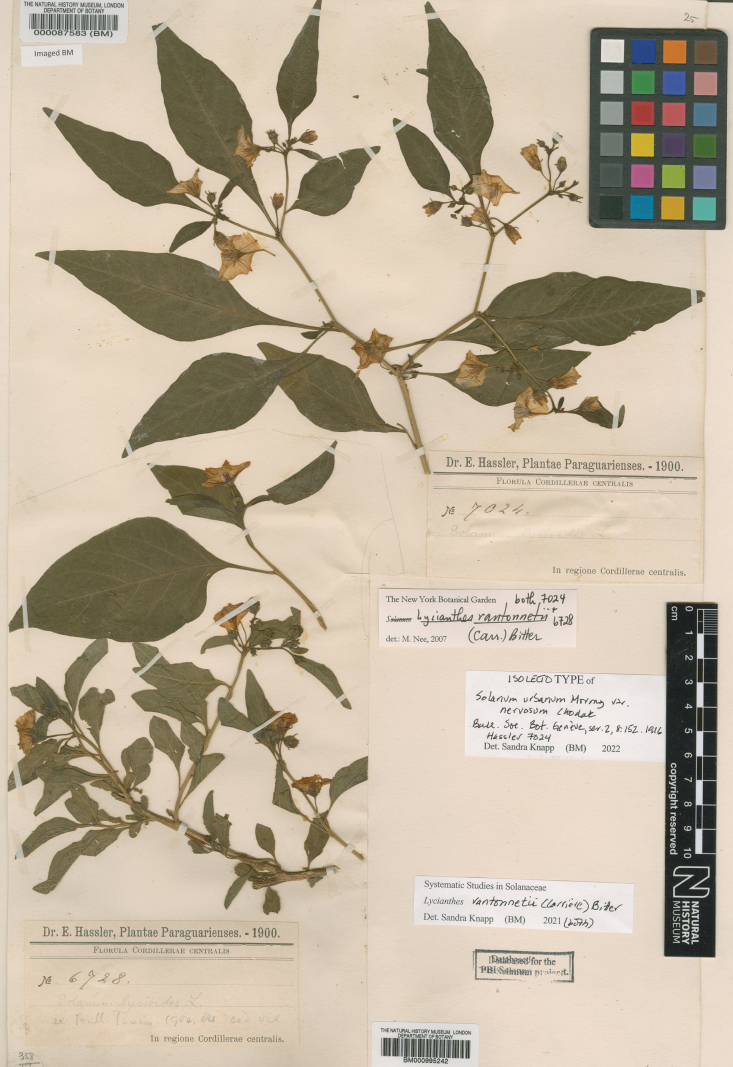
*Lycianthesrantonnetii* (Carrière) Bitter herbarium specimen. Paraguay. Cordillera: *Hassler 7024* (isolectotype of S.urbanumvar.nervosum Chodat, BM000087583) and *Hassler 6728* (BM000995242). Courtesy of the Trustees of the Natural History Museum, London, reproduced with permission.

The specific epithet is often seen spelled “rantonnei” but is correctable to “rantonnetii” following Art. 60.9 of the ICN (Turland et al. 2018: Ex. 31), which stipulates that epithets honouring persons where there is an intentional latinisation of the name that involves the omission of a terminal vowel or consonant are not permitted; the epithet in this case honours the French horticulturalist Barthélémy Victor Rantonnet so is correctable to “rantonnetii” even though Carrière (1859) originally spelled it “rantonnei” (using the latinisation Rantonneus).

### ﻿﻿9. Lycianthes schizocalyx

Taxon classificationPlantae

(Merr.) Bitter, Abh. Naturwiss. Vereins Bremen 24 [preprint]: 461. 1919.

5C8E3D70-5739-5B82-BA84-864F3B319C1C

Figs 3E , 21

Solanum biflorum Lour. var. corynephorum Kuntze, Revis. Gen. Pl. 2: 453. 1891. Type. Indonesia. Java: “Java: Tjibodas, 4,600f”, 25 May 1875, *O. Kuntze 4574* (lectotype, designated here: NY [00172276]; isolectotype: NY [00172275)).
Solanum schizocalyx Merr., Philipp. J. Sci. 5(C): 383. 1910. Type. Philippines. Luzon [Cordillera CAR]: Mount Data, District of Lepanto, Nov 1905, *E.D. Merrill 4548* (no herbaria cited; lectotype, designated here: US [00027886, acc. # 71033]; isolectotypes: K [K000759387], L [L.2859466], NY [00138723], P [P00368998]).
Lycianthes brachyanthera Bitter, Abh. Naturwiss. Vereins Bremen 24 [preprint]: 473. 1919. Type. Indonesia. Sulawesi: [Sulawesi Utara], Lokon [Gunung Lokon, near Tomohon], s.d., *P.B. Sarasin & F.K. Sarasin [VI 44a] 396* (holotype: B [destroyed]; no duplicates found, in synonymy ex descr.).
Lycianthes minutipila Bitter, Abh. Naturwiss. Vereins Bremen 24 [preprint]: 473. 1919. Type. Indonesia. Sumatra: [Aceh], Boernipaja [protologue “Gajoe Loeas in Alas Landen”], 16 Feb 1904, *G.C.E. van Daalen & R.M. Pringgo Atmodjo 21* [cited only as van Daalen by Bitter] (lectotype, designated here: BO acc. # BO-1324388]; isolectotype: BO [acc. # BO-1414432]).
Lycianthes denticulata (Blume) Bitter var. liophylla Bitter, Abh. Naturwiss. Vereins Bremen 24 [preprint]: 474. 1919. Type. Myanmar (Burma). “Tenasserim, Molyet” [= Tanintharyi Region], 1,750 m, *G. Gallatly 189* (holotype: B [destroyed]; lectotype, designated here: CAL [acc. # 315676]; isolectotype: CAL [no acc. #]).
Lycianthes laevis (Dunal) Bitter var. glabratula Bitter, Abh. Naturwiss. Vereins Bremen 24 [preprint]: 488. 1919. Type. Philippines. Luzon [Cordillera CAR]: Pauai, prov. of Benguet, 2,100 m, Jun 1910, *R.C. McGregor 8393* (lectotype, designated here: US [02840845, acc. # 628912]; isolectotype: B, [destroyed]).
Lycianthes laevis (Dunal) Bitter subsp. luzonensis Bitter, Abh. Naturwiss. Vereins Bremen 24 [preprint]: 489. 1919. Type. Philippines. Luzon [Cordillera CAR]: Mount Santo Tomas [“Mount Tonglon, prov. Benguet”], Aug 1906, *H.M. Curran 5029* (lectotype, designated here: US [00027887, acc. # 708756]; isolectotypes: B [destroyed], CAL [no acc. #]).
Lycianthes laevis (Dunal) Bitter var. majuscula Bitter, Abh. Naturwiss. Vereins Bremen 24 [preprint]: 488. 1919. Type. Indonesia. Java: “West-Java. Berg Malabar”, *E.M. Wichura 2168* (holotype: B [destroyed]; no duplicates found, in synonymy ex descr.).
Lycianthes baviensis V.V.Hop, J. Biol. (Vietnam) 26(4A): 44. 2004. Type. Vietnam. Ha Noi: Hà Tây, Mt. Ba Vi, 800–1,200 m, 24 Apr 1976, *Nguyen Van Phu HPP 136* (lectotype, designated here: HN [sheet with label saying “Typus” in V.V. Hop hand”]; isotypes: HN [two sheets, not with annotation as “typus”]).

#### Type.

Based on *Solanumschizocalyx* Merr.

#### Description.

Shrubs, 0.75–2 m tall; stems terete, glabrous or variously pubescent with golden or whitish grey translucent simple uniseriate trichomes to 1.5 mm long, these appressed or spreading and tangled, older stems glabrescent; new growth glabrous to sparsely or densely pubescent with translucent, simple uniseriate 2–10-celled trichomes to 1.5 mm long; bark of older stems glabrescent, greyish brown or dark brown. Sympodial units difoliate, the leaves geminate, the leaves of a pair differing in size and occasionally in shape, the minor leaves if different in shape usually more ovate. Leaves simple; blades of major leaves 8–20 cm long, 2.5–6.5 cm wide, elliptic to narrowly elliptic, widest in the middle or just below, concolorous but occasionally somewhat discolorous, membranous; adaxial surfaces glabrous to evenly and sparsely to moderately pubescent with simple uniseriate trichomes like those of the stems, these denser along the veins, the lamina always visible; abaxial surfaces glabrous to evenly and moderately pubescent with simple uniseriate trichomes like those of the stems, the lamina clearly visible, the trichomes denser on the principal veins and midrib; principal veins 7–8 pairs, usually sparsely to moderately pubescent, occasionally glabrous. the venation not markedly prominent; base attenuate; margins entire, in pubescent plants ciliate with simple uniseriate trichomes to 1 mm long; apex acute to acuminate; petiole 1–3.5 cm long, glabrous or pubescent with simple uniseriate trichomes like those of the stems and new growth; blades of minor leaves 3–5 cm long, 1–2 cm wide, elliptic to narrowly elliptic or ovate, sometimes almost orbicular; surfaces like those of the major leaves; principal veins of minor leaves 5–6 pairs; base attenuate; margins entire; apex acute to acuminate; petiole of minor leaves 0.5–1.2 cm long, glabrous or sparsely pubescent like the stems. Inflorescences axillary, in fascicles, with 1–6 flowers, glabrous or with a few trichomes at the pedicel bases; pedicels at anthesis (1)2–3 cm long, ca. 1 mm in diameter at the base, 1.5–2.5 mm in diameter at the apex, deflexed (perhaps spreading?), glabrous to pubescent with simple uniseriate trichomes like those of the stems, articulated at the base; pedicel scars tightly packed in the leaf axils. Buds strongly tapered and pointed, the corolla strongly exserted from the calyx tube before anthesis, the calyx appendages enclosing the bud only partially. Flowers 5-merous, cosexual. Calyx tube 4–5 mm long, 4–5.5 mm wide at the mouth, an open cuplike structure, strongly 7–10-ridged, sometimes coloured purple or dull violet (fide *Jacobs 7367*), glabrous or pubescent with simple uniseriate trichomes like those of the pedicels, the trichomes often denser on the ridges, with 7–10 appendages arising at and ca. 0.5 mm below the hyaline rim, the appendages 1.5–5 mm long, ca, 0.5 mm wide, differing in length in the same flower, subulate, parallel to the calyx tube, glabrous or pubescent with simple uniseriate trichomes like those of the stems and pedicels. Corolla 2–3 cm in diameter, white, violet or purple, occasionally with a darker purple centre (fide *Jacobs 7367*), stellate, lobed 3/4 or nearly to the base, interpetalar tissue mostly absent but a thin edge of tissue apparent on lobe margins, the lobes 9–13 mm long, 3–5.5 mm wide, spreading, glabrous on both surfaces, the tips cucullate and densely papillate. Stamens equal; filament tube minute; free portion of the filaments 1–1.5 mm long, glabrous; anthers 4–5 mm long, ca. 2 mm wide, ellipsoid and tapering to a beak-like apex, tightly connivent, yellow, glabrous, poricidal at the tips, the pores tear-drop shaped and edged with white in dry material, lengthening to slits with age. Ovary conical, glabrous; style 9–11 mm long, exserted from the anther cone, glabrous; stigma prominently capitate, the surfaces minutely papillate. Fruit a globose berry, 1–1.3 cm in diameter, green when immature, purple or bright red when mature, the pericarp glabrous, thin, shiny, transparent at fruit maturity; fruiting pedicels 2–3 cm long, ca.1 mm in diameter at the base, ca. 2 mm in diameter at the apex, spreading; fruiting calyx not accrescent or expanding, but remaining a cup covering ca. 1/4 of the berry, the appendages spreading like a star beneath the berry. Seeds 40–60 per berry, 3.5–4 mm long, ca. 2.5 mm wide, flattened-reniform, pale straw-coloured or golden tan, the surfaces pitted, the testal cells sinuate in outline in the centre, rectangular on the margins, prominent “hairy” appendages absent. Stone cells absent. Chromosome number not known.

**Figure 21.**
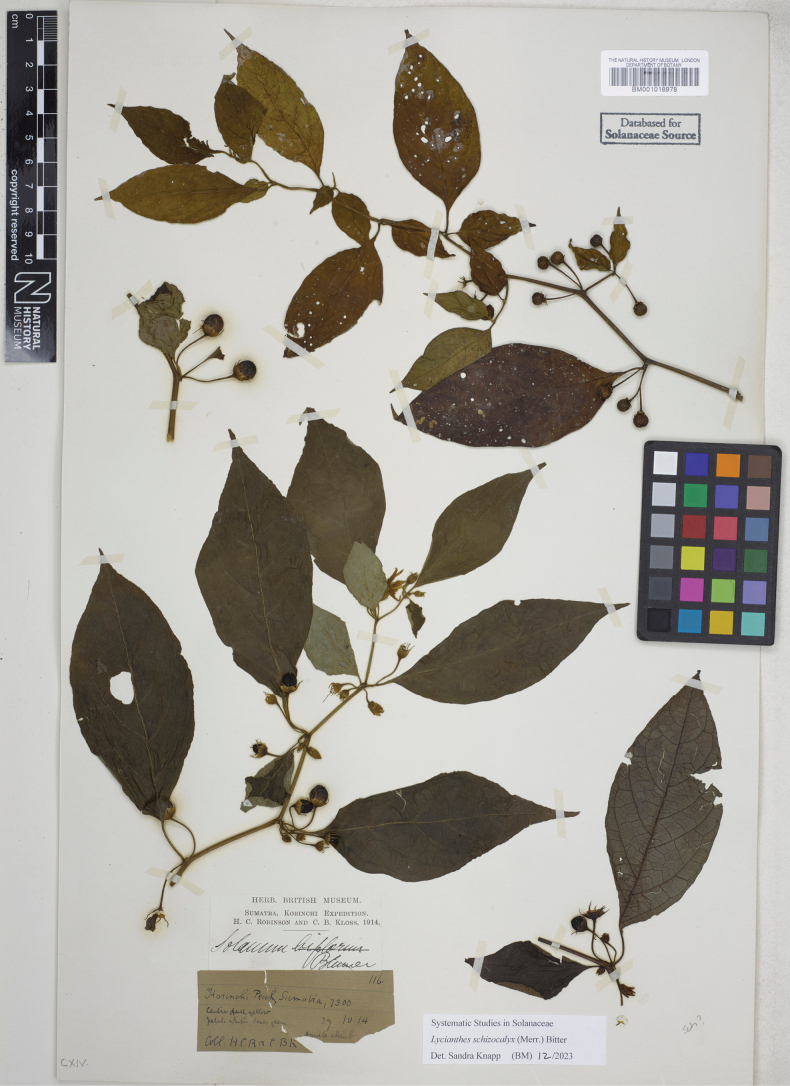
*Lycianthesschizocalyx* (Merr.) Bitter herbarium specimen. Indonesia. Sumatra, *Robinson & Kloss 116* (BM001018978). Courtesy of the Trustees of the Natural History Museum, London, reproduced with permission.

#### Distribution

**Figure 22.**
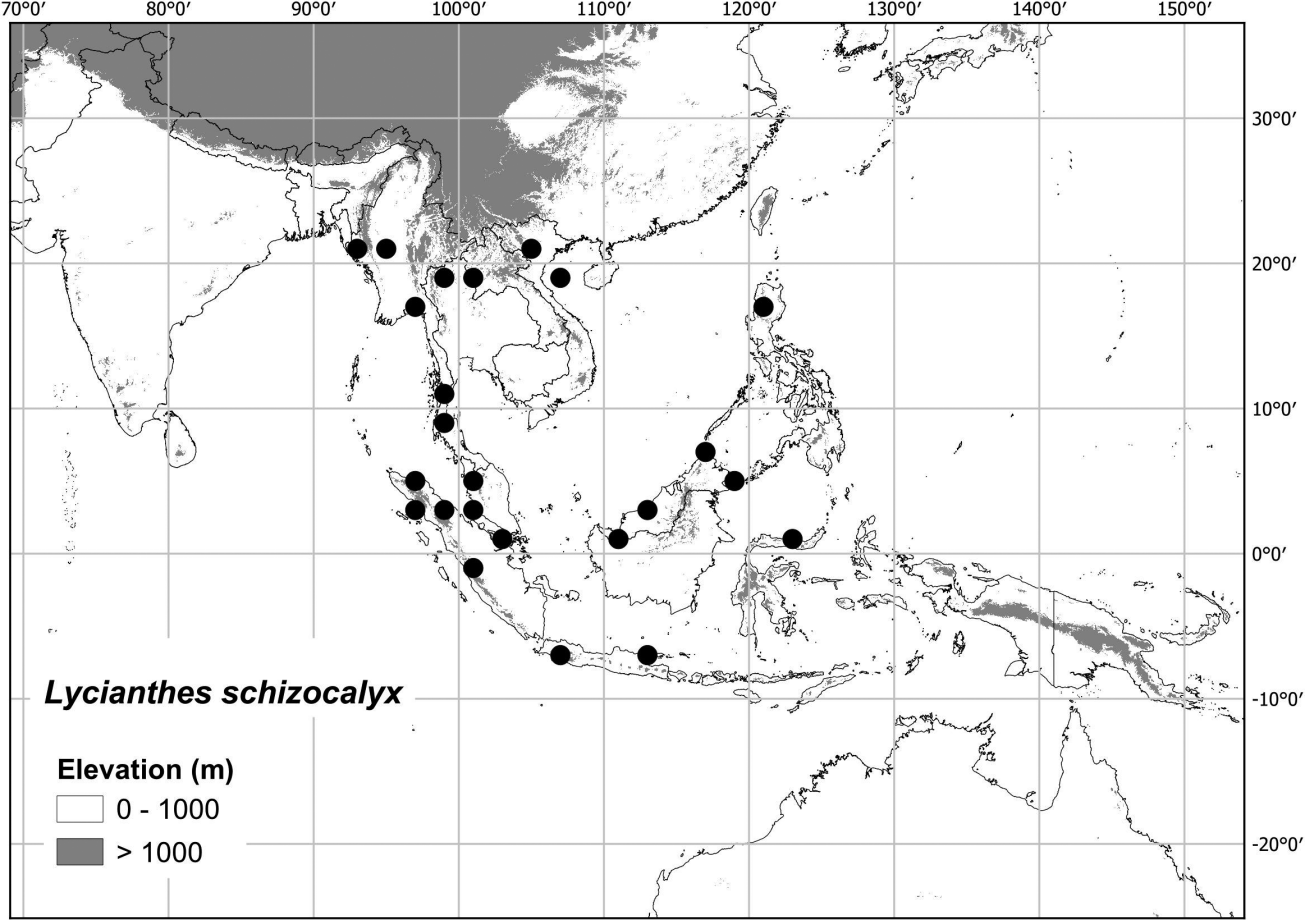
Distribution of *Lycianthesschizocalyx*.

**(Fig. 22).***Lycianthesschizocalyx* occurs in India, Indonesia, Malaysia, Myanmar (Burma), Philippines, Thailand and Vietnam.

#### Ecology and habitat.

*Lycianthesschizocalyx* is found in montane forests, evergreen forests and disturbed areas in these forests, usually growing in the understory in wet areas, from (100–)1,000 to 2,500 m elevation. Most collections are from above 1,000 m elevation.

#### Common names.

Vietnam. cà ngủ ba vì (Hop 2017).

#### Preliminary conservation assessment

**(IUCN 2020).**EOO (7,731,851 km^2^ - LC); AOO (368 km^2^ - EN). *Lycianthesschizocalyx* is known from more than ten localities and is quite widely distributed in the region. Throughout the range it is known from protected areas (e.g., Mount Data National Park in the Philippines, Mount Kinabalu in Sabah, Malaysia). The assessment of Endangered (EN) based on AOO is likely due to collecting bias, but also due to the island nature of the distribution. I therefore assign it a preliminary status of Least Concern (LC).

#### Discussion.

*Lycianthesschizocalyx* is one of several Asian species of *Lycianthes* with more than 5 (usually 10) calyx appendages. It is most similar to *L.biflora* in its shrubby habit, the usually sublate or linear calyx appendages, flowers held below the leaves maturing to erect fruits, and in its elliptic, ovate or narrowly elliptic leaves. Merrill (Merrill and Merritt 1910) suggested *L.schizocalyx* was “manifestly allied” to *L.biflora* and distinguished it by “being nearly glabrous, with comparatively large flowers and its calyx splitting down one side.” Pubescence varies throughout the range of *L.schizocalyx*, as it does in most of these species, and the splitting of the calyx tube occurs often in *L.schizocalyx* but is not unique to the species.

*Lycianthesschizocalyx* can be distinguished from *L.biflora* – sometimes with difficulty – by its usually fewer-flowered fascicles, calyx appendages that differ in length within a flower and some of which arise from below the calyx tube rim (Fig. 3E), the usually strongly ribbed calyx tube (although this can be the case in *L.biflora* as well) and flowering pedicel length [(1)2–3 cm long in *L.schizocalyx* versus 0.9–1.2 cm long in *L.biflora*]. The flowering pedicel length is the most consistent character separating the two taxa, along with the insertion of the calyx appendages, but that can sometimes be difficult to see in poorly prepared material. In general, *L.schizocalyx* is found at higher elevations than *L.biflora*, but this is not consistent across the range of *L.biflora*. These two species may hybridise, but this has not been verified.

*Lyciantheslysimachioides* is sometimes confused with *L.schizocalyx*. The two species have similar calyx appendages; these reliably emerge below the rim in *L.lysimachioides* and often emerge below the rim in *L.schizocalyx*. *Lycianthesschizocalyx* can be distinguished from *L.lysimachioides* by its shrubby habit (versus prostrate herb rooting at the nodes), calyx appendages of variable length in a single flower (versus of equal length), and larger berries (1–1.3 cm versus 0.6–0.8 cm in diameter). In addition, *L.lysimachioides* usually has one-flowered inflorescences and leaves of a geminate pair often the same size and shape (appearing opposite). *Lycianthesshunningensis* also has 10 calyx appendages, but these are strongly reflexed; those of *L.schizocalyx* are erect or spreading.

Despite the difference in calyx appendage number *L.schizocalyx* (10 calyx appendages) can be very difficult to tell from *L.laevis* (5 calyx appendages) where they grow in sympatry. These two species appear to co-occur at the same locality in various parts of the species’ ranges (e.g., *Williams 1334* and *1334 [A*] from Philippines; *Kuntze 4574*, *4575* from Java). A specimen at NY purported to belong to the gathering *Williams 1334* (here distinguished as *Williams 1334 [A*], NY barcode 01404894) represents a plant of *L.schizocalyx*; this specimen is annotated as a duplicate of 1334 (“Dup. 1334”) in an unknown hand and lacks specific locality data. The other two specimens numbered *Williams 1334* are clearly *L.laevis* (see Suppl. material 2). The specimen of *L.schizocalyx* (NY barcode 01404894) may have been numbered a part of this gathering (*Williams 1334*) not by R.S. Williams, but by a herbarium curator at a later date. Numbers of calyx teeth are consistent but may be artificial, and population studies in areas where these plants co-occur are needed to really untangle this complex situation. The Vietnamese collection (*Croat & Dzu 77996*) cited by Hop (2017) as *L.neesiana* (here a synonym of *L.laevis*) is here identified as belonging to *L.schizocalyx*.

In describing S.biflorumvar.corynephorum, Kuntze (1891) stated only “Java: Tjibodas”; as lectotype for this name I have selected the better of the two specimens of *Kuntze 4574* with flowers and fruit in Kuntze’s herbarium held in NY (barcode 00172276) that is labelled with that locality and annotated as “var. corynephorum m” by Kuntze, along with an additional slip with details corresponding to the protologue.

Merrill (Merrill and Merritt 1910) cited several collections in the protologue of *S.schizocalyx*, indicating that *Merrill 4548* was the type, but did not cite a herbarium. I have selected the US duplicate of *Merrill 4548* (barcode 00027886, acc. # 71033) as the lectotype, as it is well-preserved, and Merrill was employed by the US government to work in the Philippines; the collections held in the Philippine National Herbarium were destroyed in World War II (Schultes 1957).

Bitter (1919) included the type of *S.schizocalyx* (*Merrill 4548*) in the syntypes cited for L.laevisvar.glabratula, at the same time recognising *L.schizocalyx* as distinct, along with *McGregor 8393*, both from Berlin (“hb. Berol.”). To avoid creating homotypic synonyms, I have designated *McGregor 8393* as the lectotype of var. glabratula, using the duplicate of this collection in the US National Herbarium (US 02840845, acc. # 628912). In his protologue for var. luzonensisBitter (1919) cited *Curran 5029* (a paratype of *S.schizocalyx*) and *Elmer 6565*, both from Berlin, calling *Curran 5029* the “Hauptform” (main form). The US duplicate of *Curran 5029* (strictly *Curran in Herb. Bur. Sci 5029*) is selected therefore as the lectotype of var. luzonensis (US 00027887, acc. # 708756). I have been unable to locate duplicates of the single collection cited (Bitter 1919) for L.laevisvar.majuscula (*Wichura 2168*); it is here placed in synonymy based on the description.

*Lycianthesbaviensis* was described (Hop 2004) citing as “Typus” the collection *HPP 136* held in the Hanoi herbarium (HN). Three specimens of the gathering *HPP 136* are held in HN, of these the one with the annotation as “type” and best corresponding to the line drawing in the protologue is selected as the lectotype.

### ﻿﻿10. Lycianthes shunningensis

Taxon classificationPlantae

C.Y. Wu & S.C.Huang, Acta Phytotax. Sin. 16(2): 77. 1978.

D89FDA12-F3F4-5DC6-B8AB-36912DB41337

**Figure 23.**
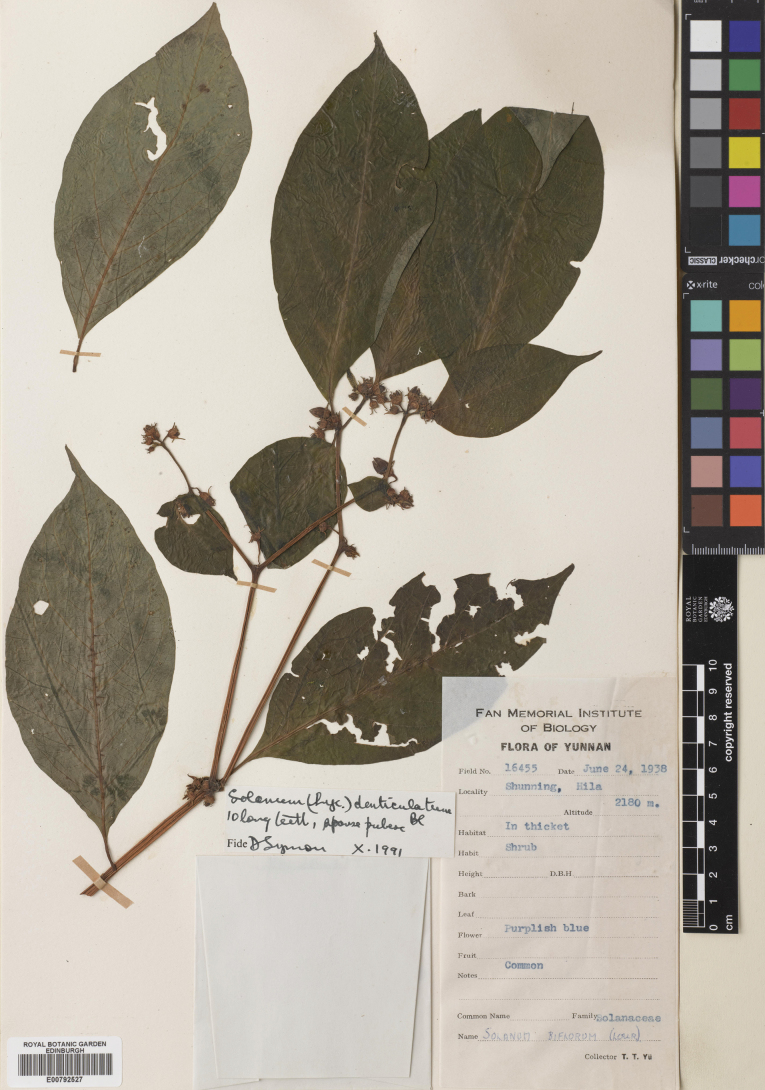
*Lycianthesshunningensis* C.Y.Wu & S.C.Huang herbarium specimen. China, Yunnan, *Yu 16455* (E00792527; isotype of *L.shunningensis*). Courtesy of Royal Botanic Garden, Edinburgh, reproduced with permission.

Figs 3F , 4F , 5F , 23

Lycianthes subtruncata (Dunal) Bitter var. remotidens Bitter, Abh. Naturwiss. Verein Bremen 24 [preprint]: 479. 1919. Type. China. Yunnan: “Szemao” [Pu’er, Simao District], 27 Aug [no year], *A. Henry 12352A* (lectotype, designated here: K [K001152508]; isolectotypes: B [destroyed], E [E00806597], MO [MO-503793, acc. # 52819], US [02840661, acc. # 459052]).

#### Type.

China. Yunnan: “Shunning, Hila” [Fenqing District, Shun Ning Lu], 2,180 m, 24 Jun 1938, *T.T. Yu [Yu Dejun] 16455* (holotype: KUN [acc. # 230263]; isotypes: A [00077123], E [E00792527], PE [00633443, 00633444]).

#### Description.

Shrubs or herbs, straggly or lax, sometimes described as climbing, ca. 1 m tall; stems terete, glabrous or sparsely pubescent with yellowish cream translucent simple uniseriate 2–8-celled trichomes to 1 mm long, soon glabrescent; new growth moderately to densely pubescent with translucent, simple uniseriate 2–8-celled trichomes to 1 mm long; bark of older stems glabrescent, yellow-green to greyish green. Sympodial units difoliate, the leaves geminate, the leaves of a pair differing in only size. Leaves simple; blades of major leaves 8–16 cm long, 2–6.5 cm wide, narrowly elliptic to elliptic, widest in the middle, concolorous, membranous; adaxial surfaces sparsely and evenly pubescent with simple uniseriate trichomes on the veins and lamina, the trichomes 2–3-celled, simple, uniseriate to 0.5 mm long, these often denser along the veins, the lamina always visible and the trichomes appearing depressed in dry specimens; abaxial surfaces glabrous or occasionally very sparsely pubescent with simple uniseriate trichomes like those of the adaxial surfaces, the midrib and principal veins with sparse pubescence; principal veins 6–8 pairs, sparsely pubescent or almost glabrous, the tertiary venation drying darker abaxially; base attenuate and somewhat decurrent onto the petiole; margins entire, ciliate with simple uniseriate trichomes to 0.5 mm long; apex acuminate; petiole 1.3–2.9 cm long, sparsely pubescent with simple uniseriate trichomes like those of the stems and new growth; blades of minor leaves 4–7.5 cm long, 1.5–3.5 cm wide, narrowly elliptic to elliptic; surfaces like those of the major leaves, the minor leaves often deciduous; principal veins of minor leaves 4–6 pairs; base attenuate; margins entire, usually somewhat ciliate; apex acuminate; petiole of minor leaves 0.6–0.8 cm long, sparsely pubescent like that of the major leaves. Inflorescences axillary, in fascicles, with 2–5(8) flowers, glabrous or with a few trichomes at the pedicel bases; pedicels at anthesis 0.8–1.1 cm long, 0.5–0.75 mm in diameter at the base, 1–1.5 mm in diameter at the apex, spreading, very sparsely pubescent with simple uniseriate trichomes like those of the stems, articulated at the base; pedicel scars tightly packed and overlapping. Buds ellipsoid, strongly tapered and pointed, the corolla never completely included, even in small buds slightly exserted, strongly exserted from the calyx tube before anthesis, the calyx appendages usually more apparent in bud. Flowers 5-merous, cosexual. Calyx tube 2–3 mm long, 2.5–4.5 mm wide at the mouth, an open cuplike structure, not ridged, glabrous or very sparsely pubescent with simple uniseriate trichomes like those of the pedicels, with 10 appendages arising ca. 1 mm below the hyaline rim, these more visible in bud (especially when the appendages are short), the appendages 0.2–4 mm long, ca. 0.5 mm wide, small nubs to curved and linear, strongly deflexed and parallel to the calyx tube, glabrous or very sparsely pubescent with a few simple uniseriate trichomes like those of the stems and pedicels. Corolla 1.1–1.3 cm in diameter, white to variously purple, stellate, lobed 3/4 or nearly to the base, interpetalar tissue absent but a thin edge of tissue apparent on lobe margins, the lobes 5–6 mm long, 2–2.5 mm wide, spreading, glabrous on both surfaces, the tips and margins densely papillate, the tips somewhat cucullate. Stamens equal; filament tube minute; free portion of the filaments 0.75–1 mm long, glabrous; anthers 2–2.5 mm long, ca. 1.25 mm wide, ellipsoid and tapering to a beak-like apex, tightly connivent, yellow, glabrous, poricidal at the tips, the pores tear-drop shaped and edged with white in dry material, lengthening to slits with age. Ovary conical, glabrous; style 5–6 mm long, exserted from the anther cone, glabrous; stigma prominently capitate, the surfaces minutely papillate. Fruit a globose berry, 0.8–1 cm in diameter, green when immature, bright red when mature, the pericarp glabrous, thin, shiny, and transparent at fruit maturity; fruiting pedicels 1.5–2 cm long, ca.1 mm in diameter at the base, ca. 2.5 mm in diameter at the apex, spreading or erect; fruiting calyx not accrescent or expanding, but remaining a plate-like structure, the appendages reflexed below berry in dry specimens, the appendages strongly reflexed and somewhat spreading. Seeds 20–40 per berry, 2–2.5 mm long, 1.5–2 mm wide, flattened-reniform or somewhat tear-drop shaped, pale straw-colored or yellow, the surfaces pitted only on the margins, the marginal testal cells rectangular to pentagonal, the lateral walls with “hairy” appendages ca. 0.2 mm long, the central testal cells sinuate. Stone cells absent. Chromosome number not known.

#### Distribution

**Figure 24.**
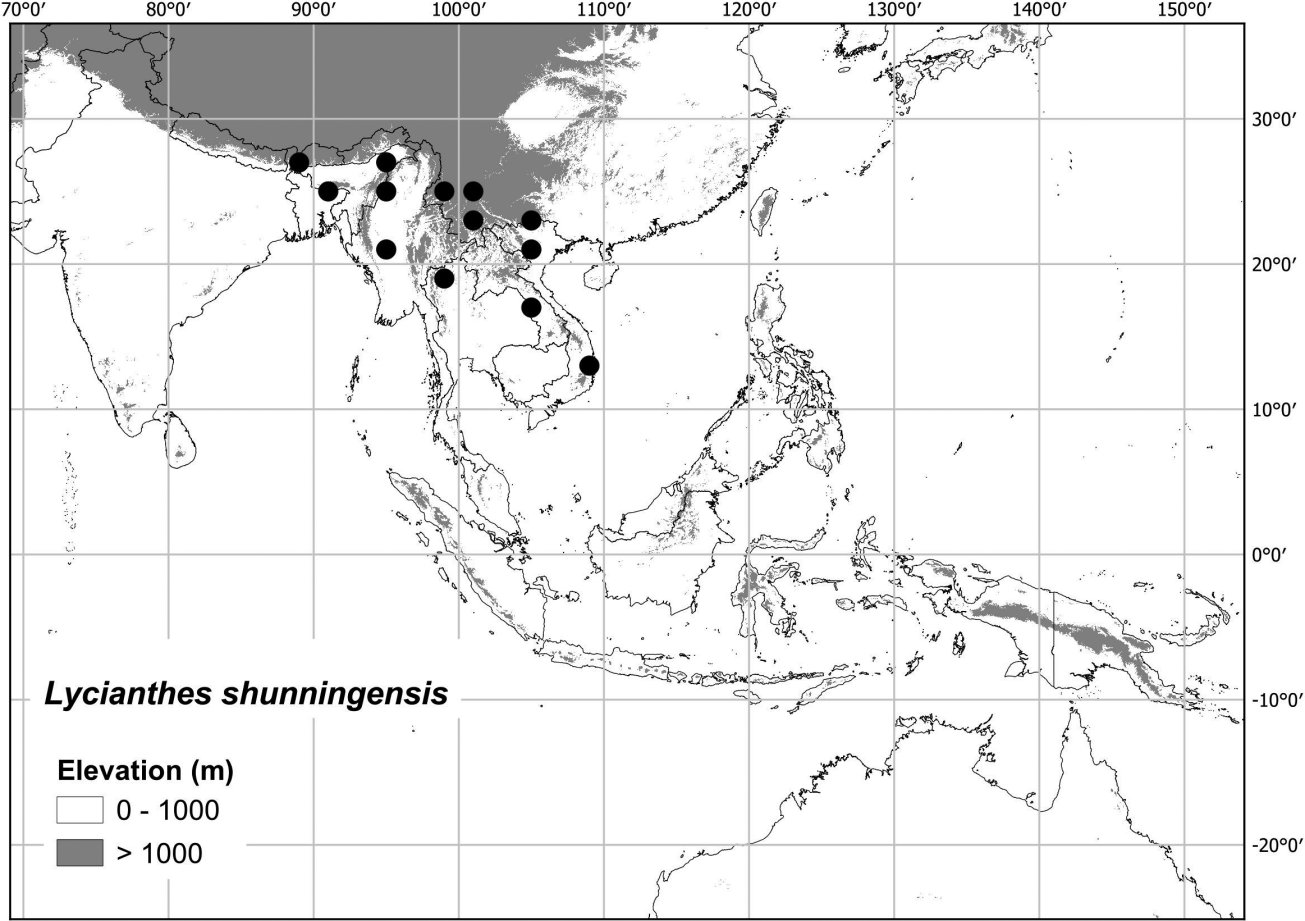
Distribution of *Lycianthesshunningensis*.

**(Fig. 24).***Lycianthesshunningensis* occurs in China, India, Laos, Myanmar (Burma), Thailand and Vietnam.

#### Ecology and habitat.

*Lycianthesshunningensis* grows in wet, evergreen forests, usually in the shaded understory, from 350 to 2,800 m elevation.

#### Common names.

China. jie chi hong si xian (as *L.neesiana*), shun ning hong si xian (Zhang et al. 1994). Vietnam. cà ngủ nees (as *L.neesiana*) (Hop 2017).

#### Preliminary conservation assessment

**(IUCN 2020).**EOO (1,388,419 km^2^ - LC); AOO (72 km^2^ - EN). *Lycianthesshunningensis* is known from ten localities and is somewhat widely distributed in the region. It is known from protected areas (e.g., Doi Inthanon National Park in Thailand, Ta Xua Nature Reserve in Vietnam). The assessment of Vulnerable (EN) based on AOO is likely due to collecting bias. I therefore assign it a preliminary status of Least Concern (LC).

#### Discussion.

*Lycianthesshunningensis* is recognised here with some hesitation, and the material included here may in fact belong to two different taxa. The specimens seen and grouped here all have strongly reflexed calyx appendages arising from usually more than 1 mm below the calyx rim (Figs 3F, 4F, 5F). This morphology is also seen in *L.connata* J.L.Gentry of Mexico and Guatemala (Dean et al. 2020: 76–78). The appendages in *L.shunningensis* vary in length, those on the type specimen (*Yu 16455*) from Yunnan in China and *Averyanov et al. AL887* from Laos (Figs 3F, 5F) are long and conspicuous, while those on the type of var. remotidens (*Henry 12352A*) and other collections are small nubs and quite inconspicuous (Fig. 4F); all, however, are strongly reflexed and arise well below the calyx rim. Variation in calyx appendage length is common in other Asian *Lycianthes* species (e.g. *L.laevis*, see description and discussion). On all of the specimens I have seen the leaf pubescence of *L.shunningensis* is also distinctive in being confined or nearly confined to the adaxial surface, most of the other taxa treated here when pubescent have denser pubescence on the abaxial leaf surfaces.

*Lycianthesshunningensis* occurs together with *L.biflora* (*Yu 16404*) at the type locality, and field studies of these populations would be useful in assessing the distinctness and consistency of the reflexed calyx appendage character that distinguishes *L.shunningensis*. A specimen of *L.biflora* (*Henry 12911*, US duplicate) was annotated as L.subtruncatavar.remotidens by Bitter in 1925, but it does not have the reflexed calyx appendages and otherwise conforms to my concept of *L.biflora* here. These *Lycianthes* species are very difficult to identify!

At least some of the material identified and treated as *L.neesiana* (here a synonym of *L.laevis*) by Zhang et al. (1994) and Hop (2017) corresponds to the type of L.subtruncatavar.remotidens with 10 reflexed calyx appendages; the type of *S.neesianum* (see under *L.laevis*) has 5 calyx appendages.

Lycianthessubtruncatavar.remotidens was described (Bitter 1919) based on *Meebold 7342* (from “hb. Berol., Vratisl.” – both destroyed, and no duplicates found), a collection from an unnamed collector (“Sammler des Bot. Gart. Calcutta!”) from Bogor, and *Henry 12352A* from “hb. Berol.” Nothing corresponding to the cited Bogor specimen has been found in BO. Bitter (1919: 480) distinguished the *Henry 12352A* collection as the basic form of the variety (“als Grundform dieser Varietät anzusehen!” and I have selected the Kew duplicate of this collection (K001152508) as the lectotype.

##### ﻿﻿Excluded taxa

***Solanumphilippinense* Merr., Philipp. J. Sci., Bot. 7: 351. 1912. Type. Philippines. Luzon: Cordillera (CAR) Mount Santo Tomas, 1 Jul 1904, *R.S. Williams 1275* (syntypes: K [K000759468], US [00027740, acc. # 707522]).**

= *Tubocapsicumanomalum* (Franch. & Sav.) Makino

**Note.**Merrill (1912: 352) suggested this species was “allied to *Solanumblumei* Nees” (= *L.laevis*). He cited several collections in this description and indicated that *Williams 1275* was the type, but without citing a herbarium. All the collections that Merrill (1912) cited that I have seen are referrable to *Tubocapsicumanomalum* (*Elmer 6561*, *Merrill 8003*, *8164*; I have not seen a duplicate of *Ramos 5406*).

##### ﻿﻿Names (designations) not validly published

Lycianthesbiflora(Lour.)Bittervar.glabra (Koidz. ex Hatus.) Hatus., Fl. Ryukus, ed. 2, 193. 1994, as “*biflorum* var. glabrum”. Not validly published, no complete citation of basionym place of publication (Art. 41.5, Turland et al. 2018). Based on SolanumbiflorumLour.var.glabrum Hatus. = *L.biflora* (Lour.) Bitter

*Solanumcrassipetalum* Wall., A numerical list of dried specimens of plants in the East India Company’s Museum 80. 1828–1849, nomen nudum. No description or diagnosis (Art. 38.1, Turland et al. 2018). “Wallich Cat.” 2618. = *L.laevis* (Dunal) Bitter

*Solanumdecemdentatum* Wall., A numerical list of dried specimens of plants in the East India Company’s Museum 80. 1828–1849, nomen nudum. No description or diagnosis (Art. 38.1, Turland et al. 2018). “Wallich Cat.” 2614. = *L.biflora* (Lour.) Bitter

*Solanumfloccosum* Zipp. ex Span., Linnaea 15(4) 337. 1841, nomen nudum. No description or diagnosis (Art 38.1, Turland et al. 2018). = *L.bimensis* (Miq.) Bitter.

*Solanumlysimachioides* Wall., A numerical list of dried specimens of plants in the East India Company’s Museum 80. 1828–1849, nomen nudum. No description or diagnosis (Art. 38.1, Turland et al. 2018). “Wallich Cat.” 2609. = *L.lysimachioides* (Wall.) Bitter

*Solanummacrodon* Wall., A numerical list of dried specimens of plants in the East India Company’s Museum 80. 1828–1849, nomen nudum. No description or diagnosis (Art. 38.1, Turland et al. 2018). “Wallich Cat.” 2621. = *L.biflora* (Lour.) Bitter

*Solanummembranaceum* Wall., A numerical list of dried specimens of plants in the East India Company’s Museum 81. 1828–1849, nomen nudum. No description or diagnosis (Art. 38.1, Turland et al. 2018). “Wallich Cat.” 2625. = *L.laevis* (Dunal) Bitter

*Solanumminahassae* Koord., Meded. Lands. Plantentuin 19: 547. 1989, nomen nudum. No description or diagnosis (Art. 38.1, Turland et al. 2018). Based on specimen labelled “*Solanumarboreum* Teysm. et Binn. msc. in H.H.B.” at Bogor (*Koorders 18030β*, *18049β*, *18152β* all annotated as “*Solanumarboreum* Teysm. et Binn.” at BO). = *L.banahaensis* (Elmer) Bitter

*Solanumsubtruncatum* Wall., A numerical list of dried specimens of plants in the East India Company’s Museum 80. 1828–1849, nomen nudum. No description or diagnosis (Art. 38.1, Turland et al. 2018). “Wallich Cat.” 2620. = *L.laevis* (Dunal) Bitter

SolanumurbanumMorongvar.typicum Chodat, Bull. Soc. Bot. Genève, ser. 2, 8: 151. 1916, not validly published. Use of prohibited epithet (Art. 24.3, Turland et al. 2018). = *L.rantonnetii* (Carrière) Bitter

## Supplementary Material

XML Treatment for Lycianthes

XML Treatment for Lycianthes banahaensis

XML Treatment for Lycianthes biflora

XML Treatment for Lycianthes bimensis

XML Treatment for Lycianthes laevis

XML Treatment for Lycianthes lysimachioides

XML Treatment for Lycianthes oliveriana

XML Treatment for Lycianthes parasitica

XML Treatment for Lycianthes rantonnetii

XML Treatment for Lycianthes schizocalyx

XML Treatment for Lycianthes shunningensis

## ﻿Additional information

### Author ORCIDs

Sandra Knapp https://orcid.org/0000-0001-7698-3945

### Data availability

All of the data that support the findings of this study are available in the main text or Supplementary Information.

## ﻿﻿Appendix 1

**Index to numbered collections**

For collections made by two or more collectors only primary (first listed) collector is presented here. Collections by anonymous collectors without date or other identifying features are not listed. Full collector strings can be found in the Supplementary Material searchable files (Suppl. materials 1, 2). This index and the searchable files are also available on the NHM Data Portal (https://doi.org/10.5519/xuvrw79j).

Aban Gibot SAN-66896 (*schizocalyx*); SAN-81013 (*biflora*); SAN-94332 (*laevis*).

Adduru, M 109 (*banahaensis*).

Aët 395 (*oliveriana*).

Afriastini, JJ 479 (*biflora*); 753 (*laevis*); 755 (*biflora*); 2859 (*schizocalyx*).

Alston, AHG 12898 (*biflora*); 14180 (*schizocalyx*); 14644 (*laevis*); 15100 (*schizocalyx*); 15692, 16218 (*biflora*.

Amano, T 6961 (*biflora*).

Amin, A SAN-117897 (*laevis*); SAN-118864 (*parasitica*); SAN-123497 (*schizocalyx*).

Amin, G SAN-93928 (*biflora*).

Ampon SAN-71804 (*biflora*).

An Mingtai 3916 (*lysimachioides*).

Andau, D 1118 (*biflora*).

Anderson, T 303, 364, 365 (*biflora*); 366 (*schizocalyx*); 1019, 1024, 1026 (*biflora*); 1029, 1030 (*laevis*).

Andrews, CW 181 (*biflora*).

Añonuevo, P 270, 13639 (*banahaensis*).

Argent, G 1047 (*schizocalyx*).

Armstrong, K 1036 (*biflora*); 1769 (*laevis*); 2187, 2966 (*biflora*).

Ashton, PS S.19162 (*biflora*).

Atkins, S 524 (*parasitica*).

Aung, MM 92543 (*biflora*); 92584 (*schizocalyx*).

Averyanov, L CBL-547 (*biflora*); AL-887 (*shunningensis*); VR-981 (*biflora*); CPC-8128, CPC-8219 (*schizocalyx*).

Backer, CA 3630 (*laevis*); 3654, 4658, 4845 (*biflora*); 5639 (*laevis*).

Bakar, A SAN-25023 (*parasitica*).

Bakia, A 612 (*schizocalyx*).

Balakrishnan, NP 1046 (*laevis*).

Balansa, B 3749 (*schizocalyx*); 3755 (*biflora*).

Balgooy, MMJ van 215, 3556, 3594, 4916 (*biflora*); 4935, 5015 (*banahaensis*).

Banang, E SAN-51907 (*parasitica*).

Barber, CA 2877, 2998 (*laevis*).

Barbon, EB PPI-5786 (*banahaensis*).

Bartlett, HH 8116a (*biflora*); 8178 (*parasitica*); 8215 (*biflora*); 8566, 8567, 8624 (*schizocalyx*).

Beaman, JH 6775, 7195, 8636, 9102 (*schizocalyx*); 9202 (*biflora*); 9554, 9780 (*schizocalyx*); 10953 (*biflora*); 10971, 11175, 11321 (*schizocalyx*).

Beccari, O 119 (*biflora*); 120 (*schizocalyx*); 714 (*laevis*).

Bechaman, A SAN-134488 (*laevis*).

Beddome, RH 5479, 5481, 5482, 5484, 5485, 5486, 5487 (*laevis*).

Bell, JR 7839 (*laevis*).

Bell, TRD 4253 (*laevis*).

Bembower, W 94, 297, 360 (*laevis*).

Bennet, SSR 3265 (*laevis*).

Berhaman, A 98 (*schizocalyx*).

Bernstein, JH 322 (*parasitica*).

Beusekom, CF van 1360, 3773 (*biflora*).

Biswas, K 98 (*biflora*); 4698 (*shunningensis*).

Biswas, K. 4578 (*shunningensis*).

Blatter, E 383[a] (*laevis*).

Bodinier, EM 485, 799 (*biflora*); 2903 (*lysimachioides*).

Boehmer, L 112 (*biflora*)

Bor, NL 4438, 6333 (*biflora*).

Boufford, DE 19409, 24197 (*lysimachioides*).

Bourell, M 2317 (*biflora*).

Bourne, AG 268, 471, 1129, 1130, 1131, 2088, 2808, 6256 (*laevis*).

Bourne, ET 1499 (*laevis*).

Boyce, PC 1004 (*shunningensis*); 1023 (*biflora*).

Brandis, D 350 (*laevis*).

Brass, LJ 23978 (*biflora*).

Britton, BB 192 (*parasitica*); 198 (*banahaensis*).

Brooke, WMA 9992, 10092 (*parasitica*); 10701 (*biflora*).

Bullock, AA 682 (*laevis*).

Bunnemeyer, HAB 2669 (*schizocalyx*); 3841 (*lysimachioides*); 4738 (*biflora*).

Burkill, IH 37463 (*shunningensis*); 37532 (*laevis*).

Burley, JS 2602 (*biflora*); 3214 (*parasitica*); 3611 (*biflora*); 3531 (*banahaensis*); 3628 (*biflora*).

Burtt, BL B-1880 (*biflora*).

Buwalda, P 6986 (*parasitica*).

Cai, X 56685 (*laevis*).

Calder, CC 422 (*laevis*).

Campbell, EFJ 312 (*parasitica*).

Canton Christian College 1882, 12648 (*biflora*)

Carr, CE 11670 (*oliveriana*); 14991 (*biflora*); 11670, 15948 (*oliveriana*); 15965 (*biflora*).

Cavalerie, PJ 1167 (*lysimachioides*);1360 (*biflora*).

Cave, GH 80 (*laevis*).

Cenabre, A 28511 (*banahaensis*).

Chai, P S.37360 (*biflora*).

Chamchumroon, V VC-5544 (*biflora*).

Chang, CE 8982 (*biflora*).

Chantaranothai, P 90/69, 90/469, 1160 (*biflora*).

Chatterjee’s collector 118 (*biflora*).

Chen Shaoqing 11848 (*lysimachioides*); 15058 (*lysimachioides*); 15217 (*laevis*); 15256 (*lysimachioides*); 15735, 16590 (*laevis*).

Chew, WL RSNB-126, 637, RSNB-1247, 1278 (*schizocalyx*); RSNB-4025 (*biflora*); RSNB-4707 (*schizocalyx*).

China Germany Team 492 (*laevis*); 1613 (*biflora*).

Ching, RC 1948, 5317, 6684 (*biflora*); 7526 (*laevis*).

Chou, CY 44 (*biflora*).

Chow, CL 6772 (*biflora*).

Chow, HC 685, 8077 (*lysimachioides*).

Chow, KS 79100, 80151 (*biflora*).

Christensen, H 71 (*biflora*).

Chun, NK 41918, 42333 (*biflora*).

Chun, WY 6764, 6764[b] (*biflora*).

Chung, HH 6743, 8025 (*biflora*).

Clarke, CB 7230, 8813 (*laevis*); 8830B (*biflora*); 8702 , 9224, 10901C, 10901A, 10956, 11083, 11486 (*laevis*); 12011, 12123, 12198 (*biflora*); 12265 (*lysimachioides*); 12304 (*laevis*); 12399, 12811A (*biflora*); 15063 (*laevis*); 15780A (*biflora*); 15891, 16514B, 16514A (*laevis*); 17850 (*lysimachioides*); 17865A, 18070B, 18901 (*laevis*); 18911, 18934 (*biflora*); 24781B, 25014 (*laevis*); 26728B, 26728D, 26845, 26879, 27004 (*biflora*); 27284B, 27284A (*laevis*); 27628A (*biflora*); 29781B (*laevis*); 35334A (*biflora*); 35855D (*laevis*); 36473A (*biflora*); 38718A, 38718D (*lysimachioides*); 40787A (*laevis*); 41074, 43496A, 43843, 44136E (*biflora*); 44567A (*laevis*).

Clemens, J 20171, 20624, 22188, 26049, 26119 (*schizocalyx*); 26139, 28470, 28479 (*biflora*); 31879, 32965, 40167, 40315, 50070 (*schizocalyx*).

Clemens, MS 939 (*banahaensis*); 1821 (*oliveriana*); 15792 (*banahaensis*); 16471 (*schizocalyx*).

Cockburn, PF SAN-83342 (*laevis*).

Cole, ? 54, 111 (*laevis*).

Collenette, IS 576, 21604 (*biflora*).

Collett, H 43 (*lysimachioides*).

Conklin, HC 861 (*laevis*).

Conn, BJ 1756 (*oliveriana*).

Coode, MJE 5277, 5372 (*banahaensis*).

Cooper, RE 467, 4466 (*biflora*); 4710 (*laevis*); 5941A, 6130 (*biflora*).

Copeland, EB 329 (*parasitica*).

Corner, EJH SF-32503 (*parasitica*).

Covile, DPM 6729 (*biflora*).

Craib, WG 66 (*biflora*); 360, 396 (*laevis*).

Cramer, LH 3458, 3801 (*laevis*).

Craven, LA 848 (*oliveriana*).

Creech, JL 599 (*biflora*).

Croat, TB 77996 (*schizocalyx*).

Cuming, H 837 (*parasitica*).

Cuong, NM 47, 662 (*biflora*).

Curran, HM 5029, 16201, 16203 (*schizocalyx*).

Curtis, C 1335 (*parasitica*).

D’Arcy, WG 19272 (*biflora*).

Daalen, GCE van 21 (*schizocalyx*).

Dai Tainlun 101068 (*biflora*).

Damas, D SAJ-1050 (*oliveriana*).

Darling, FW 14864 (*banahaensis*).

Dastur, RH 40 (*rantonnetii*).

Davidse, G 8647 (*laevis*).

Davis, PH 69213 (*schizocalyx*).

Deb, DB 156 (*laevis*); 746, 859 (*biflora*); 25735 (*laevis*); 26139, 26406 (*biflora*); 26996 (*laevis*); 27174 (*biflora*).

Deka, GK EC-5064 (*laevis*); 14008 (*lysimachioides*).

Denaga, M 29864 (*banahaensis*).

Deng Shiwei 90480 (*biflora*).

Diaoluo Shan Team 3029 (*biflora*).

Dobremez, JF 1753 (*lysimachioides*).

Docters van Leeuwen, WM 9884 (*oliveriana*); 11573 (*parasitica*)

Dransfield, J 6323 (*parasitica*); 6329, 6705 (*biflora*).

Du, D 418 (*lysimachioides*).

Du, HB HNK-2950 (*biflora*).

Du, NV HNK-2798, HNK-2950 (*biflora*).

Ducloux, F 736 (*lysimachioides*).

Dulong Jiang Investigation Team 230, 387 (*biflora*); 881, 1225, 2064 (*lysimachioides*)

Dunn, ST 3344 (*biflora*).

Duyag, A 76666 (*biflora*); 76679 (*banahaensis*).

East India Company 5903 (*biflora*).

Edaño, GE 223 (*parasitica*); 716, 852 (*biflora*); 2196 (*parasitica*); 2399 (*laevis*); 3322 (*banahaensis*).

Edinburgh Nepal Expedition 348 (*laevis*)

Elbert, J 2956 (*biflora*);4261 (*banahaensis*).

Elmer, ADE 6565 (*schizocalyx*); 7492(*banahaensis*); 7494(*parasitica*); 9208, 9425, 10489 (*banahaensis*); 10762 (*parasitica*); 11576 (*laevis*); 11693, 13782 (*banahaensis*); 13572 (*parasitica*); 13828 (*laevis*); 13881 (*biflora*); 13887 (*banahaensis*); 15895 (*parasitica*); 16996[b] (*biflora*); 20597 (*biflora*); 17592 (*laevis*).

Enggoh NBF-7345 (*parasitica*).

Erwin, ? S.27459 (*schizocalyx*).

Escritor, L 21306, 21445 (*banahaensis*); 21606 (*parasitica*).

Esquirol, JH 176, 523, 544 (*biflora*); 3257 (*lysimachioides*); 3662 (*biflora*).

Evangelista, B 883 (*parasitica*).

Everett, B FRI-13774 (*biflora*).

Expeditio Biologica Sino-Rossica 1236 (*biflora*)

Faber, E 138, 184, 190[b], 258, 279 (*lysimachioides*).

Fan, CS 272 (*lysimachioides*).

Fang, WP 2524, 2568 (*lysimachioides*); 2569, 15748, 17850 (*biflora*).

Faurie, U 318, 321, 324, 641 (*biflora*); 640, 918 (*lysimachioides*); 918[a], 1192, 1481, 4104 (*biflora*).

Feng, G 13227 (*laevis*).

Feng, KM 3061, 11392, 12923 (*biflora*); 13168 (*laevis*).

Fénix, E 3838 (*biflora*); 28181 (*parasitica*).

Field, WD 21y, 42 (*biflora*).

Fischer, CEC 417, 2220, 2741 (*laevis*).

Floto, F 7615 (*biflora*).

Floyd, AG 7459, 7509 (*biflora*).

Forbes, HO 507 (*biflora*); 586 (*parasitica*); 794, 882, 949, 974a, 995 (*biflora*); 1001C, 2034 (*schizocalyx*); 2224 (*laevis*); 3464 (*biflora*).

Ford, CA 124 (*biflora*).

Foreman, DB LAE-60242 (*biflora*).

Forrest, G 7898, 7972, 8125, 8650, 10590, 11035, 18237, 24674 (*biflora*); 27998 (*shunningensis*).

Fosberg, FR 37656, 37890 (*biflora*); 49975 (*laevis*).

Frodin, DG 2352 (*oliveriana*).

Frohne, G 57-127 (*parasitica*).

Fujikawa, K 53378 (*biflora*); 89302, 89469, 89521, 94109 (*schizocalyx*); 94293, 95404 (*biflora*).

Funakoshi, H 85366 (*shunningensis*).

Furuse, M 1789, 2503 (*biflora*); 3787 (*laevis*); 5629, 39791, 44780 (*biflora*).

Gachalian, FS 142 (*parasitica*).

Gaerlan, FJ PPI-137 (*biflora*); PPI-5238, PPI-5345, PPI-9910, PPI-10428, PPI-23561 (*banahaensis*); PPI-26149 (*parasitica*); PPI-26273 (*banahaensis*).

Gallatly, G 386 (*biflora*); 189 (*schizocalyx*).

Gamble, JS 741, 3396 (*biflora*); 3394A, 3395C (*laevis*); 9852 (*biflora*); 9928, 11452, 11604, 11712, 12335, 14821, 15636, 16276, 18034 (*laevis*).

Gamble, W 3393A, 3395A (*laevis*); 3400A (*lysimachioides*).

Gaoligong Shan Biodiversity Survey 15688, 15708, 15819, 16605, 18958, 20731, 20786, 22271, 22292, 26301, 27554, 28983, 32396, 32454, 33091, 33355, 33476, 33492, 33526 (*biflora*).

Gaoligong Shan Expedition 8709, 8727, 8829, 9728, 9758 (*biflora*)

Gardner, G 628 (*laevis*).

Garrett, HBG 1136, 1265 (*biflora*).

Gaudichaud, C 95 (*biflora*).

Geesink, R 7423 (*schizocalyx*); 8055 (*shunningensis*).

George, P SAN-138339 (*laevis*).

Gibbs, LS 3126 (*parasitica*)

Giking, M 236 (*biflora*).

Gilbert, MG 509 (*biflora*).

Girmansyah, D Deden-937 (*biflora*).

Gjellerup, K 613 (*oliveriana*).

Godefroy, A 139 (*biflora*).

Gressitt, JL 51, 269, 272 (*biflora*); 273, 449 (*lysimachioides*).

Grierson, AJC 2290 (*biflora*).

Griffith, W 296 (*laevis*); 760, 2331, 5903[a] (*biflora*); 5903[b], 5903 (*laevis*); 5903[c] (*lysimachioides*); 5904 (*laevis*).

Griswold, JA 68 (*schizocalyx*).

Guan Kejian 1724 (*lysimachioides*).

Hainan Station 340 (*biflora*).

Halim, MR EC-32553 (*lysimachioides*).

Hallier, H 24, 4476[a] (*biflora*).

Hamel, C 496 (*laevis*); 539, 668, 679 (*schizocalyx*); 1242 (*biflora*).

Hance, HF 2128 (*biflora*).

Handel-Mazzetti, HF 9043, 11164 (*lysimachioides*).

Hanoi-UBC-Logan-Longwood-Kew Expedition to Vietnam NHE-210 (*biflora*)

Hansen, B 12067 (*laevis*).

Hara, H 63-03549 (*laevis*); 63-03555 (*biflora*).

Harder, DK 1783, 1788 (*biflora*).

Hartless, AC 145 (*laevis*).

Hartley, TG 10065, 10136, 11428 (*oliveriana*).

Haviland, GD 1234 (*schizocalyx*); 1727 (*biflora*); 3711K (*parasitica*); 17217 (*biflora*).

Hayakawa’s collector N-415 (*biflora*).

Henderson, MR 18286, SF-22709, SF-22323 (*biflora*); SF-22378 (*lysimachioides*); SF-23365 (*schizocalyx*); SF-34225 (*laevis*).

Hendrian, R 810, 927 (*banahaensis*); 1005 (*lysimachioides*).

Heng, L 8727, 8829, 9728, 9758, 11476 (*biflora*).

Hennipman, E 6039 (*biflora*).

Henry, A 190 (*lysimachioides*); 307B, 307, 307A (*biflora*); 628 (*lysimachioides*); 758, 1750 (*biflora*); 3268 (*lysimachioides*); 4304 (*biflora*); 4757, 4762, 5912, 5912A, 6080, 6670, 7207 (*lysimachioides*); 9160, 9160A, 9218C, 9218B, 9218 (*biflora*); 10988 (*lysimachioides*); 12009, 12009A, 12009B, 12273 (*biflora*); 12352, 12352A (*shunningensis*); 12911, 13652 (*biflora*).

Henry, AN 61, SC-16387, SC-17439 (*laevis*).

Henty, EE NGF-29203 (*biflora*).

Herb Univ Hull Flora of India 281, 282, 283, 284 (*laevis*)

Herb. Griffith 5903 (*biflora*).

Herb. Harland 2128 (*biflora*).

Herb. Sampson 441A (*biflora*).

Herbal Community PT-783 (*biflora*).

Hicks, D 148, 150 (*banahaensis*).

Hiep, NT NTH-3420 (*biflora*).

Hochreutiner, BPG 825, 826, 1204, 1441 (*schizocalyx*); 1723 (*laevis*); 2041 (*schizocalyx*).

Hohenacker, RF 803, 1415 (*laevis*).

Hollrung, M 776 (*oliveriana*).

Holstvoogd, C 496 (*biflora*).

Hooker, JD 672 (*biflora*).

Hooper, D 38578, 38624 (*laevis*)

Hoover, WS Deden-50, Deden-300 (*laevis*); 809 (*biflora*); 30007 (*lysimachioides*); 30098 (*laevis*); 31106 (*biflora*); 30388, 30390, 30403 (*schizocalyx*); 31315, 31784 (*schizocalyx*); 31997, 32217, 32250, 32457, 32792 (*laevis*); 32477, 32632 (*schizocalyx*).

Hore, DK ANC-7553 (*biflora*).

Horsfield, T Sol.8, Sol.9, Sol.11part, 1421 (*laevis*); Sol.10 (*biflora*); Sol-11 (*schizocalyx*); Sol.11part, (*laevis*); Sol.13, 436 (*biflora*); 1421 (*laevis*).

Hose, C 119 (*parasitica*).

Hou Kuanzhao 73538 (*biflora*).

How, FC 70743, 72611, 73399, 73538 (*biflora*).

Hsiao, S-C 1240 (*biflora*).

Hu, SY 5523, 9277, 23888 (*biflora*).

Hu, WK 8921 (*biflora*).

Huang, SF 4486 (*biflora*).

Huang, TC 10675, 15179 (*biflora*).

Huang, YY 64 (*lysimachioides*).

Hugh, F 1699 (*lysimachioides*).

Idjan 348 (*oliveriana*).

Isles, S NGF-33899 (*biflora*).

Ismail, S BRF-1699 (*parasitica*).

Iwatsuki, K 516 (*biflora*); S-1487 (*lysimachioides*).

Jacob, KC 17555 (*laevis*).

Jacobs, M 4805 (*biflora*); 7070, 7367 (*schizocalyx*); 8073 (*lysimachioides*).

Jana, SK BSHC-17961 (*laevis*).

Jaswir SAN-30654 (*parasitica*).

Jawa, R S.70069 (*biflora*).

Jehen, A 7747b (*rantonnetii*).

Jenkins, F 244 (*biflora*); 246 (*laevis*).

Joamat 28177 (*schizocalyx*).

Johansson, JT 143 (*biflora*).

Joseph, J 15532, 39711 (*laevis*); EC-40453, 48294 (*biflora*).

Juan, GE 186 (*laevis*).

Junghuhn, FW 584 (*schizocalyx*).

Kairo, A 10652, NGF-27869 (*biflora*).

Kamarudin, S FRI-33752 (*schizocalyx*).

Kanai, H 63-03553 (*biflora*); 72-1160 (*lysimachioides*); 72-1682 (*laevis*).

Kanrtawinana, K 905 (*laevis*).

Kao, MT 6711, 7926 (*biflora*).

Kao, Y-C 387, 560 (*biflora*); 601 (*lysimachioides*).

Kartonegoro, A 606 (*laevis*).

Kato, H C-7273 (*biflora*).

Kaudern, WA 450 (*biflora*).

Kayal, RN 608 (*laevis*).

Keenan, J 809, 1124, 1521 (*biflora*).

Kerr, AFG 2799 (*shunningensis*); 3510, 5030, 5550, 6742, 7069 (*biflora*); 7529, 10820 (*schizocalyx*); 13293 (*biflora*); 21186 (*lysimachioides*);

Kessler, PJA 109 (*laevis*); 934 (*biflora*); 3014, 3038 (*biflora*); 3143 (*banahaensis*).

Kiew, R 4403 (*laevis*).

King’s collector 226 (*schizocalyx*); 2234 (*laevis*); 4709, 10260 (*parasitica*); 10930 (*laevis*).

King, G 209 (*laevis*); 210 (*biflora*); 733, 820 (*laevis*); 995 (*biflora*).

Kingdon-Ward, F 1823, 12849 (*lysimachioides*); 13884 (*biflora*); 17846, 18837 (*lysimachioides*); 22249 (*biflora*).

Kjellberg, G 1984 (*parasitica*).

Klackenberg, J 549 (*laevis*).

Kloss, CB SF-18977 (*parasitica*); SF-18984 (*biflora*).

Knapp, S 10106 (*biflora*).

Ko, SP 55822 (*biflora*).

Kobayashi, S 2811 (*biflora*).

Kochummen, KM FRI-16186 (*schizocalyx*).

Koelz, W 8780 (*rantonnetii*); 11211, 24352 (*laevis*); 25576, 26174 (*biflora*); 28282 (*laevis*); 29592 (*biflora*).

Kondo, Y 38792 (*parasitica*).

Konta, F T-29815 (*biflora*); T-29822 (*shunningensis*).

Koorders, SH 18031, 18038, 18041 (*biflora*); 18049B, 18050B, 18512B (*banahaensis*); 27862 (*laevis*); 27963B, 37847B (*biflora*); 40378 (*parasitica*).

Kornassi 649 (*oliveriana*).

Kostermans, AJGH 649, 671 (*banahaensis*); 777, 1024, 13995 (*biflora*); 264, 19054 (*bimensis*); 18398 (*laevis*); 2704 (*oliveriana*).

Koyama, H T-30451, T-31410 (*biflora*); T-61043 (*lysimachioides*); T-61226 (*laevis*).

Krishnappa, DG 400, 420 (*laevis*).

Krispinus, F SAN-120254 (*biflora*); SAN-128365 (*parasitica*).

Kunstler, H 226 (*schizocalyx*); 2234 (*laevis*).

Kuntze, CEO 4574 (*schizocalyx*); 4575, 4733, 5855, 6841 (*laevis*); 6007 (*biflora*).

Kuo, CM 8880 (*biflora*).

Kuroiwa, N 51266 (*schizocalyx*); 51333 (*biflora*).

Kurz, S 208 (*schizocalyx*); 455[a] (*biflora*); 455[b] (*lysimachioides*); 462 (*laevis*); 487, 487[b] (*lysimachioides*); 1502, 1775, 1777 (*biflora*); 1778 (*laevis*); 1803 (*biflora*); 2276, 2440 (*laevis*).

Lace, JH 4899, 6315 (*laevis*).

Lai, ST S.72457 (*schizocalyx*).

Laman, TG 347 (*parasitica*).

Lammers, TG 8506 (*biflora*); 8511 (*lysimachioides*).

Lamont, J 493 (*biflora*).

Lanjouw, J 71 (*laevis*).

Larsen, K 2393, (*schizocalyx*);.9007, 10427, 10513, 10516 (*biflora*); 30658 (*schizocalyx*); 34393 (*laevis*); 43707 (*schizocalyx*).

Lau, SK 4303, 4768, 20223, 25593, 28538 (*biflora*).

Lau, SY 20223 (*biflora*).

Lee, B S-52380 (*parasitica*).

Leong, WC 1287 (*lysimachioides*).

Leschenault de la Tour, JBL 311, 683 (*laevis*)

Leslie, JE 180 (*biflora*).

Leu, W-P 823, 886 (*biflora*).

Levine, CO 106, CCC-234, CCC-1652, 1882, CCC-3131, CCC-3458 (*biflora*).

Li Cehong 96062 (*biflora*).

Li Guangzhao 15380, 15971 (*biflora*).

Li Xuegen 204667 (*lysimachioides*).

Li Zexian 3189 (*lysimachioides*); 3594 (*biflora*).

Li Zhen-yu 361 (*lysimachioides*).

Liang, HY 64881 (*biflora*).

Liao, C-C 411 (*biflora*).

Lin, C-H 869 (*biflora*).

Lingnan University Herbarium 12648 (*biflora*)

Link, DA XI-105 (*biflora*).

Liou, TN 834 (*biflora*).

Liu Linhan 1979, 016358 (*lysimachioides*).

Liu Ying 275 (*lysimachioides*).

Liu, JH 442 (*biflora*).

Liu, TY 331 (*lysimachioides*).

Liu, YS 2134 (*lysimachioides*).

Loher, A 4374, 4375, 4376[b] (*schizocalyx*).

Long, DG 1057 (*biflora*).

Lörzing, JA 11366 (*biflora*); 13440 (*schizocalyx*); 14035 (*laevis*).

Lugas, L 2152 (*biflora*).

Luke, A 210 (*biflora*).

Lütjeharmes, WJ 5406 (*biflora*).

Mabesa, C 23812 (*banahaensis*); 24889 (*laevis*); 25381 (*banahaensis*).

MacGregor, RW 795 (*laevis*); 1302 (*biflora*).

Madani, L SAN-60131 (*schizocalyx*); SAN-92035 (*parasitica*); SAN-111183 (*laevis*).

Madulid, DA PPI-7756 (*banahaensis*).

Maire, EE 203 (*lysimachioides*).

Mamit, JD S.33474 (*biflora*).

Man, LS 55051, 55348, 87558, 88162, 96155 (*biflora*).

Mananduar, RK 6805 (*biflora*).

Mandal, NR BSHC-11037, BSHC-11132, BSHC-11823, BSHC-14672 (*biflora*).

Mann, G 136 (*biflora*).

Marcan, A 1156, 1398 (*biflora*).

Martinelo, A 35476 (*parasitica*).

Matthew, KM 16287, RHT-23335, RHT-24142, RHT-42486 (*laevis*).

Maxwell, JF 93-127, 09-262, 99-284, 05-456, 97-476, 06-534, 91-759, 94-866, 96-917, 89-1111, 93-1320 (*biflora*).

McClure, FA CCC-8268 (*biflora*); 9532 (*lysimachioides*); CCC-9699 (*biflora*).

McDonald, ? 4894 (*laevis*).

McGregor, MC 43766 (*parasitica*).

McGregor, RC 8393 (*schizocalyx*); 18510, 18853 (*parasitica*).

Mearns, EA BurSci4401 (*schizocalyx*); 4720 (*laevis*).

Meebold, A 6906 (*biflora*); 7057 (*laevis*); 7342 (*shunningensis*); 7867, 8878, 9456, 11600, 11696, 12409, 13926 (*laevis*); 17068 (*biflora*).

Meijer, W 2619 (*laevis*); 5337 (*lysimachioides*); 7242, 10445 (*laevis*); SAN-19246 (*parasitica*).

Mendoza, D 329 (*banahaensis*).

Mendoza, DR 61-210 (*parasitica*).

Merrill, ED 1719 (*biflora*); 3046 (*banahaensis*); 4548, 6588 (*schizocalyx*); 6157 (*parasitica*); 8266 (*schizocalyx*). 9525 (*biflora*).

Mgadiman SF-36779 (*biflora*).

Middleton, DJ 139, 3452 (*biflora*).

Mikage, M 96-85300, 9685300 (*lysimachioides*).

Millar, AN NGF-11795 (*biflora*); NGF-23858, NGF-35176 (*oliveriana*).

Millar, HN NGF-11794 (*biflora*).

Millard, AH KL-1792 (*laevis*).

Miyazaki, T 5080731/2 (*biflora*)

Mokim, S 46, 82, 116 (*laevis*).

Molino, J-F 3060 (*oliveriana*).

Moll, VW BW-9529 (*biflora*).

Monro, AK 6455, 6498 (*biflora*).

Mooney, HF 362, 1542 (*laevis*).

Motley, J 324 (*biflora*).

Muin Chai SAN-15531 (*laevis*).

Murata, G T-15042, T-16856, T-17789, T-17790, T-37073 (*biflora*).

Murata, J 24682 (*biflora*).

Muroi, H 2513 (*biflora*).

Murty, GGK 12992 (*laevis*).

Nanakorn, W 1090 (*biflora*).

Native Collector 2127 (*schizocalyx*).

Nees van Esenbeck, CGD 7271 (*laevis*)

Neth Ind Forest Service Cel./v300 (*banahaensis*).

NGS Hainan Expedition 509[B] (*biflora*); 509[A] (*lysimachioides*).

Nguyen, V.P. HPP-136 (*schizocalyx*).

Nguyen, VD HNK-1507 (*biflora*).

Nitta, A 15072 (*laevis*); 15090 (*schizocalyx*).

Nong Dongxin 451026141015017LY (*shunningensis*).

Nooteboom, HP 813 (*shunningensis*).

Nur, M SF-32658, SF-34225 (*laevis*).

Nuraliev, MS NUR-101n, NUR-182a (*biflora*); 1638 (*lysimachioides*); 2810 (*shunningensis*).

Nyugen, VD HNK-1507, HNK-2008 (*biflora*).

Oates, JF 105 (*laevis*).

Ocampo, M 27990 (*parasitica*).

Ohashi, H 775702 (*biflora*).

Okada, H 3284 (*biflora*).

Oldham, R 337 (*biflora*).

Ou, CH 9397 (*biflora*).

Palee, P 844, 995 (*biflora*).

Palmer, W 1185 (*laevis*).

Panigrahi, G 3843 (*lysimachioides*); EC-4047 (*laevis*); 4070 (*lysimachioides*); 4375, 5064 (*laevis*); 14531 (*biflora*); 17021 (*laevis*).

Parnell, J 95-091 (*biflora*); 95-120 (*schizocalyx*).

Parry, NE Mrs 282 (*schizocalyx*); 405, 826 (*laevis*).

Patsipun S.79958, S.82141 (*biflora*).

Pendry, CA 95-016 (*schizocalyx*).

Peng, C 8359 (*biflora*).

Peng, C-I 17304 (*lysimachioides*).

Peng, TC 13 (*lysimachioides*).

Pereira, JT 558 (*biflora*); SAN-151226 (*parasitica*).

Perrottet, GS 270, 518, 903[b] (*laevis*).

Pételot, A 2207, 3110 (*biflora*); 3167 (*lysimachioides*); 5459 (*schizocalyx*).; 8694 (*lysimachioides*).

Petrmitr, O 37 (*biflora*).

Phengklai, C 148 (*biflora*); 7194 (*laevis*)

Phuong, VX HNK-116, HNK-145, HNK-228 (*biflora*); HNK-653(*biflora*); HNK-622, HNK-797 (*laevis*); HNK-769 (*schizocalyx*); HNK-804 (*biflora*).

Pierre, L 635 (*biflora*).

Pleyte, DR 623 (*oliveriana*).

Poilane, E 17706 (*biflora*); 18985 (*laevis*).

Polak, AM 651, 864 (*oliveriana*).

Poore, MED H74, H501 (*biflora*).

Postar SAN-144127 (*parasitica*).

Powell, DA 164 (*biflora*).

Prain’s collector 225 (*laevis*).

Premanath, RK ANC-8464 (*biflora*).

Price, WR 299 (*biflora*); 832 (*lysimachioides*).

Puglisi, C LAOS89 (*laevis*); 139 (*biflora*).

Put Phraisurind 156 (*biflora*); 122 (*parasitica*).

Qin, H 89-3065, 89-3133 (*biflora*).

Qinghai-Tibet group (Expedition Xizang) 73-672 (*lysimachioides*)

Quisumbing, E 49-201, 5122 (*parasitica*).

Raghavan, RS WC-30179, WC-62492, WC-67850, WC-68101, WC-80513, WC-80785, WC-82976, WC-97282 (*laevis*).

Rahim SAN-100355 (*laevis*).

Rai, SK BSHC-21852, BSHC-23988, BSHC-25852, BSHC-38370, BSHC-38371 (*biflora*).

Rama Rao, M 1546 (*laevis*).

Ramamoorthy, TP HFP-1904, HFP-2029 (*laevis*).

Ramamurthy, K SC-78409 (*laevis*).

Ramesh, SR 3938 (*laevis*).

Ramlanto, ? 869 (*biflora*).

Ramos, M (Bur. Sci. #) 1463 (*parasitica*); 1646 (*banahaensis*); 4648 (*biflora*); 7252, 7637 (*banahaensis*); 20476 (*parasitica*); 23438 (*laevis*); 23536 (*banahaensis*); 24126 (*parasitica*); 27368, 27477 (*banahaensis*); 28989, 30271 (*parasitica*); 30357, 30519 (*biflora*); 30994, 31182 (*parasitica*); 32716, 33099, 34392, 34798, 34850, 34952 (*banahaensis*); 35027, 36650 (*parasitica*); 37551 (*banahaensis*); 37490, 37590 (*schizocalyx*); 7851 (*banahaensis*); 39430, 39456 (*laevis*); 40268, 40336, 40439, 40472 (*schizocalyx*); 40914 (*laevis*); 41058, 43308 (*parasitica*); 45017 (*biflora*); 46394 (*banahaensis*); 48403 (*biflora*); 48857, 48997, 75180, 85249 (*banahaensis*).

Rao, AS 48039 (*biflora*); 47745 (*laevis*).

Rao, RS 3955 (*lysimachioides*); 10400 (*biflora*); 10869 (*laevis*); 14052 (*lysimachioides*).

Rao, TA 826 (*biflora*).

Rau, K 380 (*biflora*).

Raynal, A 18863 (*schizocalyx*); 18881 (*laevis*); 18882 (*biflora*).

Reporter on Economic Products to the Govt. of India 11536 (*biflora*)

Rev St Munch 76 (*laevis*).

Reynoso, E PPI-3639 (*biflora*); PPI-24637 (*banahaensis*); .

Richards, PW 2622 (*biflora*).

Ridley, HN 34 (*biflora*); 7448 (*laevis*); 8211 (*schizocalyx*); 8562, 9714 (*laevis*); 10688 (*schizocalyx*); 12210 (*parasitica*); 13565, 13566 (*laevis*).

Ritchie, C 1301 (*laevis*).

Robinson, HC 5 (*schizocalyx*). 34 (*lysimachioides*); 61 (*schizocalyx*); 62, 82 (*laevis*); 116 (*schizocalyx*).

Rock, JF 226 (*shunningensis*); 1706 (*biflora*).

Rodger, A 187 (*biflora*).

Rogers, CG 324T (*lysimachioides*).

Romero, E PPI-29539 (*banahaensis*).

Rosenbluth, R 12675 (*banahaensis*)

Royen, P van 7621, 7716 (*oliveriana*); 10682 (*laevis*).

Rup Chand, T 2572 (*laevis*); 315 (*biflora*); 3698, 3789, 5711 (*laevis*).

Ruse, LF 141 (*biflora*).

Russell, PT 2166, 2219 (*biflora*).

Ryan, GM 1495 (*laevis*).

Safni, B 1834 (*laevis*).

Sahoo, AK BSHC-26696 (*lysimachioides*).

Saigol, P SAN-93078 (*laevis*).

Saito, S 7294 (*biflora*).

Saldanha, CJ HFP-409 (*laevis*).

Salvoza, F 29642 (*banahaensis*).

Sampson, T 441 (*biflora*).

Sands, MJS 272 (*biflora*); 507 (*laevis*); 6431 (*biflora*); 6744 (*oliveriana*); 6791 (*biflora*).

Santapau, H 11900, 11901, 11945 (*laevis*).

Santos, JK 32023 (*schizocalyx*).

Santos, JV 4207 (*biflora*).

Sapiin 2473 (*schizocalyx*).

Sastry, ARK 12411 (*laevis*).

Saulière, A 3, 22, 72, 73, 627 (*laevis*).

Schiffner, V 2505 (*laevis*); 2512 (*schizocalyx*).

Schlechter, FRR 13748, 13749a, 17305 (*biflora*); 17961, 18319, 18427, 20256 (*oliveriana*).

Schram, FAW BW-10645, BW-10744 (*biflora*).

Scortechini, B 149b (*parasitica*); 1221 (*laevis*).

Sebastine, KM 1018 (*laevis*).

Sedgwick, LJ 2909, 7037 (*laevis*).

Sengupta, G 354 (*biflora*); 871 (*laevis*).

Shah, M 695 (*laevis*).

Shin Ying Hu 5523 (*biflora*).

Shukla, BK BSHC-21022 (*biflora*).

Si Boeea, R 5813, 6213, 6246 (*parasitica*); 6619 (*biflora*); 7082, 7083 (*laevis*); 7798, 8613 (*parasitica*); 8649, 8765, 9034 (*biflora*); 9081, 9379, 9659 (*parasitica*); 10182 (*schizocalyx*); 10232 (*biflora*); 10242, 10323, 10418, 10563, 10641, 10780, 10971 (*schizocalyx*).

Sidiyasa, K 2124A (*biflora*).

Simpson, DA 2578 (*parasitica*).

Sinclair, J 6103 (*laevis*); 8147, 9347 (*parasitica*); 9761 (*biflora*); SF-38711 (*laevis*).

Sino-American Expedition 617 (*lysimachioides*)

Sino-American Guizhou Botanical Expedition 1240 (*lysimachioides*)

Siti Munirah, MY FRI-76680 (*biflora*).

Smith, H 10068 (*lysimachioides*).

Smith, WW 39, 514 (*laevis*).

Society of Himalayan Botany Expedition to Nepal 94-60089 (*laevis*)

Soedarsono 330 (*parasitica*).

Soejarto, D 10406 (*laevis*).

Soewarta 143 (*biflora*).

Soibeh, D 789 (*schizocalyx*).

Solomon, JC 20620 (*biflora*); 21218 (*lysimachioides*).

Song Xianghou 929 (*biflora*)

Sorenson, T 3730 (*biflora*).

Srinivasan, SR SC-65950 (*laevis*).

Srivastava, RC BSHC-10301 (*biflora*).

Stainton, JDA 3983, 6077 (*laevis*); 6505 (*lysimachioides*); 8809 (*laevis*).

Steenis, CGGJ van 6212 (*schizocalyx*); 6457 (*lysimachioides*); 7310 (*biflora*); 8085 (*lysimachioides*); 8124 (*laevis*); 9354 (*schizocalyx*); 9373 (*lysimachioides*); 10816 (*schizocalyx*).

Steiner, ML 2072 (*schizocalyx*).

Stevens, PF LAE-58668 (*biflora*).

Steward, AN 130 (*lysimachioides*); 605 (*biflora*); 683 (*lysimachioides*).

Strachey, R 3 (*lysimachioides*).

Streimann, H NGF-25853 (*biflora*).

Subba Rao, GV 23245 (*laevis*).

Subramanian, KN 1914, 9510 (*laevis*).

Sugau, JB SAN-147090 (*biflora*).

Suksathan, P 1841 (*shunningensis*).

Sulit, MD 1340, 1656 (*laevis*); 2036, 4383 (*parasitica*); 8498 (*banahaensis*); 9852, 14570 (*parasitica*); 16964 (*biflora*); 29448 (*banahaensis*).

Sumbing Jimpin SAN-113836 (*biflora*).

Sun, CL 743 (*lysimachioides*).

Sundaling, P SAN-71284 (*biflora*).

Surunda, Y 100 (*laevis*).

Suzuki, S 275, 4989, 5039, 10659 (*lysimachioides*).

Sykes, WR Ch-63 (*biflora*).

Symington, CF 27120 (*laevis*).

Symon, DE 10655, 10659, 13896 (*oliveriana*).

Taam, YW 2053, 2503 (*biflora*).

Tadong, D 506 (*schizocalyx*).

Tai, LY T-453 (*biflora*).

Takahashi, H T-62628 (*schizocalyx*); T-63382 (*biflora*).

Takeuchi, WN 6156, 9307 (*oliveriana*); 12688, 16902 (*biflora*); 17743, 17786, 22112, 23389, 23491, 23895 (*oliveriana*).

Talangamin, F 51 (*banahaensis*).

Talbot, WA 123, 653, 3007 (*laevis*).

Tamura, M 21574 (*lysimachioides*).

Tanaka, N 23001, MY-338 (*biflora*); 23235 (*schizocalyx*); 23525, 30626 (*biflora*); 81320 (*schizocalyx*).

Tanaka, T 341, 17877 (*biflora*).

Tang Siu Ging 5699, 7094, 7163, 16101 (*biflora*).

Teijsmann, JE 8918 (*banahaensis*).

Teng, SW 90828 (*biflora*).

Tessier-Yamdell 35 (*biflora*).

Thorel, C 2087 (*biflora*).

Thothathri, K 10551 (*laevis*).

Thwaites, GHK 1900, 2429 (*laevis*).

Tibet-MacArthur 2111 (*biflora*).

Toppin, S 4187 (*laevis*).

Treutler, WJ 238 (*biflora*).

Tsai, HT 52287 (*lysimachioides*); 52494, 54160, 54273, 58534, 61720, 61940 (*biflora*).

Tsang, WT 355, 540, 21464, 28144 (*biflora*).

Tsiang, Y 419, 4621 (*biflora*); 6719, 7669, 7852, 8806 (*lysimachioides*); 9256, 9505, 12428 (*biflora*).

Tsui, TM 411, 422, 827 (*biflora*).

Uji, T 2750 (*biflora*).

University of San Carlos 194 (*banahaensis*); 379 (*parasitica*)

Uong Sing Po 12053 (*biflora*).

USA Typhus Commission 526 (*laevis*)

Utteridge, TMA 287, 295 (*oliveriana*).

Vajravelu, E SC-29245 (*laevis*).

Vidal, S 3349 (*laevis*); 3367 (*banahaensis*).

Vinas, A LAE-59477 (*oliveriana*).

Vink, W 16847 (*oliveriana*).

Vogel, EF de 1326, 3565, 3718 (*biflora*); 5600 (*banahaensis*).

Voogd, CNA de 972 (*biflora*); 2819 (*bimensis*); 3119 (*laevis*).

Walker 65 (*laevis*).

Walker, EH 5876, 7608, 8336 (*biflora*).

Walker, GW 97, 107 (*laevis*); 236 (*biflora*); 333 (*laevis*).

Wallich, N Cat.2614[A], Cat.2621 (*biflora*); Cat.2618, Cat.2620, Cat.2625A, Cat.2625B, Cat.2625b, Cat.2625C, Cat. suppl.248 (*laevis*); Cat.2609 (*lysimachioides*)

Walsh, ME 99 (*biflora*).

Wang Congrong 1355, 97341 (*lysimachioides*).

Wang Qiansheng 2539 (*lysimachioides*).

Wang Xuewen 8233 (*biflora*).

Wang, C 32543, 33638(*biflora*); 35210 (*laevis*); 36950(*biflora*); 39993 (*laevis*); 40596, 40894, 42589 (*biflora*).

Wang, C-C 544 (*biflora*).

Wang, C-M 2294 (*lysimachioides*).

Wang, CW 72647, 76510, 76822, 78209 (*biflora*).

Wang, FT 23303 (*lysimachioides*).

Wang, J-C 2693, 2781 (*biflora*).

Wang, TP 857 (*lysimachioides*).

Wang, WH 8113 (*biflora*).

Warburg, O 4186(*biflora*); 14349 (*banahaensis*); 14354 (*parasitica*); 15066, 15067 (*biflora*); 15074[b] (*banahaensis*); 21250 (*biflora*).

Watt, G 5647 (*biflora*); 6683, 6691 (*laevis*).

Weber, CM 1207 (*parasitica*).

Wells, J NGF-7569 (*biflora*).

Wen, J 7373, 7374, 10282 (*biflora*).

Wenzel, CA 219 (*banahaensis*); 500, 3091 (*parasitica*).

Widjaja, EA 8917, 9734 (*biflora*).

Wight, R 1569-105 (*biflora*); 582, 691, 1569, 1569[a], 1569-103, 1569[2417], 1569-101, 1569-127, 1569b/105, 2012[b], 2012, 2021, 2024, 2025[a], 2025 (*laevis*).

Wilde, JJFE de SAN-143905 (*parasitica*).

Wilde, WJJO de 12173, 12397 (*parasitica*); 12445 (*lysimachioides*); 13655 (*schizocalyx*); 14870 (*parasitica*); 16685 (*schizocalyx*); 18076 (*parasitica*); 18335 (*schizocalyx*).

Williams, LHJ 8144 (*biflora*); 8330 (*laevis*).

Williams, RS 1334 (*laevis*); 1334 [A] (*schizocalyx*); 2170 (*parasitica*); 2300, 2334, 2760 (*banahaensis*).

Wilson, EH 1374, 2158, 4201 (*lysimachioides*); 4202 (*biflora*); 5095 (*lysimachioides*); 5096 (*biflora*).

Winkler, HJP 2701 (*parasitica*).

Wiriadinata, H 10634 (*laevis*).

Without Collector 6, 26 (*laevis*); 40, 85 (*biflora*); 161, 194 (*laevis*); 330, 459 (*biflora*); 755, 1448 (*laevis*); 2188 (*shunningensis*); 3007, 11557 (*laevis*).

Womersley, JS NGF-37289 (*oliveriana*)

Woodrow, GW 209 (*laevis*).

Worthington, RD 13340 (*schizocalyx*).

Wray, L 1460 (*laevis*); 3409, 3968 (*parasitica*).

Wright, C 197 (*biflora*).

Wu, MJ 1331 (*biflora*).

Wu, S-H 1053 (*biflora*).

Xiong, J 32197 (*lysimachioides*).

Xu Honggui 4615, 5138 (*lysimachioides*).

Yahara, T 7431 (*biflora*).

Yamamoto, Y 1150 (*biflora*); 2430 (*lysimachioides*).

Yao, K 9354 (*lysimachioides*).

Yates, HS 2864 (*schizocalyx*); 2994 (*laevis*); 3038 (*biflora*).

Yen, HF 8400 (*biflora*).

Yii S.70379 (*biflora*).

Ying, T 419 (*biflora*).

Yonekura, K 11397 (*biflora*).

Yu, R-Y 118 (*laevis*).

Yu, TT 16404 (*biflora*); 17009 (*lysimachioides*); 16455 (*shunningensis*); 20377 (*biflora*).

Zhang Guicai 369 (*lysimachioides*).

Zhang, T 10CS-2040 (*biflora*); 10CS-2128 (*lysimachioides*).

Ziyunshan Expedition 1607 (*lysimachioides*).

Zollinger, H 705 (*lysimachioides*); 705[a], 723, 1256 (*biflora*); 1262 (*laevis*); 1759 (*parasitica*); 1799, 1981, 1982 (*biflora*); 2597, 2597Z, 2597bis (*laevis*); 3458 (*bimensis*).

Zwickey, AL 163 (*biflora*); 670 (*banahaensis*).

## Supplementary materials

﻿Supplementary material 1 Searchable csv file of all collection events (observations and specimens) for *Lycianthes* outside of the Americas. Data type csv Explanation note This file can also be found on the Natural History Museum’s Data Portal (https://doi.org/10.5519/xuvrw79j). https://binary.pensoft.net/file/1103819This dataset is made available under the Open Database License (http://opendatacommons.org/licenses/odbl/1.0/). The Open Database License (ODbL) is a license agreement intended to allow users to freely share, modify, and use this Dataset while maintaining this same freedom for others, provided that the original source and author(s) are credited.Sandra Knapp

Supplementary material 2 Searchable csv file of *Lycianthes* collections seen for this revision (including *L.rantonnetii* over its entire range) Data type csv Explanation note This file can also be found on the Natural History Museum’s Data Portal (https://doi.org/10.5519/xuvrw79j). https://binary.pensoft.net/file/1103820This dataset is made available under the Open Database License (http://opendatacommons.org/licenses/odbl/1.0/). The Open Database License (ODbL) is a license agreement intended to allow users to freely share, modify, and use this Dataset while maintaining this same freedom for others, provided that the original source and author(s) are credited.Sandra Knapp
